# A Straightforward Synthesis of Polyketides via Ester Dienolate Matteson Homologation

**DOI:** 10.1002/chem.202004650

**Published:** 2020-12-15

**Authors:** Oliver Andler, Uli Kazmaier

**Affiliations:** ^1^ Institut für Organische Chemie Universität des Saarlandes Campus C4.2 66123 Saarbrücken Germany

**Keywords:** boronic esters, enolates, Matteson homologation, natural products, polyketides

## Abstract

Application of ester dienolates as nucleophiles in Matteson homologations allows for the stereoselective synthesis of highly substituted α,β‐unsaturated δ‐hydroxy carboxyl acids, structural motifs widespread found in polyketide natural products. The protocol is rather flexible and permits the introduction of substituents and functionalities also at those positions which are not accessible by the commonly used aldol reaction. Therefore, this ester dienolate Matteson approach is an interesting alternative to the “classical” polyketide syntheses.

## Graphical abstract

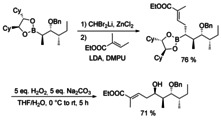

Polyketides are an enormously large group of natural products found widespread in linear as well as in cyclic form as macrocycles.^[1]^ Biosynthetically, the polyketides are formed via decarboxylative Claisen condensation of activated malonate, followed by reduction of the β‐ketoester formed.^[2]^ Therefore, classical polyketides often contain *O*‐functionalities at 3,5,7 positions. Double bonds can be obtained via dehydration, and their reduction gives rise to linear unsubstituted carbon chains, comparable to fatty acids. The use of activated methyl malonate results in the introduction of methyl groups at positions 2,4,6 of the polyketide chains, while anomalous substitution pattern are generally the result of post‐translational modifications.^[3]^ The combination of the involved polyketide synthases (PKS) with modules of nonribosomal peptide synthetases (NRPS) gives access to peptide‐polyketide hybrids^[4]^ such as jasplakinolide,^[5]^ miuraenamide^[6]^ or lagunamide^[7]^ (Figure 1), to name only a few. Many of these natural products have highly interesting biological activities and might be good candidates for the development of new antibiotics or anticancer agents.^[8]^ Therefore, it is not surprising that many efforts has been undertaken in the development of asymmetric syntheses of these natural products.^[9]^

**Figure 1:**
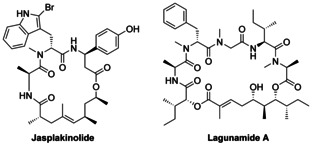
Natural occurring peptide polyketide hybrids.

The most popular approaches take advantage of asymmetric aldol reactions, in all their variations, to generate directly the polyketide substitution pattern.^[10]^ An alternative approach uses asymmetric allylations/crotylations in combination with oxidative double bond cleavage to generate the same structural motifs.^[11]^ No question, these protocols are straightforward for the syntheses of classical polyketides, but are less suited for posttranslational modified natural products, having O‐ or C‐functionalities at “unusual” positions.

Our group is also involved in the synthesis of biological active natural products, focusing on cyclic peptides and peptide‐polyketide hybrids.^[12]^ To become independent of the aldol‐motif we developed recently a synthesis of lagunamide based on a Matteson homologation.^[13]^ This stereoselective prolongation of chiral boronic esters was introduced by Donald Matteson already 40 years ago.^[14]^ Key step of this protocol is the highly stereoselective formation of a α‐chloro boronic ester **A** (Scheme 1) which can be subjected to nucleophilic substitution under S_N_2‐conditions with a wide range of nucleophiles,^[15]^ such as Grignard reagents, alkoxides or certain enolates.[^16^, ^17^] This allows for the stepwise stereoselective incorporation of substituents and functionalities into a growing carbon chain. For a long time most synthetic applications of this elegant method were reported by Matteson himself,^[18]^ but in the last years it found several nice applications in natural product^[19]^ or drug syntheses.^[20]^ Aggarwal developed a version where the stereochemical outcome of each prolongation step can be controlled by using spartein as a chiral ligand.^[ 15b,  21]^

**Scheme 1:**
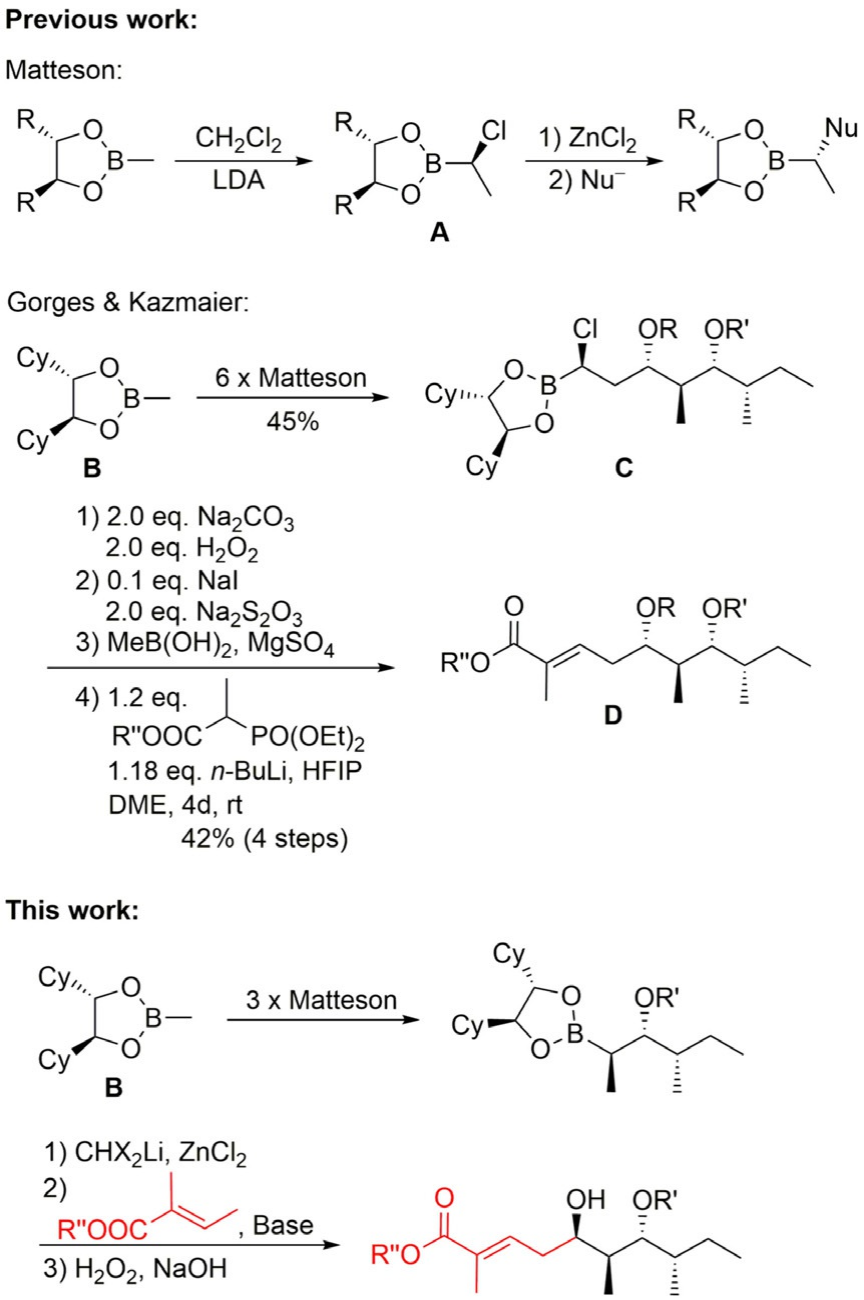
Matteson homologation and application.

We applied the classical Matteson approach in the synthesis of the polyketide fragment of lagunamide A from ester **B**,^[13]^ where all four stereogenic centers were controlled by the chiral diol in the boronic ester. Key intermediate was the prolonged α‐chloro boronic ester **C** which was oxidized to the corresponding aldehyde and subjected to a Horner–Wadsworth–Emmons olefination^[22]^ to the desired α‐methylated α,β‐unsaturated ester **D**. Unfortunately, this last step was rather slow, requiring 4 days for completeness and providing a 4:1 (*E*/*Z*)‐mixture of the α,β‐unsaturated ester.

Therefore, we tried to shorten the synthetic sequence significantly with the option to get also access to other stereoisomers for SAR studies. The idea was to stop the Matteson homologation sequence after generation of the first three stereogenic centers and to introduce the “non chiral” part (red) of the polyketide in one step, including the α,ß‐unsaturated ester moiety. In this case the OH‐group is not introduced via S_N_2 reaction but by oxidation of the boronic ester and should therefore be obtained with the opposite configuration as before, what makes this approach an interesting complement to the previous protocol. As nucleophiles we wanted to use deprotonated butenoic esters, vinylogous enolates which have been used previously in aldol additions.

In comparison to normal ester enolates, reactions of the vinylogeous enolates are more difficult to control, because besides α‐ and γ‐ products also (*E*/*Z*)‐isomers of the resulting double bond can be obtained and it is sometimes difficult to separate the regio‐ and stereoisomers. While alkylations preferentially occur at the α‐position,^[23]^ the outcome of allylations depends on the counter‐ion of the dienolate.^[24]^ Li‐Dienolates also give rise to α‐products, the γ‐products can be obtained preferentially with Cu‐dienolates. Kinetically controlled aldol reactions yield mainly the α‐product,^[25]^ while the γ‐products are obtained under thermodynamical conditions.[^26^, ^27^] Mukaiyama aldol reactions using vinylogeous silylketenacetals as nucleophiles also give a higher ratio of γ‐substitution product,^[28]^ while this approach was used frequently in polyketide syntheses,^[29]^ for example, in a synthesis of lagunamide A and related compounds.^[30]^ To the best of our knowledge dienolates have never been applied in Matteson homologations so far, and only two publications describe the use of simple ester enolates.^[16]^

To figure out if the dienolates can be used in Matteson homologations at all we investigated the reaction of crotylesters with phenyl ethyl boronic ester **1** (Table 1). First experiments were carried out under “typical Matteson conditions” using CHCl_2_Li as a nucleophile. Crotonic esters cannot be deprotonated with LDA, because 1,4‐addition of the amide is a too fast process,^[29a]^ but this side reaction can be suppressed by addition of HMPA.^[23]^ To avoid the usage of this nasty reagent we decided to use DMPU (*N*,*N*'‐dimethylpropylene urea) instead.^[31]^ But no desired homologation product could be observed, only a mixture of undefined products. We assumed, that the reactivity of the α‐chloro boronic ester might not be high enough and decided to switch to the corresponding α‐bromo derivative. These brominated esters are more reactive, but show also a higher tendency for epimerization, causing products with lower stereoselectivity.^[32]^ Therefore, it is recommended to use such esters without purification and storage to avoid decomposition and epimerization. With α‐bromo boronic ester **2**, the desired product could be obtained in acceptable yield, although as a mixture of the linear (*E*)‐configured γ‐product (**3 a**) and the branched α‐product (**3 a'**) (as diastereomeric mixture) (Table 1, entry 1). After oxidation, the desired δ‐hydroxylated unsaturated ester could be obtained in pure form. Ethyl crotonate gave almost the same result as the *tert*‐butylester (entry 2). No reaction was observed with silylketenacetals, even in the presence of Lewis acids. Therefore, we decided to investigate the influence of the counter ion, which has an influence in aldol reactions.^[24]^ But in our case the addition of copper salts had no influence on the α/γ‐selectivity, only the yield dropped (entries 3 and 4). In the presence of magnesium salts no complete conversion could be observed (entry 5) and in all cases more or less 1:1 mixtures of the regioisomers were obtained.

**Table 1:** Matteson homologations of **1** using crotonic ester dienolates.

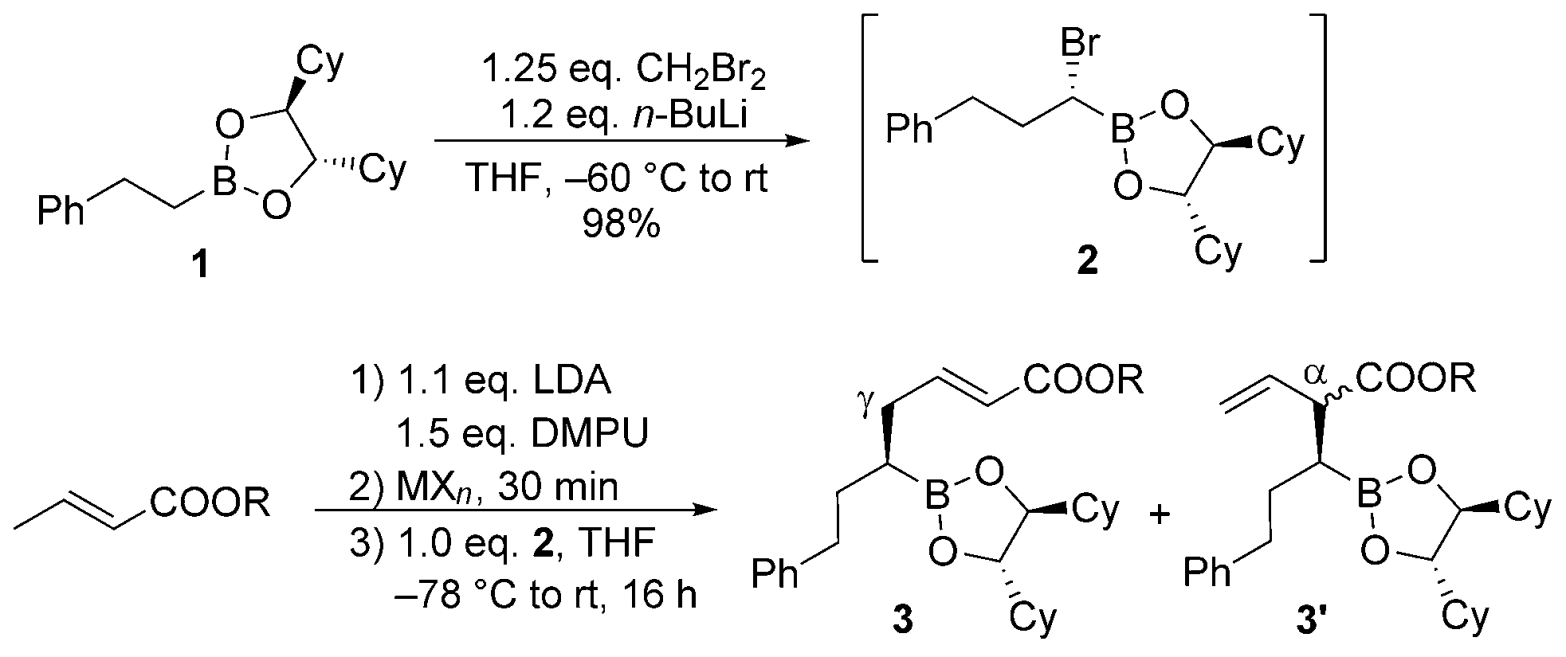					
Entry	R	MX_*n*_ (eq.)	Yield [%]	Product	Ratio **3**:**3**’
1	*t*‐Bu	–	65	**3 a**	57:43
2	Et	–	54	**3 b**	63:37
3	*t*‐Bu	CuI (1.2)	48	**3 a**	43:57
4	*t*‐Bu	CuBr⋅SMe_2_ (1.0)	53	**3 a**	56:44
5	*t*‐Bu	MgBr_2_⋅OEt_2_ (2.0)	n.d.	**3 a**	43:57

Therefore, we switched to the tiglic esters **4 a**–**c** (R’, R’’=H) which give rise to the substitution pattern we were looking for. To our big surprise, with these ester enolates the linear γ‐products **5 a**–**c** were formed almost exclusively (Table 2, entries 1–3). The yields were almost the same as in the previous example, only in case of the methyl ester **5 c** the yield was a little lower (entry 3). In all examples only the formation of the (*E*)‐isomer was observed. To investigate the scope and limitations of this protocol also substituted tiglic esters **4 d**–**f** were evaluated. Introduction of an additional methyl group on the double bond (**4 d**) was well accepted and the tetra substituted double bond in **5 d** was obtained in good (*E*/*Z*)‐ratio of 94:6 (entry 4). Addition of another methyl group at the γ‐position (**4 e**) had no influence on the yield but on the regioselectivity (entries 5 and 6). The γ/α‐selectivity of **5 e** was significantly lower, compared to the examples without substituent on the γ‐position, but was still in a synthetically useful range. While the α‐product was an inseparable mixture of diastereomers and (*E*/*Z*)‐isomers, the γ‐product was obtained with good diastereoselectivity, especially in case of the ethyl ester **5 e** (entry 5). The product mixtures were analyzed after oxidation, the corresponding alcohols could be separated by flash chromatography. It should be mentioned that the yields given in tables 1 and 2 are isolated yields after chromatography.

**Table 2:** Matteson homologations of **2** using tiglic ester dienolates.

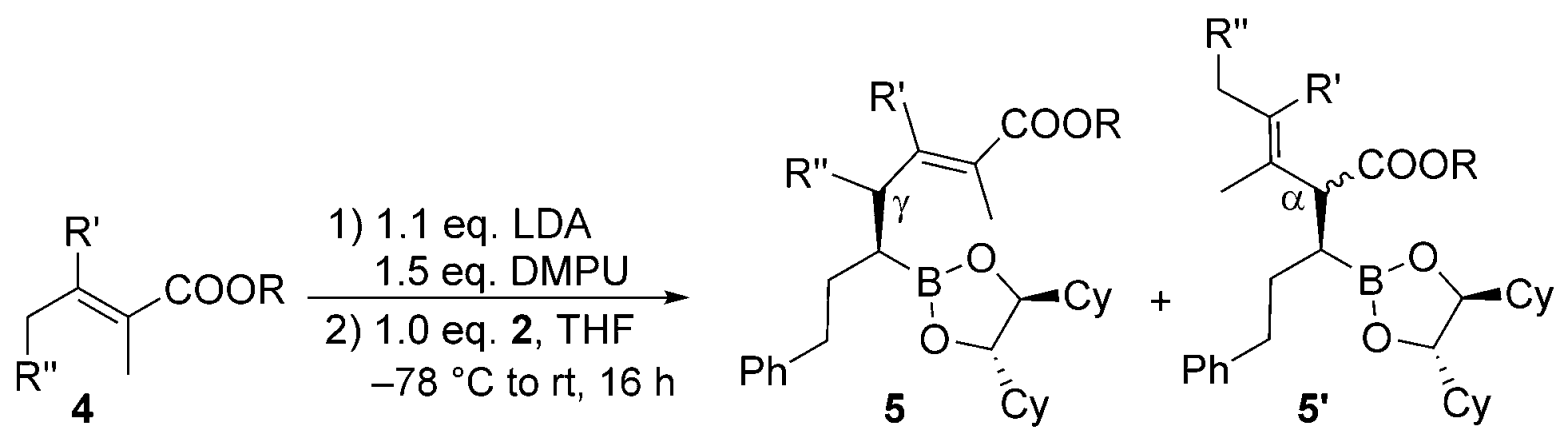							
Entry	4	R	R’	R’’	Product	Yield [%]	Ratio **5**:**5**’
1	**4 a**	*t*‐Bu	H	H	**5 a**	63	96:4
2	**4 b**	Et	H	H	**5 b**	60	>96:4
3	**4 c**	Me	H	H	**5 c**	51	>96:4
4	**4 d**	Et	Me	H	**5 d**	52	>96:4
5	**4 e**	Et	H	Me	**5 e**	62	78^[a]^:22
6	**4 f**	*t*‐Bu	H	Me	**5 f**	60	85^[b]^:15

[a] Diastereomeric ratio 4*S*:4*R*:95:5. [b] Diastereomeric ratio 4*S*:4*R*: 83:17.

All boronic esters **5** were oxidized to the corresponding unsaturated δ‐hydroxyesters. Depending on the oxidation protocol different products become available. Using “typical” oxidation conditions methyl ester **5 c** was also hydrolyzed to the free carboxylic acid **6 c** (Scheme 2). In contrast, under the same conditions *tert*‐butyl esters (**5 a**) are not affected. Using Na_2_CO_3_ as a milder base also the ethyl ester of **5 b** remains untouched. In the last two cases the hydroxy esters **6 a** and **6 b** could not be separated from the chiral diol used as auxiliary in the boronic ester. But this problem can be solved by adding commercially available methyl boronic acid forming the chiral methyl boronic ester **B** (Scheme 1), which can be used again in Matteson homologations. In principle, the original boronic esters used in the homologation sequence can be „recovered“, what makes this protocol rather economic. All alcohols **6** were obtained with an *ee*>90 % clearly indicating that no significant epimerization in the α‐bromo boronic ester occurred.

**Scheme 2:**
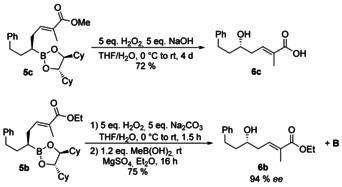
Oxidation of chiral boronic esters **5**.

Finally we reacted tiglic ester **4 b** with a series of different boronic esters (see Supporting Information) to illustrate the scope and applicability of this protocol (Figure 2). Linear as well as branched and functionalized boronic esters can be used giving yields in the range of 61–78 %, independent of the substitution pattern and the chiral auxiliary used. Only one set of signals are observed in the NMR spectra of the purified products **7**, indicating a high diastereoselectivity.

**Figure 2:**
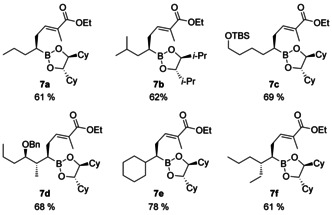
Chiral boronic esters obtained by Matteson dienolate homologation.

As a proof of concept we also synthesized a protected polyketide fragment of *epi*‐lagunamide A starting from known boronic ester **8**. Homologation of **8** with CHBr_2_Li followed by reaction with the dienolate of ethyl tiglate provided **9** in high yield, which was then oxidized to the desired polyketide fragment **10**. Moreover, we recovered the chiral auxiliary DICHED after oxidation (Scheme 3).

**Scheme 3:**
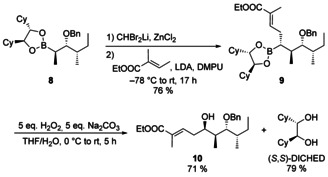
Synthesis of the polyketide fragment **11** of *epi*‐lagunamide A.

In conclusion, we could show that ester dienolates are good nucleophiles for Matteson homologations. Especially enolates of tiglic esters give excellent regio‐ and stereoselectivities, and in combination with a subsequent oxidation of the prolonged boronic ester formed this approach allows the straightforward synthesis of polyketide structures in only a few steps. Further synthetic applications are currently under investigation.

## Conflict of interest

The authors declare no conflict of interest.

## Supporting information

As a service to our authors and readers, this journal provides supporting information supplied by the authors. Such materials are peer reviewed and may be re‐organized for online delivery, but are not copy‐edited or typeset. Technical support issues arising from supporting information (other than missing files) should be addressed to the authors.

Supplementary
