# Supplementary material for: A Straightforward Synthesis of Polyketides via Ester Dienolate Matteson Homologation
Source: Chemistry. 2020 Dec 15;27(3):949–53. doi: 10.1002/chem.202004650 (PMC7839490; doi:10.1002/chem.202004650)

Chemistry–A European Journal

Supporting Information

A Straightforward Synthesis of Polyketides via Ester Dienolate Matteson Homologation

Oliver Andler and Uli Kazmaier*[a]

Experimental Section	S2
General remarks	S2
General procedures	S2
Preparation of α-bromo boronic ester **2**	S4
Alkylation of dienolates with α-bromo boronic ester **2**	S4
Oxidation of boronic esters **5a–f**	S10
Preparation of boronic esters **7a–f**	S15
Dienolate Matteson homologation of **7a–f** with ethyl tiglate	S19
Synthesis of the polyketide unit of *epi*-Lagunamide A	S23
Copies of the NMR Spectra and HPLC-chromatograms	S25

Experimental Section

General remarks: All air- or moisture-sensitive reactions were carried out in oven-dried glassware (75 °C) under an atmosphere of nitrogen. Dried solvents were distilled before use: THF was distilled from sodium/benzophenone, diisopropylamine was dried with CaH_2 before distillation. Anhydrous DCM and DMSO were purchased from Acros Organics and anhydrous DMPU from Sigma Aldrich and stored under nitrogen. Petroleum ether (40-60 °C) and ethyl acetate were distilled prior to use. Zinc chloride was fused in vacuo (0.1 mbar) prior to use. The products were purified by flash chromatography on silica gel columns (Macherey-Nagel 60, 0.063-0.2 mm or 0.04-0.063 mm). For reversed-phase flash chromatography, a Büchi Reveleris® Prep Chromatography System and Kinesis GmbH Telos® Flash C18 columns were used. Preparative HPLC was performed on a Büchi Reveleris® Prep Chromatography System using a Phenomenex Luna® C18(2) 100 Å column (250 x 21.1 mm, 5 µm). Analytical TLC was performed on pre-coated silica gel plates (Macherey-Nagel, Polygram® SIL G/UV_{254}). Visualization was accomplished with UV-light, $KMnO_4$ solution or cerium(IV)/ammonium molybdate solution. Melting points were determined with a MEL-TEMP II apparatus and are uncorrected. ^1H and ^{13}C NMR spectra were recorded with Bruker AV 400 Ultra Shield [400 MHz (^1H) and 100 MHz (^{13}C)] or Bruker AV 500 [500 MHz (^1H) and 125 MHz (^{13}C)] spectrometers in $CDCl_3$ or acetone-d_6. Chemical shifts are reported in ppm (δ) with respect to TMS, and $CHCl_3$ or acetone-d_5 was used as the internal standard. Selected signals for the minor diastereomers are extracted from the spectra of the diastereomeric mixture. Enantiomeric ratios were determined by HPLC [column: Daicel Chiralcel OD-H (250 x 4.6 mm, 5 µm)]. Optical rotations were measured with a PerkinElmer 341 polarimeter at the sodium D line (589 nm). Mass spectra were recorded with a Finnigan MAT 95 spectrometer (CI).

General procedures

General procedure for the preparation of α-bromo boronic esters (GP 1).

LDA solution: *n*-butyllithium (1.6 M in hexanes, 1.25 eq.) was added dropwise to a solution of diisopropylamine (1.35 eq.) in anhydrous THF (0.2 ml/mmol) at –40 °C. The mixture was allowed to warm to room temperature and stirred for 20 min.

Homologation: The freshly prepared LDA solution was slowly added to a solution of the boronic ester (1.0 eq.) and dibromomethane (3.0 eq.) in anhydrous THF (1.4 ml/mmol) at –78 °C. After stirring at this temperature for 1 h, a solution of zinc chloride (2.0–3.0 eq.) in anhydrous THF (0.5 ml/mmol $ZnCl_2$) was added dropwise and the mixture was allowed to slowly warm to room temperature overnight. Pentane was added and the reaction was quenched by the addition of saturated NH_4Cl solution. After stirring for 5 min, the layers were separated and the aqueous layer was extracted twice with pentane. The combined organic layers were dried over Na_2SO_4 and concentrated in vacuo to yield the crude α-bromo boronic ester which was used in the next step without further purification.

Note: As α-bromo boronic esters rapidly epimerize upon storage,[1] they should always be freshly prepared and directly used in the next step.

[1] D. S. Matteson, H.-W. Man, *J. Org. Chem.* **1994**, *59*, 5734–5741.

General procedure for the alkylation of dienolates with α-bromo boronic esters (GP 2).

n-butyllithium (1.6 M in hexanes, 1.10 eq.) was added dropwise to a solution of diisopropylamine (1.15 eq.) in anhydrous THF (2.5 ml/mmol) at –40 °C. The mixture was allowed to warm to room temperature and stirred for 20 min. After cooling the LDA solution to –78 °C, anhydrous DMPU (1.5 eq.) was added and the mixture was stirred for 30 min followed by dropwise addition of the α,β-unsaturated ester. The yellow dienolate solution was stirred for another 30 min at –78 °C and a solution of the freshly prepared α-bromo boronic ester in anhydrous THF (2.5 ml/mmol) was added dropwise. After completion of the addition, the mixture was allowed to slowly warm to room temperature overnight. The reaction was quenched by the addition of saturated NH_4Cl solution. After stirring for 5 min, the layers were separated and the aqueous layer was extracted twice with pentane. The combined organic layers were dried over Na_2SO_4, concentrated in vacuo and the crude product was purified by flash chromatography (petroleum ether, ethyl acetate).

General procedure for the oxidation of boronic esters to alcohols (GP 3).

To a solution of the boronic ester (1.0 eq.) in THF (2 ml/mmol) were added hydrogen peroxide (33 % in water, 5.0 eq.) and a solution of sodium hydroxide or sodium carbonate (5.0 eq.) in water (2 ml/mmol). The mixture was allowed to warm to room temperature and stirred until TLC control indicated full conversion. Brine was added and the aqueous layer was extracted three times with diethyl ether. The combined organic layers were dried over Na_2SO_4, concentrated in vacuo and purified by flash chromatography. If necessary, the product was further purified by preparative HPLC.

If the desired alcohol and the chiral auxiliary (*S*,*S*)-DICHED were not separable via chromatography, (*S*,*S*)-DICHED was converted to its methylboronic ester as follows: The crude product was dissolved in diethyl ether (5 ml/mmol) and methylboronic acid (1.2 eq.) and an excess of $MgSO_4$ were added at room temperature. After stirring at room temperature overnight, the mixture was filtered, concentrated in vacuo and purified by flash chromatography (petroleum ether, ethyl acetate).

General procedure for Matteson homologations with dichloromethyllithium (GP 4).

LDA solution: *n*-butyllithium (1.6 M in hexanes, 1.25 eq.) was added dropwise to a solution of diisopropylamine (1.35 eq.) in anhydrous THF (0.2 ml/mmol) at –40 °C. The mixture was allowed to warm to room temperature and stirred for 20 min.

Homologation: The freshly prepared LDA solution was slowly added to a solution of the boronic ester (1.0 eq.) and anhydrous dichloromethane (3.0 eq.) in anhydrous THF (1.4 ml/mmol) at –40 °C. After stirring at this temperature for 10 min, a solution of zinc chloride (2.0–4.0 eq.) in anhydrous THF (0.5 ml/mmol $ZnCl_2$) was added and the mixture was stirred for 2 h at room temperature.

Reaction with nucleophile: The mixture was cooled to 0 °C and the nucleophile solution was slowly added. After stirring for 1–3 d at room temperature, saturated NH_4Cl was added. The biphasic mixture was stirred for 5 min, then the layers were separated and the aqueous layer was extracted twice with pentane. The combined organic layers were dried over Na_2SO_4 and concentrated in vacuo. If necessary, the crude product was purified by flash chromatography.

(4*S*,5*S*)-2-[(*R*)-1-Bromo-3-phenylpropyl]-4,5-dicyclohexyl-1,3,2-dioxaborolane (2)

(4*S*,5*S*)-4,5-Dicyclohexyl-2-phenethyl-1,3,2-dioxaborolane **1** was prepared as described by Molander *et al.*[2]

According to GP 1 305 mg (896 µmol) boronic ester **1**, 188 µl (ρ = 2.49 g/ml, 2.69 mmol) dibromomethane, 700 µl (1.6 M in hexanes, 1.12 mmol) *n*-butyllithium, 172 µl (ρ = 0.71 g/ml, 1.21 mmol) diisopropylamine and 244 mg (1.79 mmol) zinc chloride were reacted to give crude **2** in 98 % yield (381 mg, 879 µmol) as a pale yellow oil, R_f = 0.28 (petroleum ether, ethyl acetate 98:2); $[\alpha]_D^{20}$ = –62.3 (c =1.0, $CHCl_3$).

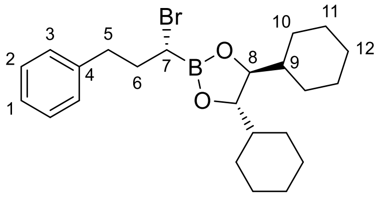

^{1}H-NMR (400 MHz, $CDCl_3$): δ = 0.99 (m, 2 H, 11-H), 1.07 (m, 2 H, 10-H), 1.13–1.30 (m, 6 H, 10-H', 11-H', 12-H), 1.37 (m, 2 H, 9-H), 1.62 (m, 2 H, 10-H"), 1.69 (m, 2 H, 12-H'), 1.72–1.84 (m, 6 H, 10-H"', 11-H"), 2.19 (m, 1 H, 6-H_a), 2.23 (m, 1 H, 6-H_b), 2.75 (m, 1 H, 5-H_a), 2.85 (m, 1 H, 5-H_b), 3.36 (dd, $^3J_{7,6a}$ = 8.1 Hz, $^3J_{7,6b}$ = 7.1 Hz, 1 H, 7-H), 7.17–7.25 (m, 3 H, 1-H, 3-H), 7.29 (m, 2 H, 2-H).
^{13}C-NMR (100 MHz, $CDCl_3$): δ = 25.8 (t, C-11), 26.0 (t, C-10), 26.4 (t, C-12), 27.3 (t, C-11'), 28.2 (t, C-10'), 32.6 (bs, C-7), 34.6 (t, C-5), 35.8 (t, C-6), 42.9 (d, C-9), 84.0 (d, C-8), 126.0 (d, C-1), 128.4 (d, C-2), 128.6 (d, C-3), 141.0 (s, C-4).
HRMS (CI) calcd for $C_{23}H_{34}O_2BBr$ $[M]^+$: 432.1830, found: 432.1829.

***tert*-Butyl (2*E*,5*S*)-5-[(4*S*,5*S*)-4,5-dicyclohexyl-1,3,2-dioxaborolan-2-yl]-7-phenylhept-2-enoate (3a)**

382 mg (882 µmol) α-bromo boronic ester **2**, 157 mg (1.10 mmol) *tert*-butyl crotonate, 144 µl (ρ = 0.71 g/ml, 1.01 mmol) diisopropylamine, 606 µl (1.6 M in hexanes, 970 µmol) *n*-butyllithium and 159 µl (ρ = 1.06 g/ml, 1.32 mmol) DMPU were reacted according to GP 2. Purification of the crude produt by flash chromatography (petroleum ether, ethyl acetate 95:5) gave **3a** as a mixture of isomers (γ:α = 57:43 according to ^{1}H NMR) in 65 % yield (284 mg, 553 µmol) as a colorless oil, R_f = 0.22 (petroleum ether, ethyl acetate 95:5).

major isomer (γ isomer):

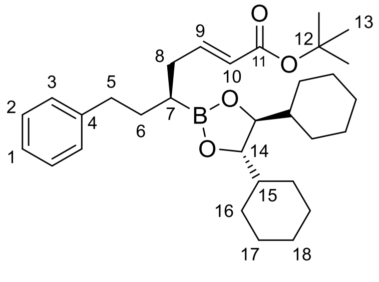

^{1}H-NMR (400 MHz, $CDCl_3$): δ = 0.92–1.38 (m, 13 H, 7-H, 15-H, 16-H, 17-H), 1.47 (s, 9 H, 13-H), 1.60 (m, 2 H, 16-H’), 1.64–1.72 (m, 3 H, 6-H_a, 18-H), 1.71–1.87 (m, 7 H, 6-H_b, 16-H’’, 17-H’, 18-H’), 2.34 (m, 2 H, 8-H), 2.66 (m, 2 H, 5-H), 3.86 (m, 2 H, 15-H), 5.74 (d, $^3J_{10,9}$ = 15.7 Hz, 1 H, 10-H), 6.86 (dt, $^3J_{9,10}$ = 15.5 Hz, $^3J_{9,8}$ = 7.2 Hz, 1 H, 9-H), 7.11–7.21 (m, 3 H, 1-H, 3-H), 7.26 (m, 2 H, 2-H).
^{13}C-NMR (100 MHz, $CDCl_3$): δ = 25.9 (t, C-17), 26.0 (t, C-16), 26.4 (t, C-18), 27.5 (t, C-17’), 28.2 (q, C-13), 28.5 (t, C-16’), 33.1 (t, C-6), 33.7 (t, C-8), 35.4 (t, C-5), 43.1 (d,

[2] G. A. Molander, S. R. Wisniewski, *J. Am. Chem. Soc.* **2012**, *134*, 16856–16868.

C-15), 79.8 (s, C-12), 83.5 (d, C-14), 123.5 (d, C-10), 125.7 (d, C-1), 128.3 (d, C-2), 128.4 (d, C-3), 142.7 (s, C-4), 147.7 (d, C-9), 166.0 (s, C-12).
The signal of C-7 was not detected.
minor isomer (α isomer, selected signals):

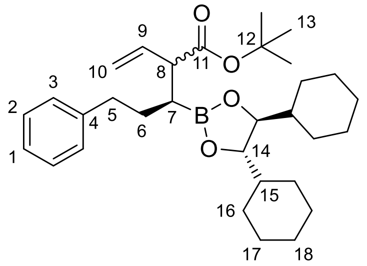

^{1}H-NMR (400 MHz, $CDCl_3$): δ = 5.14 (m, 2 H, 10-H), 5.79 (m, 1 H, 9-H).
HRMS (CI) calcd for $C_{31}H_{48}O_4B$ $[M+H]^+$: 495.3640, found: 495.3606.

tert-Butyl (*S*,*E*)-5-hydroxy-7-phenylhept-2-enoate

116 mg (235 µmol) boronic ester **3a** (mixture of isomers), 109 µl (33 % in water, ρ = 1.11 g/ml, 1.17 mmol) hydrogen peroxide and 47 mg (1.17 mmol) sodium hydroxide were reacted according to GP 3. After 1 h, the reaction was worked up and the crude product was treated with 17 mg (281 µmol) methylboronic acid to separate (*S*,*S*)-DICHED. Purification by flash chromatography (petroleum ether, ethyl acetate 95:5, 8:2) gave the title compound (single isomer) in 36 % yield (23 mg, 83 µmol) as a colorless oil, R_f = 0.29 (petroleum ether, ethyl acetate 8:2); $[\alpha]_D^{20}$ = –10.0 (c =1.0, $CHCl_3$).

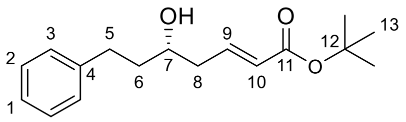

^{1}H-NMR (400 MHz, $CDCl_3$): δ = 1.48 (s, 9 H, 13-H), 1.72 (bs, 1 H, OH), 1.80 (m, 2 H, 6-H), 2.37 (m, 2 H, 8-H), 2.69 (dt, $^2J_{5a,5b}$ = 14.1 Hz, $^3J_{5a,6}$ = 8.2 Hz, 1 H, 5-H_a), 2.81 (dt, $^2J_{5b,5a}$ = 13.9 Hz, $^3J_{5b,6}$ = 7.6 Hz, 1 H, 5-H_b), 3.77 (m, 1 H, 7-H), 5.83 (d, $^3J_{10,9}$ = 15.5 Hz, 1 H, 10-H), 6.85 (dt, $^3J_{9,10}$ = 15.4 Hz, $^3J_{9,8}$ = 7.5 Hz, 1 H, 9-H), 7.14–7.23 (m, 3 H, 1-H, 3-H), 7.29 (m, 2 H, 2-H).
^{13}C-NMR (100 MHz, $CDCl_3$): δ = 28.1 (q, C-13), 31.9 (t, C-5), 38.6 (t, C-6), 40.2 (t, C-8), 69.7 (d, C-7), 80.3 (s, C-12), 125.8 (d, C-10), 125.9 (d, C-1), 128.4 (d, C-3), 128.4 (d, C-2), 141.6 (s, C-4), 143.5 (d, C-9), 165.6 (s, C-11).
HRMS (CI) calcd for $C_{17}H_{23}O_2$ $[M+H–H_2O]^+$: 259.1693, found: 259.1690.

Ethyl (2*E*,5*S*)-5-[(4*S*,5*S*)-4,5-dicyclohexyl-1,3,2-dioxaborolan-2-yl]-7-phenylhept-2-enoate (3b)

127 mg (293 µmol) α-bromo boronic ester **2**, 46 µl (ρ = 0.918 g/ml, 366 µmol) ethyl crotonate, 48 µl (ρ = 0.71 g/ml, 337 µmol) diisopropylamine, 202 µl (1.6 M in hexanes, 322 µmol) *n*-butyllithium and 53 µl (ρ = 1.06 g/ml, 440 µmol) DMPU were reacted according to GP 2. Purification of the crude produt by flash chromatography (petroleum ether, ethyl acetate 95:5) gave **3b** as a mixture of isomers (γ:α = 63:37 according to ^{1}H NMR) in 54 % yield (74 mg, 159 µmol) as a colorless oil, R_f = 0.20 (petroleum ether, ethyl acetate 95:5).

major isomer (γ isomer):

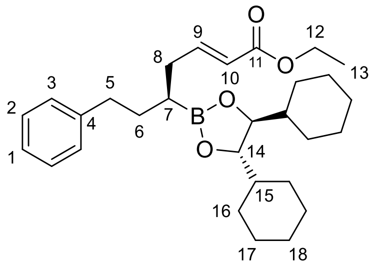

^{1}H-NMR (400 MHz, $CDCl_3$): δ = 0.99 (m, 2 H, 17-H), 1.07 (m, 2 H, 16-H), 1.12–1.41 (m, $^3J_{13,12}$ = 7.1 Hz, 12 H, 7-H, 13-H, 15-H, 16-H', 17-H'), 1.61 (m, 2 H, 16-H''), 1.64–1.72 (m, 3 H, 6-H_a, 18-H), 1.72–1.89 (m, 7 H, 6-H_b, 16-H''', 17-H'', 18-H'), 2.35 (m, 2 H, 8-H), 2.64 (m, 2 H, 5-H), 3.86 (m, 2 H, 15-H), 4.17 (q, $^3J_{12,13}$ = 7.1 Hz, 2 H, 12-H), 5.82 (d, $^3J_{10,9}$ = 15.5 Hz, 1 H, 10-H), 6.97 (dt, $^3J_{9,10}$ = 15.4 Hz, $^3J_{9,8}$ = 7.3 Hz, 1 H, 9-H), 7.13–7.22 (m, 3 H, 1-H, 3-H), 7.26 (m, 2 H, 2-H).
^{13}C-NMR (100 MHz, $CDCl_3$): δ = 14.3 (q, C-14), 25.9 (t, C-17), 26.0 (t, C-16), 26.4 (t, C-18), 27.5 (t, C-17'), 28.5 (t, C-16'), 33.2 (t, C-6), 33.9 (t, C-8), 35.4 (t, C-5), 43.1 (d, C-15), 60.1 (t, C-12), 83.6 (d, C-14), 121.8 (d, C-10), 125.7 (d, C-1), 128.3 (d, C-2), 128.4 (d, C-3), 142.6 (s, C-4), 149.0 (d, C-9), 166.6 (s, C-11).
The signal of C-7 was not detected.
minor isomer (α isomer, selected signals):

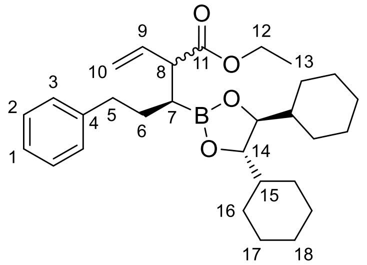

^{1}H-NMR (400 MHz, $CDCl_3$): δ = 5.14 (m, 2 H, 10-H), 5.88 (m, 1 H, 9-H).
HRMS (CI) calcd for $C_{29}H_{44}O_4B$ $[M+H]^+$: 467.3327, found: 467.3327.

tert-Butyl tiglate (4a)

34.4 ml *n*-butyllithium (1.6 M in hexanes, 55.0 mmol, 1.0 eq.) were added dropwise to a solution of 5.26 ml (ρ = 0.775 g/ml, 55.0 mmol, 1.0 eq.) *tert*-butanol in 65 ml anhydrous THF at room temperature. After stirring for 40 min, a solution of 6.52 g (55.0 mmol, 1.0 eq.) tigloyl chloride in 45 ml anhydrous THF was added over 10 min. The mixture was heated to reflux for 1 h. After cooling to room temperature, water was added and the aqueous layer was extracted 3x with diethyl ether. The combined organic layers were dried over Na_2SO_4 and concentrated in vacuo (40 °C, > 300 mbar). Distillation of the residue (90–95 °C, 105 mbar) gave **4a** in 82 % yield (7.04 g, 45.1 mmol) as a colorless liquid.

NMR spectra of **4a** were in accordance with previously published data.[3]

The ester **4b** – **4e** are commercially available or were prepared according to the literature.

[3] M. S. Baird, H. L. Fitton, W. Clegg, A. McCamley, *J. Chem. Soc., Perkin Trans. 1* **1993**, 321–326.

tert-Butyl (*E*)-2-methylpent-2-enoate (4f)

(*E*)-2-Methylpent-2-enoyl chloride was prepared as described previously.[4]

7.17 ml *n*-Butyllithium (1.6 M in hexanes, 11.5 mmol, 1.0 eq.) were added dropwise to a solution of 1.10 ml *tert*-butanol (ρ = 0.775 g/ml, 11.5 mmol, 1.0 eq.) in 15 ml anhydrous THF at room temperature. After stirring for 40 min, a solution of 1.52 g (55.0 mmol, 1.0 eq.) (*E*)-2-methylpent-2-enoyl chloride in 10 ml anhydrous THF was added over 10 min. The mixture was heated to reflux for 1 h. After cooling to room temperature, water was added and the aqueous layer was extracted 3x with diethyl ether. The combined organic layers were dried over Na_2SO_4 and concentrated in vacuo (40 °C, > 100 mbar). Purification of the residue by flash chromatography (pentane, diethyl ether 96:4) gave **4f** in 92 % yield (1.80 g, 10.6 mmol) as a colorless liquid, R_f = 0.34 (pentane, diethyl ether 96:4).

NMR spectra of **4f** were in accordance with previously published data.[5]

tert-Butyl (2*E*,5*S*)-5-[(4*S*,5*S*)-4,5-dicyclohexyl-1,3,2-dioxaborolan-2-yl]-2-methyl-7-phenylhept-2-enoate (5a)

382 mg (882 µmol) α-bromo boronic ester **2**, 172 mg (1.10 mmol) **4a**, 144 µl (ρ = 0.71 g/ml, 1.01 mmol) diisopropylamine, 606 µl (1.6 M in hexanes, 970 µmol) *n*-butyllithium and 159 µl (ρ = 1.06 g/ml, 1.32 mmol) DMPU were reacted according to GP 2. Purification of the crude produt by flash chromatography (petroleum ether, ethyl acetate 95:5) gave **5a** as a mixture of isomers (γ:α = 96:4 according to ^{1}H NMR) in 63 % yield (281 mg, 553 µmol) as a colorless oil, R_f = 0.26 (petroleum ether, ethyl acetate 95:5); $[\alpha]_D^{20}$ = –12.1 (c =1.0, $CHCl_3$).

major isomer (γ isomer):

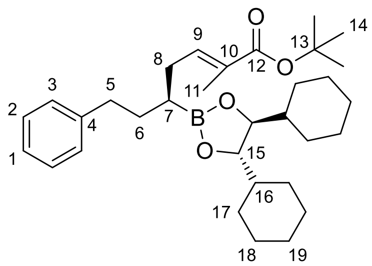

^{1}H-NMR (400 MHz, $CDCl_3$): δ = 0.98 (m, 2 H, 18-H), 1.08 (m, 2 H, 17-H), 1.12–1.37 (m, 9 H, 7- H 16-H, 17-H', 18-H'), 1.48 (s, 9 H, 14-H), 1.60 (m, 2 H, 17-H''), 1.64–1.72 (m, 3 H, 6-H_a, 19-H), 1.72–1.85 (m, 10 H, 6-H_b, 11-H, 17-H''', 18-H'', 19-H'), 2.26 (ddd, $^2J_{8a,8b}$ = 14.9 Hz, $^3J_{8a,7}$ ≈ $^3J_{8a,9}$ = 7.4 Hz, 1 H, 8-H_a), 2.33 (ddd, $^2J_{8b,8a}$ = 14.9 Hz, $^3J_{8b,7}$ ≈ $^3J_{8b,9}$ = 7.4 Hz, 1 H, 8-H_b), 2.64 (m, 2 H, 5-H), 3.86 (m, 2 H, 15-H), 6.67 (tq, $^3J_{9,8}$ = 7.5 Hz, $^4J_{9,11}$ = 1.3 Hz, 1 H, 9-H), 7.12–7.21 (m, 3 H, 1-H, 3-H), 7.26 (m, 2 H, 2-H).
^{13}C-NMR (100 MHz, $CDCl_3$): δ = 12.5 (q, C-11), 22.7 (bs, C-7), 25.9 (t, C-18), 26.0 (t, C-17), 26.4 (t, C-19), 27.4 (t, C-18'), 28.1 (q, C-14), 28.4 (t, C-17'), 30.4 (t, C-8), 33.3 (t, C-6), 35.5 (t, C-5), 43.1 (d, C-16), 79.7 (s, C-13), 83.5 (d, C-15), 125.6 (d, C-1), 128.3 (d, C-2), 128.4 (d, C-3), 129.2 (s, C-10), 141.0 (d, C-9), 142.8 (s, C-4), 167.5 (s, C-12).

[4] R. Huston, M. Rey, A. S. Drieding, *Helv. Chim. Acta* **1982**, *63*, 1563–1575.
[5] S. G. Davies, O. Ichihara, I. A. S. Walters, *J. Chem. Soc., Perkin Trans. 1* **1994**, 1141–1147.

minor isomer (α isomer, selected signals):

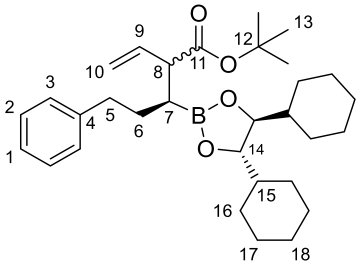

^{1}H-NMR (400 MHz, $CDCl_3$): δ = 1.49 (s, 9 H, 13-H), 5.09 (m, 2 H, 10-H), 5.91 (m, 1 H, 9-H).
HRMS (CI) calcd for $C_{32}H_{50}O_4B$ $[M+H]^+$: 509.3797, found: 509.3801.

Ethyl (2*E*,5*S*)-5-[(4*S*,5*S*)-4,5-dicyclohexyl-1,3,2-dioxaborolan-2-yl]-2-methyl-7-phenyl-hept-2-enoate (5b)

127 mg (293 µmol) α-bromo boronic ester **2**, 51 µl (ρ = 0.923 g/ml, 366 µmol) ethyl tiglate **4b**, 48 µl (ρ = 0.71 g/ml, 337 µmol) diisopropylamine, 202 µl (1.6 M in hexanes, 322 µmol) *n*-butyllithium and 53 µl (ρ = 1.06 g/ml, 440 µmol) DMPU were reacted according to GP 2. Purification of the crude produt by flash chromatography (petroleum ether, ethyl acetate 95:5) gave **5b** in 60 % yield (85 mg, 177 µmol) as a colorless oil, R_f = 0.22 (petroleum ether, ethyl acetate 95:5); $[\alpha]_D^{20}$ = –17.8 (c =1.0, $CHCl_3$).

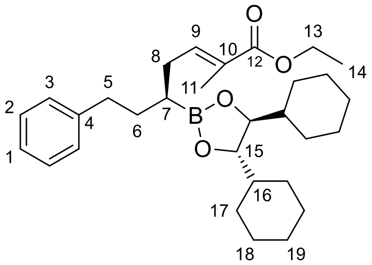

^{1}H-NMR (400 MHz, $CDCl_3$): δ = 0.98 (m, 2 H, 18-H), 1.07 (m, 2 H, 17-H), 1.14–1.39 (m, $^3J_{14,13}$ = 7.1 Hz, 12 H, 7-H, 14-H, 16-H, 17-H’, 18-H’), 1.60 (m, 2 H, 17-H’’), 1.64–1.72 (m, 3 H, 6-H_a, 19-H), 1.72–1.82 (m, 7 H, 6-H_b, 17-H’’’, 18-H’’, 19-H’), 1.83 (s, 3 H, 11-H), 2.32 (m, 2 H, 8-H), 2.64 (m, 2 H, 5-H), 3.86 (m, 2 H, 15-H), 4.17 (q, $^3J_{13,14}$ = 7.1 Hz, 2 H, 13-H), 6.78 (tq, $^3J_{9,8}$ = 7.5 Hz, $^4J_{9,11}$ = 1.3 Hz, 1 H, 9-H), 7.13–7.22 (m, 3 H, 1-H, 3-H), 7.26 (m, 2 H, 2-H).
^{13}C-NMR (100 MHz, $CDCl_3$): δ = 12.5 (q, C-11), 14.3 (q, C-14), 22.8 (bs, C-7), 25.9 (t, C-18), 26.0 (t, C-17), 26.4 (t, C-19), 27.4 (t, C-18’), 28.5 (t, C-17’), 30.4 (t, C-8), 33.4 (t, C-6), 35.5 (t, C-5), 43.1 (d, C-16), 60.3 (t, C-13), 83.5 (d, C-15), 125.7 (d, C-1), 127.9 (s, C-10) 128.3 (d, C-2), 128.4 (d, C-3), 142.1 (d, C-9), 142.7 (s, C-4), 168.2 (s, C-12).
The α-Isomer could not be detected.
HRMS (CI) calcd for $C_{30}H_{45}O_4B$ $[M]^+$: 480.3405, found: 480.3396.

Methyl (2*E*,5*S*)-5-[(4*S*,5*S*)-4,5-dicyclohexyl-1,3,2-dioxaborolan-2-yl]-2-methyl-7-phenyl-hept-2-enoate (5c)

127 mg (293 µmol) α-bromo boronic ester **2**, 44 µl (ρ = 0.95 g/ml, 366 µmol) methyl tiglate **4c**, 48 µl (ρ = 0.71 g/ml, 337 µmol) diisopropylamine, 202 µl (1.6 M in hexanes, 322 µmol) *n*-butyllithium and 53 µl (ρ = 1.06 g/ml, 440 µmol) DMPU were reacted according to GP 2. Purification of the crude produt by flash chromatography (petroleum ether, ethyl acetate 95:5)

gave **5c** (γ:α > 96:4) in 51 % yield (70 mg, 177 µmol) as a colorless oil, R_f = 0.15 (petroleum ether, ethyl acetate 95:5); $[\alpha]_D^{20}$ = –31.7 (c =1.0, $CHCl_3$).

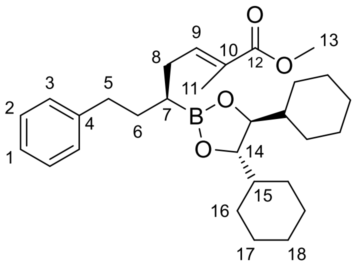

^{1}H-NMR (400 MHz, $CDCl_3$): δ = 0.98 (m, 2 H, 17-H), 1.07 (m, 2 H, 16-H), 1.14–1.39 (m, $^3J_{14,13}$ = 7.1 Hz, 9 H, 7-H, 15-H, 16-H', 17-H'), 1.61 (m, 2 H, 16-H''), 1.64–1.72 (m, 3 H, 6-H_a, 18-H), 1.72–1.82 (m, 7 H, 6-H_b, 16-H''', 17-H'', 18-H'), 1.84 (s, 3 H, 11-H), 2.32 (m, 2 H, 8-H), 2.65 (m, 2 H, 5-H), 3.72 (s, 3 H, 13-H), 3.86 (m, 2 H, 15-H), 6.79 (tq, $^3J_{9,8}$ = 7.5 Hz, $^4J_{9,11}$ = 1.2 Hz, 1 H, 9-H), 7.12–7.22 (m, 3 H, 1-H, 3-H), 7.27 (m, 2 H, 2-H).
^{13}C-NMR (100 MHz, $CDCl_3$): δ = 12.5 (q, C-11), 22.8 (bs, C-7), 25.9 (t, C-17), 26.0 (t, C-16), 26.4 (t, C-18), 27.4 (t, C-17'), 28.4 (t, C-16'), 30.4 (t, C-8), 33.4 (t, C-6), 35.5 (t, C-5), 43.1 (d, C-15), 51.6 (q, C-13), 83.5 (d, C-14), 125.7 (d, C-1), 127.6 (s, C-10) 128.3 (d, C-2), 128.4 (d, C-3), 142.4 (d, C-9), 142.7 (s, C-4), 168.6 (s, C-12).
The α-Isomer could not be detected.
HRMS (CI) calcd for $C_{29}H_{44}O_4B$ $[M+H]^+$: 467.3327, found: 467.3327.

Ethyl (2*E*,5*S*)-5-[(4*S*,5*S*)-4,5-dicyclohexyl-1,3,2-dioxaborolan-2-yl]-2,3-dimethyl-7-phenylhept-2-enoate (5d)

Ethyl 2,3-dimetylbut-2-enoate **4d** was prepared as described by Baiker *et al.* [6]
171 mg (395 µmol) α-bromo boronic ester **2**, 78 mg (494 µmol) **4d**, 65 µl (ρ = 0.71 g/ml, 454 µmol) diisopropylamine, 272 µl (1.6 M in hexanes, 434 µmol) *n*-butyllithium and 53 µl (ρ = 1.06 g/ml, 592 µmol) DMPU were reacted according to GP 2. Purification of the crude produt by flash chromatography (petroleum ether, ethyl acetate 96:4) gave **5d** (*E*:*Z* = 94:6 according to ^{1}H-NMR) in 52 % yield (102 mg, 206 µmol) as a colorless oil, R_f = 0.27 (petroleum ether, ethyl acetate 95:5); $[\alpha]_D^{20}$ = –35.8 (c =1.0, $CHCl_3$).

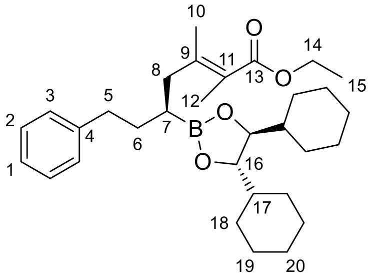

major isomer (E isomer):

^{1}H-NMR (400 MHz, $CDCl_3$): δ = 0.90–1.12 (m, 4 H, 18-H, 19-H), 1.15–1.44 (m, $^3J_{15,14}$ = 7.1 Hz, 12 H, 7-H, 15-H, 17-H, 18-H', 19-H'), 1.60 (m, 2 H, 17-H''), 1.64–1.72 (m, 3 H, 6-H_a, 20-H), 1.72–1.85 (m, 7 H, 6-H_b, 18-H''', 19-H'', 20-H'), 1.87 (m, 3 H, 12-H), 1.94 (m, 3 H, 10-H), 2.25 (dd, $^2J_{8a,8b}$ = 13.1 Hz, $^3J_{8a,7}$ = 8.1 Hz, 1 H, 8-H_a), 2.32 (dd, $^2J_{8b,8a}$ = 13.2 Hz, $^3J_{8b,7}$ = 8.4 Hz, 1 H, 8-H_b), 2.62 (m, 2 H, 5-H), 3.84 (m, 2 H, 16-H), 4.17 (q, $^3J_{14,15}$ = 7.2 Hz, 2 H, 14-H), 7.12–7.22 (m, 3 H, 1-H, 3-H), 7.27 (m, 2 H, 2-H).
^{13}C-NMR (100 MHz, $CDCl_3$): δ = 14.3 (q, C-15), 15.7 (q, C-12), 20.6 (q, C-10), 21.5 (bs, C-7), 25.9 (t, C-19), 26.0 (t, C-18), 26.5 (t, C-20), 27.6 (t, C-19'), 28.5 (t, C-18'), 33.5 (t, C-6),

[6] K. Borszeky, T. Mallat, A. Baiker, *Tetrahedron: Asymmetry* **1997**, *8*, 3745–3753.

35.8 (t, C-5), 37.3 (t, C-8), 43.1 (d, C-17), 59.9 (t, C-14), 83.6 (d, C-16), 123.3 (s, C-11), 125.6 (d, C-1), 128.3 (d, C-2), 128.4 (d, C-3), 142.7 (s, C-4), 145.9 (s, C-9), 169.9 (s, C-12).

minor isomer (Z isomer, selected signals):

^{1}H-NMR (400 MHz, $CDCl_3$): δ = 2.01 (m, 3 H, 10-H), 3.80 (m, 2 H, 16-H), 4.12 (q, $^3J_{14,15}$ = 7.1 Hz, 2 H, 14-H).
The α-Isomer could not be detected.
HRMS (CI) calcd for $C_{31}H_{48}O_4B$ $[M+H]^+$: 495.3640, found: 495.3637.

Ethyl (2*E*,4*R*,5*S*)-5-[(4*S*,5*S*)-4,5-dicyclohexyl-1,3,2-dioxaborolan-2-yl]-2,4-dimethyl-7-phenylhept-2-enoate (5e)

Ethyl 2-metylpent-2-enoate **4e** was prepared as described by Andersson *et al.* [7]
382 mg (882 µmol) α-bromo boronic ester **2**, 157 mg (1.10 mmol) **4e**, 144 µl (ρ = 0.71 g/ml, 1.01 mmol) diisopropylamine, 606 µl (1.6 M in hexanes, 970 µmol) *n*-butyllithium and 159 µl (ρ = 1.06 g/ml, 1.32 mmol) DMPU were reacted according to GP 2. Flash chromatography (petroleum ether, ethyl acetate 95:5) gave **5e** as a mixture of isomers in 62 % yield (270 mg, 546 µmol) as a colorless oil, R_f = 0.27 (petroleum ether, ethyl acetate 95:5). To determine the d.r. and the γ:α ratio, the product mixture **5e** was directly converted into alcohol **6e** without further purification.
HRMS (CI) calcd for $C_{31}H_{48}O_4B$ $[M+H]^+$: 495.3640, found: 495.3640.

Ethyl (2*E*,4*R*,5*S*)-5-[(4*S*,5*S*)-4,5-dicyclohexyl-1,3,2-dioxaborolan-2-yl]-2,4-dimethyl-7-phenylhept-2-enoate (5f)

382 mg (882 µmol) α-bromo boronic ester **2**, 188 mg (1.10 mmol) **4f**, 144 µl (ρ = 0.71 g/ml, 1.01 mmol) diisopropylamine, 606 µl (1.6 M in hexanes, 970 µmol) *n*-butyllithium and 159 µl (ρ = 1.06 g/ml, 1.32 mmol) DMPU were reacted according to GP 2. Flash chromatography (petroleum ether, ethyl acetate 95:5) gave **5f** as a mixture of isomers in 59 % yield (270 mg, 526 µmol) as a colorless oil, R_f = 0.34 (petroleum ether, ethyl acetate 95:5). To determine the d.r. and the γ:α ratio, the product mixture **5f** was directly converted into alcohol **6f** without further purification.

***tert*-Butyl (2*E*,5*S*)-5-hydroxy-2-methyl-7-phenylhept-2-enoate (6a)**

247 mg (486 µmol) boronic ester **5a**, 226 µl (33 % in water, ρ = 1.11 g/ml, 2.43 mmol) hydrogen peroxide and 97 mg (2.43 mmol) sodium hydroxide were reacted according to GP 3. After 1 h, the reaction was worked up and the crude product was treated with 35 mg (583 µmol) methylboronic acid to separate (*S,S*)-DICHED. Purification by flash chromatography (petroleum ether, ethyl acetate 95:5, 8:2) gave **6a** (92 % *ee*) in 67 % yield (161 mg, 327 µmol) as a colorless oil, R_f = 0.29 (petroleum ether, ethyl acetate 8:2); $[\alpha]_D^{20}$ = +7.8 (c =1.0, $CHCl_3$).

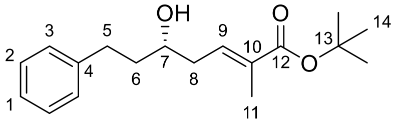

^{1}H-NMR (500 MHz, $CDCl_3$): δ = 1.49 (s, 9 H, 14-H), 1.63 (bs, 1 H, OH), 1.79–1.87 (m, 5 H, 6-H, 11-H), 2.36 (m, 2 H, 8-H), 2.70 (dt, $^2J_{5a,5b}$ = 13.9 Hz, $^3J_{5a,6}$ = 8.2 Hz, 1 H, 5-H_a), 2.82 (dt, $^2J_{5b,5a}$ = 14.2 Hz, $^3J_{5b,6}$ = 7.6 Hz, 1 H, 5-H_b), 3.78 (tt, $^3J_{7,6}$ ≈ $^3J_{7,8}$ = 6.1 Hz, 1 H, 7-H), 6.70 (tq, $^3J_{9,8}$ = 7.6 Hz, $^4J_{9,11}$ = 1.4 Hz, 1 H, 9-H), 7.17–7.24 (m, 3 H, 1-H, 3-H), 7.29 (m, 2 H, 2-H).

[7] J.-Q. Li, X. Quan, P. G. Andersson, *Chem. Eur. J.* **2012**, *18*, 10609–10616.

^{13}C-NMR (125 MHz, $CDCl_3$): δ = 12.7 (q, C-11), 28.1 (q, C-14), 32.0 (t, C-5), 36.9 (t, C-8), 38.7 (t, C-6), 70.4 (d, C-7), 80.2 (s, C-13), 125.9 (d, C-1), 128.4 (d, C-2), 128.4 (d, C-3), 131.7 (s, C-10), 136.4 (d, C-9), 141.7 (s, C-4), 167.2 (s, C-12).
HPLC (*Chiralcel OD-H 5 µm*, hexane/*i*PrOH 9:1, 1.0 ml/min, 20 °C): t_R(*S*)-**6a** = 9.76 min, t_R(*R*)-**6a** = 12.85 min.
HRMS (CI) calcd for $C_{18}H_{27}O_3$ $[M+H]^+$: 291.1955, found: 291.1959.

Ethyl (2*E*,5*S*)-5-hydroxy-2-methyl-7-phenylhept-2-enoate (6b)

68 mg (142 µmol) boronic ester **5b**, 66 µl (33 % in water, ρ = 1.11 g/ml, 708 µmol) hydrogen peroxide and 75 mg (708 µmol) sodium carbonate were reacted according to GP 3. After 1.5 h, the reaction was worked up and the crude product was treated with 10 mg (170 µmol) methylboronic acid to separate (*S*,*S*)-DICHED. Purification by flash chromatography (petroleum ether, ethyl acetate 8:2) gave **6b** (94 % *ee*) in 75 % yield (28 mg, 107 µmol) as a colorless oil, R_f = 0.26 (petroleum ether, ethyl acetate 8:2); $[\alpha]_D^{20}$ = +6.5 (c =1.0, $CHCl_3$).

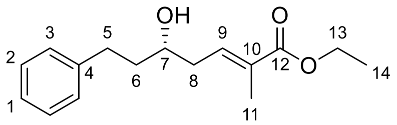

^{1}H-NMR (400 MHz, $CDCl_3$): δ = 1.28 (t, $^3J_{14,13}$ = 7.0 Hz, 3 H, 14-H), 1.70–1.93 (m, 6 H, 6-H, 11-H, OH), 2.37 (dd, $^3J_{8,7} \approx {}^3J_{8,9}$ = 6.5 Hz, 2 H, 8-H), 2.68 (dt, $^2J_{5a,5b}$ = 13.8 Hz, $^3J_{5a,6}$ = 8.2 Hz, 1 H, 5-H_a), 2.81 (dt, $^2J_{5b,5a}$ = 14.4 Hz, $^3J_{5b,6}$ = 7.7 Hz, 1 H, 5-H_b), 3.78 (tt, $^3J_{7,6} \approx {}^3J_{7,8}$ = 5.9 Hz, 1 H, 7-H), 4.18 (q, $^3J_{13,14}$ = 7.1 Hz, 2 H, 13-H), 6.80 (t, $^3J_{9,8}$ = 7.2 Hz, 1 H, 9-H), 7.11–7.23 (m, 3 H, 1-H, 3-H), 7.28 (m, 2 H, 2-H).
^{13}C-NMR (100 MHz, $CDCl_3$): δ = 12.7 (q, C-11), 14.2 (q, C-14), 32.0 (t, C-5), 36.9 (t, C-8), 38.7 (t, C-6), 60.6 (t, C-13), 70.3 (d, C7), 125.9 (d, C-1), 128.4 (d, C-2), 128.4 (d, C-3), 130.1 (s, C-10), 137.6 (d, C-9), 141.7 (s, C-4), 167.9 (s, C-12).
HPLC (*Chiralcel OD-H 5 µm*, hexane/*i*PrOH 9:1, 1.0 ml/min, 20 °C): t_R(*S*)-**6b** = 14.84 min, t_R(*R*)-**6a** = 20.73 min.
HRMS (CI) calcd for $C_{16}H_{23}O_3$ $[M+H]^+$: 263.1642, found: 263.1659.

(2*E*,5*S*)-5-hydroxy-2-methyl-7-phenylhept-2-enoic acid (6c)

61 mg (131 µmol) boronic ester **5a**, 61 µl (33 % in water, ρ = 1.11 g/ml, 654 µmol) hydrogen peroxide and 26 mg (654 µmol) sodium hydroxide were reacted according to GP 3. After 4 d, saturated $NaHCO_3$ solution was added and the mixture was washed twice with diethyl ether. The aqueous layer was acidified to pH = 2 with 1 M HCl and extracted three times with ethyl acetate. The combined organic extracts were dried over Na_2SO_4 and concentrated in vacuo. Purification by reversed phase flash chromatography (H_2O + 0.1 % HCOOH → 50 % MeCN) gave **6c** in 72 % yield (22 mg, 94 µmol) as a colorless oil, R_f = 0.06 (petroleum ether, ethyl acetate 8:2); $[\alpha]_D^{20}$ = +6.5 (c =1.0, $CHCl_3$).

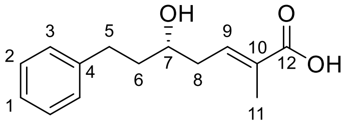

^{1}H-NMR (400 MHz, acetone-d_6): δ = 1.70–1.87 (m, 5 H, 6-H, 11-H), 2.40 (m, 2 H, 8-H), 2.69 (ddd, $^2J_{5a,5b}$ = 13.6 Hz, $^3J_{5a,6a}$ = 9.2 Hz, $^3J_{5a,6b}$ = 7.3 Hz, 1 H, 5-H_a), 2.83 (ddd, $^2J_{5b,5a}$ = 13.7 Hz, $^3J_{5b,6b}$ = 9.5 Hz, $^3J_{5b,6a}$ = 6.0 Hz, 1 H, 5-H_b), 3.76 (m, 1 H, 7-H), 6.93 (tq, $^3J_{9,8}$ = 7.3 Hz, $^4J_{9,11}$ = 0.9 Hz, 1 H, 9-H), 7.11–7.31 (m, 5 H, 1-H, 2-H, 3-H).

^{13}C-NMR (100 MHz, acetone-d$_6$): δ = 12.8 (q, C-11), 32.8 (t, C-5), 37.8 (t, C-8), 40.2 (t, C-6), 70.4 (d, C-7), 126.5 (d, C-1), 129.2 (d, C-2), 129.3 (d, C-3), 129.5 (s, C-10), 140.3 (d, C-9), 143.5 (s, C-4), 169.3 (s, C-12).
HRMS (CI) calcd for $C_{14}H_{17}O_2$ $[M+H–H_2O]^+$: 217.1223, found: 217.1229.

Ethyl (*S*,*E*)-5-hydroxy-2,3-dimethyl-7-phenylhept-2-enoate (6d)

76 mg (154 µmol) boronic ester **5d**, 71 µl (33 % in water, ρ = 1.11 g/ml, 768 µmol) hydrogen peroxide and 81 mg (768 µmol) sodium carbonate were reacted according to GP 3. After 1.5 h, the reaction was worked up and the crude product was purified by reversed phase flash chromatography (H_2O/MeCN 9:1 → 5:95) to yield a mixture of *E* and *Z* isomers containing (*S*,*S*)-DICHED as an impurity. Further purification by preparative HPLC (*Phenomenex Luna® C18(2)*, H_2O + 0.1 % HCOOH, MeCN 9:1 → 5:95) gave **6d** (single isomer) in 66 % yield (28 mg, 101 µmol) as a colorless oil, R_f = 0.31 (petroleum ether, ethyl acetate 8:2); $[\alpha]_D^{20}$ = –3.7 (c =1.0, $CHCl_3$).

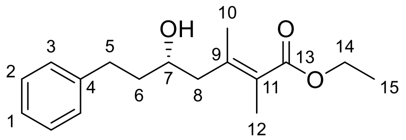

^{1}H-NMR (400 MHz, $CDCl_3$): δ = 1.29 (t, $^3J_{15,14}$ = 7.2 Hz, 3 H, 15-H), 1.82 (m, 2 H, 6-H), 1.85–1.93 (m, 4 H, 12-H, OH), 1.95 (m, 3 H, 10-H), 2.21 (dd, $^2J_{8a,8b}$ = 13.2 Hz, $^3J_{8a,7}$ = 4.3 Hz, 1 H, 8-H$_a$), 2.45 (dd, $^2J_{8b,8a}$ = 13.2 Hz, $^3J_{8b,7}$ = 8.8 Hz, 1 H, 8-H$_b$), 2.69 (dt, $^2J_{5a,5b}$ = 14.0 Hz, $^3J_{5a,6}$ = 8.1 Hz, 1 H, 5-H$_a$), 2.83 (dt, $^2J_{5b,5a}$ = 14.1 Hz, $^3J_{5b,6}$ = 7.5 Hz, 1 H, 5-H$_b$), 3.83 (m, 1 H, 7-H), 4.19 (q, $^3J_{14,15}$ = 7.1 Hz, 2 H, 14-H), 7.14–7.23 (m, 3 H, 1-H, 3-H), 7.27 (m, 2 H, 2-H).
^{13}C-NMR (100 MHz, $CDCl_3$): δ = 14.2 (q, C-15), 15.9 (q, C-12), 21.3 (q, C-10), 32.1 (t, C-5), 38.9 (t, C-6), 43.6 (t, C-8), 60.2 (t, C-14), 69.5 (d, C-7), 125.8 (s, C-4, C-11), 128.4 (d, C-2,C-3), 141.3 (s, C-9), 141.8 (s, C-4), 170.0 (s, C-13).
HRMS (CI) calcd for $C_{17}H_{25}O_3$ $[M+H]^+$: 277.1798, found: 277.1794.

Ethyl (2*E*,4*S*,5*S*)-5-hydroxy-2,4-dimethyl-7-phenylhept-2-enoate (6e)

254 mg (514 µmol) boronic ester **5e** (mixture of isomers), 238 µl (33 % in water, ρ = 1.11 g/ml, 2.57 mmol) hydrogen peroxide and 272 mg (2.57 mmol) sodium carbonate were reacted according to GP 3. After 1.5 h, the reaction was worked up and the crude product was purified by reversed phase flash chromatography (H_2O/MeCN 9:1 → 5:95) to yield a mixture of isomers (γ:α = 78:22, d.r. = 95:5 according to ^{1}H NMR) containing (*S*,*S*)-DICHED as an impurity. Further purification by preparative HPLC (*Phenomenex Luna® C18(2)*, H_2O + 0.1 % HCOOH, MeCN 9:1 → 5:95) gave **6e** (single isomer) in 39 % yield (55 mg, 199 µmol) as a colorless oil, R_f = 0.33 (petroleum ether, ethyl acetate 8:2); $[\alpha]_D^{20}$ = –27.2 (c =1.0, $CHCl_3$).

major isomer (γ-(4S,5S) isomer):

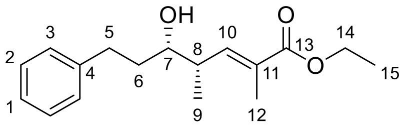

^{1}H-NMR (500 MHz, $CDCl_3$): δ = 1.06 (d, $^3J_{9,8}$ = 6.9 Hz, 3 H, 9-H), 1.30 (t, $^3J_{15,14}$ = 6.9 Hz, 3 H, 15-H), 1.59 (bs, 1 H, OH), 1.68 (m, 1 H, 6-H$_a$), 1.82 (m, 1 H, 6-H$_b$), 1.85 (d, $^4J_{12,10}$ = 1.6 Hz, 3 H, 12-H), 2.59 (m, 1 H, 8-H), 2.66 (ddd, $^2J_{5a,5b}$ = 13.8 Hz, $^3J_{5a,6a}$ = 9.5 Hz, $^3J_{5a,6b}$ = 6.6 Hz, 1 H, 5-H$_a$), 2.84 (ddd, $^2J_{5b,5a}$ = 14.2 Hz, $^3J_{5b,6b}$ = 10.1 Hz, $^3J_{5a,6b}$ = 5.4 Hz, 1 H, 5-H$_b$),

3.52 (ddd, $^{3}J_{7,6a}$ = 9.5 Hz, $^{3}J_{7,8}$ = 6.6 Hz, $^{3}J_{7,6b}$ = 2.8 Hz, 1 H, 7-H), 4.19 (q, $^{3}J_{14,15}$ = 7.3 Hz, 2 H, 14-H), 6.60 (dq, $^{3}J_{10,8}$ = 10.4 Hz, $^{4}J_{10,12}$ = 1.4 Hz, 1 H, 10-H), 7.17–7.22 (m, 3 H, 1-H, 3-H), 7.28 (m, 2 H, 2-H).
^{13}C-NMR (125 MHz, $CDCl_3$): δ = 12.7 (q, C-12), 14.3 (q, C-15), 15.4 (q, C-9), 32.3 (t, C-5), 36.5 (t, C-6), 39.7 (d, C-8), 60.6 (t, C-14), 74.7 (d, C-7), 125.9 (d, C-1), 128.0 (s, C-11), 128.4 (d, C-3), 128.4 (d, C-2), 141.8 (s, C-4), 143.5 (d, C-10), 168.2 (s, C-13).
HRMS (CI) calcd for $C_{17}H_{25}O_3$ $[M+H]^+$: 277.1798, found: 277.1838.

minor isomer (γ-(4R,5S) isomer, selected signals):

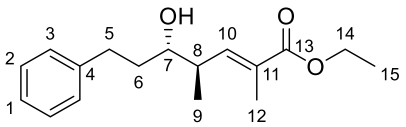

^{1}H-NMR (500 MHz, $CDCl_3$): δ = 6.68 (dq, $^{3}J_{10,8}$ = 10.4 Hz, $^{4}J_{10,12}$ = 1.4 Hz, 1 H, 10-H).

minor isomer (α isomer, mixture of diastereomers and E/Z isomers, selected signals):

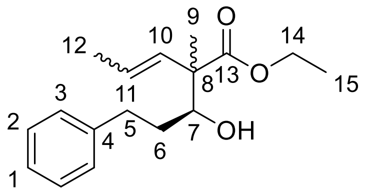

^{1}H-NMR (500 MHz, $CDCl_3$): δ = 5.44–5.73 (m, 2 H, 10-H, 11-H).

To confirm the configuration of **6e**, it was converted into known diol **6ea** via ozonolysis / reduction.

(2*S*,3*S*)-2-Methyl-5-phenylpentane-1,3-diol (6ea)

Ozone was bubbled through a solution of 11 mg (40 µmol, 1.0 eq.) **6e** in 1 ml DCM at –78 °C until the solution was colored blue (approximately 5 min). Excess ozone was removed by bubbling through with oxygen and 13 mg (48 µmol, 1.2 eq.) triphenylphosphine were added. The mixture was warmed to room temperature over 15 min and the solvent was removed in vacuo. The residue was passed through a short silica gel pad (pentane, diethyl ether 1:1) to yield the crude aldehyde which was directly used in the next step.

To a solution of the aldehyde in 1 ml methanol were added 2 mg (48 µmol, 1.2 eq.) sodium borohydride at room temperature. After stirring for 16 h, brine was added and the mixture was extracted three times with DCM. The combined organic layers were dried over Na_2SO_4 and concentrated in vacuo to give **6ea** in 39 % yield (3 mg, 15 µmol) as a colorless oil, R_f = 0.41 (Et_2O); $[\alpha]_D^{20}$ = –20.4 (c =1.0, $CHCl_3$).

NMR spectra of **4a** were in accordance with previoulsy published data.[8]

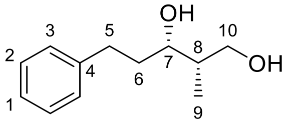

^{1}H-NMR (400 MHz, $CDCl_3$): δ = 0.92 (d, $^{3}J_{9,8}$ = 7.1 Hz, 3 H, 9-H), 1.66–1.94 (m, 3 H, 6-H, 8-H), 2.58 (bs, 2 H, OH), 2.65 (ddd, $^{2}J_{5a,5b}$ = 13.8 Hz, $^{3}J_{5a,6a}$ = 9.5 Hz, $^{3}J_{5a,6b}$ = 6.8 Hz, 1 H, 5-H_a), 2.84 (ddd, $^{2}J_{5b,5a}$ = 13.9 Hz, $^{3}J_{5b,6b}$ = 9.9 Hz, $^{3}J_{5b,6a}$ = 5.5 Hz, 1 H, 5-H_b), 3.70 (d, $^{3}J_{10,8}$ = 5.5 Hz, 2 H, 10-H), 3.85 (m, 1 H, 7-H), 7.16–7.24 (m, 3 H, 1-H, 3-H), 7.29 (m, 2 H, 2-H).
^{13}C-NMR (100 MHz, $CDCl_3$): δ = 10.3 (q, C-9), 32.6 (t, C-5), 35.7 (t, C-6), 39.2d, C-8), 67.0 (t, C-10), 73.9 (d, C-7), 125.8 (d, C-1), 128.4 (d, C-2, C-3), 142.0 (s, C-4).

---

8 R. L. Danheiser, D. J. Carini, C. A. Kwasigroch, *J. Org. Chem.* **1986**, *51*, 3870–3878.

HRMS (CI) calcd for $C_{12}H_{16}O$ $[M–H_2O]^+$: 176.1196, found: 176.1197.

***tert*-Butyl (2*E*,4*S*,5*S*)-5-hydroxy-2,4-dimethyl-7-phenylhept-2-enoate (6f)**

256 mg (490 µmol) boronic ester **5f** (mixture of isomers), 227 µl (33 % in water, ρ = 1.11 g/ml, 2.45 mmol) hydrogen peroxide and 98 mg (2.45 mmol) sodium hydroxide were reacted according to GP 3. After 1.5 h, the reaction was worked up and the crude product was purified by reversed phase flash chromatography (H_2O/MeCN 9:1 → 5:95) to yield a mixture of isomers (γ:α = 85:15, d.r. = 83:17 according to ^{1}H NMR) containing (*S*,*S*)-DICHED as an impurity. Further purification by preparative HPLC (*Phenomenex Luna® C18(2)*, H_2O + 0.1 % HCOOH, MeCN 9:1 → 5:95) gave **6f** (single isomer) in 37 % yield (55 mg, 181 µmol) as a colorless oil, R_f = 0.38 (petroleum ether, ethyl acetate 8:2); $[\alpha]_D^{20}$ = –28.1 (c =1.0, $CHCl_3$).

major isomer (γ-(4S,5S) isomer):

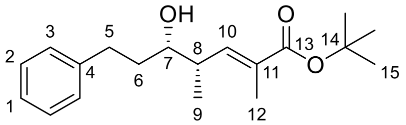

^{1}H-NMR (400 MHz, $CDCl_3$): δ = 1.05 (d, $^3J_{9,8}$ = 6.7 Hz, 3 H, 9-H), 1.48 (m, 10 H, 15-H, OH), 1.67 (m, 1 H, 6-H_a), 1.77–1.88 (m, 4 H, 6-H_b. 12-H), 2.56 (m, 1 H, 8-H), 2.66 (ddd, $^2J_{5a,5b}$ = 13.8 Hz, $^3J_{5a,6a}$ = 9.5 Hz, $^3J_{5a,6b}$ = 6.8 Hz, 1 H, 5-H_a), 2.84 (ddd, $^2J_{5b,5a}$ = 13.9 Hz, $^3J_{5b,6b}$ = 9.7 Hz, $^3J_{5a,6b}$ = 5.3 Hz, 1 H, 5-H_b), 3.50 (m, 1 H, 7-H), 6.49 (dq, $^3J_{10,8}$ = 10.3 Hz, $^4J_{10,12}$ = 1.3 Hz, 1 H, 10-H), 7.15–7.23 (m, 3 H, 1-H, 3-H), 7.28 (m, 2 H, 2-H).
^{13}C-NMR (100 MHz, $CDCl_3$): δ = 12.8 (q, C-12), 15.4 (q, C-9), 28.1 (q, C-15), 32.3 (t, C-5), 36.5 (t, C-6), 39.7 (d, C-8), 74.8 (d, C-7), 80.2 (s, C-14), 125.9 (d, C-1), 128.4 (d, C-3), 128.4 (d, C-11), 129.5 (s, C-11), 141.8 (s, C-4), 142.3 (d, C-10), 167.5 (s, C-13).
HRMS (CI) calcd for $C_{19}H_{29}O_3$ $[M+H]^+$: 305.2111, found: 305.2101.

minor isomer (γ-(4R,5S) isomer, selected signals):

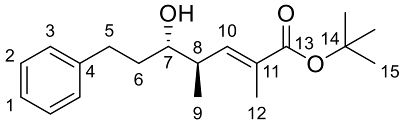

^{1}H-NMR (500 MHz, $CDCl_3$): δ = 6.56 (dq, $^3J_{10,8}$ = 10.2 Hz, $^4J_{10,12}$ = 1.3 Hz, 1 H, 10-H).

minor isomer (α isomer, mixture of diastereomers and E/Z isomers selected signals):

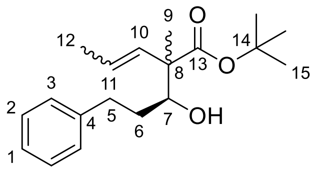

^{1}H-NMR (500 MHz, $CDCl_3$): δ = 5.45–5.71 (m, 2 H, 10-H, 11-H).

To confirm the configuration of **6f**, it was also converted into known diol **6ea** via ozonolysis / reduction.

Preparation of several Alkylboronates

(4*S*,5*S*)-4,5-Diphenyl-2-propyl-1,3,2-dioxaborolane (A')

(*S*,*S*)-Hydrobenzoin was prepared as described by Sharpless *et al.*[9]

1.80 g (20.5 mmol, 1.2 eq.) propylboronic acid and 3.66 g (17.1 mmol, 1.0 eq.) (*S*,*S*)-hydrobenzoin were dissolved in 90 ml diethyl ether. After the addition of 5.6 g anhydrous $MgSO_4$, the mixture was stirred 2 d at room temperature, filtered and concentrated in vacuo. Filtration through a short silica gel pad (petroleum ether, diethyl ether 9:1) gave **A'** in 99 % yield (4.48 g, 16.8 mmol) as a colorless oil, R_f = 0.31 (petroleum ether, diethyl ether 9:1); $[\alpha]_D^{20}$ = –36.4 (c =1.0, $CHCl_3$).

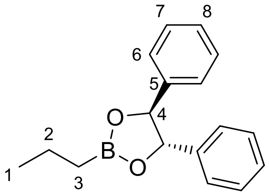

^{1}H-NMR (400 MHz, $CDCl_3$): δ = 0.96–1.10 (m, $^3J_{3,2}$ = 7.8 Hz, $^3J_{1,2}$ = 7.3 Hz, 5 H, 1-H, 3-H), 1.59 (tq, $^3J_{2,1} \approx {}^3J_{2,3}$ = 7.5 Hz, 2 H, 7-H), 5.11 (s, 2 H, 4-H), 7.25 (m, 4 H, 6-H), 7.28–7.42 (m, 6 H, 7-H, 8-H).
^{13}C-NMR (100 MHz, $CDCl_3$): δ = 13.0 (bs, C-3), 17.0 (q, C-1), 17.5 (t, C-2), 86.3 (d, C-4), 125.7 (d, C-6), 128.3 (d, C-8), 128.8 (d, C-7), 140.6 (s, C-5).
HRMS (CI) calcd for $C_{17}H_{19}O_2B$ $[M]^+$: 266.1473, found: 266.1480.

(4*S*,5*S*)-4,5-Dicyclohexyl-2-propyl-1,3,2-dioxaborolane (A)

To a solution of 4.45 g (16.7 mmol, 1.0 eq.) of boronic ester **A'** in 14 ml Methanol were added 44 mg (209 µmol, 1.25 mol-%) rhodium(III)-chloride hydrate and 504 mg Al_2O_3 (basic, activity state I). The mixture was stirred under an atmosphere of hydrogen (80 bar) at room temperature. After 5 h, the mixture was filtered through celite and concentrated in vacuo. Filtration of the residue through a short silica gel pad (petroleum ether, diethyl ether 96:4) gave **A** in 86 % yield (4.02 g, 14.5 mmol) as a colorless oil, R_f = 0.51 (petroleum ether, diethyl ether 9:1); $[\alpha]_D^{20}$ = –41.1 (c =1.0, $CHCl_3$).

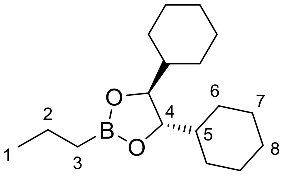

^{1}H-NMR (400 MHz, $CDCl_3$): δ = 0.80 (t, $^3J_{3,2}$ = 7.7 Hz, 2 H, 3-H), 0.89–1.02 (m, $^3J_{1,2}$ = 7.3 Hz, 5 H, 1-H, 7-H), 1.06 (m, 2 H, 6-H), 1.11–1.25 (m, 6 H, 6-H', 7-H'), 1.31 (m, 2 H, 5-H), 1.45 (tq, $^3J_{2,1} \approx {}^3J_{2,3}$ = 7.5 Hz, 2 H, 2-H), 1.59 (m, 2 H, 6-H''), 1.67 (m, 2 H, 8-H), 1.71–1.83 (m, 6 H, 6-H''', 7-H'', 8-H'), 3.83 (m, 2 H, 4-H).
^{13}C-NMR (100 MHz, $CDCl_3$): δ = 12.6 (bs, C-3), 17.0 (q, C-1), 17.6 (t, C-2), 25.9 (t, C-7), 26.0 (t, C-6), 26.5 (t, C-8), 27.4 (t, C-7'), 28.3 (t, C-6'), 43.0 (d, C-5), 83.1 (d, C-4).
HRMS (CI) calcd for $C_{17}H_{32}O_2B$ $[M+H]^+$: 279.2490, found: 279.2479.

---

[9] Z.-M. Wang, K. B. Sharpless, *J. Org. Chem.* **1994**, *59*, 8302–8303.

(4*S*,5*S*)-2-Isobutyl-4,5-diisopropyl-1,3,2-dioxaborolane (B)

(*S*,*S*)-diisopropylethanediol (DIPED) was prepared as described previously.[10]

140 mg (1.37 mmol, 1.2 eq.) isobutylboronic acid and 167 mg (1.14 mmol, 1.0 eq.) (*S*,*S*)-DIPED were dissolved in 6 ml diethyl ether. After the addition of 375 mg anhydrous $MgSO_4$, the mixture was stirred 1.5 h at room temperature, filtered and concentrated in vacuo (40 °C, > 200 mbar). Filtration through a short silica gel pad (pentane, diethyl ether 9:1) gave boronic ester **B** in 93 % yield (225 mg, 1.06 mmol) as a colorless oil, R_f = 0.64 (pentane, diethyl ether 9:1); $[\alpha]_D^{20}$ = –59.1 (c =1.0, $CHCl_3$).

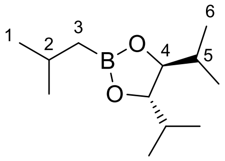

^{1}H-NMR (400 MHz, $CDCl_3$): δ = 0.79 (d, $^3J_{3,2}$ = 7.0 Hz, 2 H, 3-H), 0.91 (d, $^3J_{6,5}$ = 6.7 Hz, 6 H, 6-H), 0.91 (d, $^3J_{6',5}$ = 6.8 Hz, 6 H, 6-H'), 0.94 (d, $^3J_{1,2}$ = 6.6 Hz, 3 H, 1-H), 0.94 (d, $^3J_{1',2}$ = 6.6 Hz, 3 H, 1-H'), 1.66 (m, 2 H, 5-H), 1.87 (qqt, $^3J_{2,1}$ ≈ $^3J_{2,1'}$ ≈ $^3J_{2,3}$ = 6.7 Hz, 1 H, 2-H), 3.80 (m, 2 H, 4-H).
^{13}C-NMR (100 MHz, $CDCl_3$): δ = 16.7 (q, C-6), 17.9 (q, C-6'), 20.8 (bs, C-3), 24.8 (d, C-2), 25.1 (q, C-1), 25.3 (q, C-1'), 33.1 (d, C-5), 83.9 (d, C-4).

***tert*-Butyl{4-[(4*S*,5*S*)-4,5-dicyclohexyl-1,3,2-dioxaborolan-2-yl]butoxy}dimethylsilane (C)**

tert-butyldimethyl[4-(4,4,5,5-tetramethyl-1,3,2-dioxaborolan-2-yl)butoxy]silane[11] and (*S*,*S*)-dicyclohexylethanediol (DICHED)[12] were prepared as described previously.

To a solution of 10.4 g (33.0 mmol, 1.0 eq.) *tert*-butyldimethyl[4-(4,4,5,5-tetramethyl-1,3,2-dioxaborolan-2-yl)butoxy]silane in 160 ml diethyl ether were added 7.47 g (33.0 mmol, 1.0 eq.) (*S*,*S*)-DICHED and 4 ml water at room temperature. After stirring for 9 d, the mixture was concentrated in vacuo. Purification of the residue by flash chromatography (petroleum ether, ethyl acetate 98.5:1.5) gave boronic ester **C** in 84 % yield (11.7 g, 27.7 mmol) as a colorless oil, R_f = 0.36 (petroleum ether, ethyl acetate 9:1); $[\alpha]_D^{20}$ = –17.1 (c =1.0, $CHCl_3$).

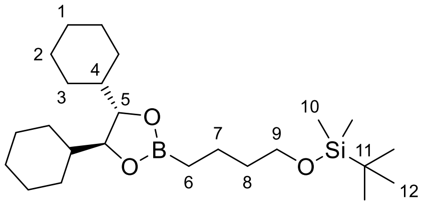

^{1}H-NMR (400 MHz, $CDCl_3$): δ = 0.04 (s, 6 H, 10-H), 0.81 (t, $^3J_{6,7}$ = 7.6 Hz, 2 H, 6-H), 0.89 (s, 9 H, 12-H), 0.96 (m, 2 H, 2-H), 1.06 (m, 2 H, 3-H), 1.11–1.25 (m, 6 H, 2-H', 3-H'), 1.31 (m, 2 H, 4-H), 1.46 (m, 2 H, 7-H), 1.50–1.63 (m, 4 H, 3-H'', 8-H), 1.67 (m, 2 H, 1-H), 1.71–1.83 (m, 6 H, 1-H', 2-H'', 3-H'''), 3.60 (t, $^3J_{9,8}$ = 6.5 Hz, 2 H, 9-H), 3.82 (m, 2 H, 5-H).
^{13}C-NMR (100 MHz, $CDCl_3$): δ = –5.3 (q, C-10), 10.1 (bs, C-6), 18.4 (s, C-11), 20.4 (t, C-7), 25.9 (t, C-2), 26.0 (q, C-12), 26.0 (t, C-3), 26.5 (t, C-1), 27.3 (t, C-2'), 28.4 (t, C-3'), 35.5 (t, C-8), 43.0 (d, C-4), 63.1 (t, C-9), 83.2 (d, C-4).
HRMS (CI) calcd for $C_{24}H_{48}O_3BSi$ $[M+H]^+$: 423.3460, found: 423.3465.

---

[10] D. S. Matteson, E. C. Beedle, A. A. Kandil, *J. Org. Chem.* **1987**, *52*, 5034–5036. K. Bojaryn, C. Hoffmann, F. R. Struth, C. Hirschhäuser, *Synlett* **2018**, *29*, 1092–1094.
[11] S. D. Dreher, S.-E. Lim, D. L. Sandrock, G. A. Molander, *J. Org. Chem.* **2009**, *74*, 3626–3631.
[12] W. C. Hiscox, D. S. Matteson, *J. Org. Chem.* **1996**, *61*, 8315–8316.

(4*S*,5*S*)-2-[(*S*)-1-(Benzyloxy)butyl]-4,5-dicyclohexyl-1,3,2-dioxaborolane (D')

According to GP4, 1.00 g (3.59 mmol) boronic ester **A** were treated with 694 µl (ρ = 1.32 g/ml, 10.7 mmol) dichloromethane, 2.81 ml (1.6 M in hexane, 4.49 mmol) *n*-butyllithium, 692 µl (ρ = 0.71 g/ml, 4.85 mmol) diisopropylamine and 980 mg (7.19 mmol) zinc chloride. The nucleophile solution was prepared by adding 523 µl (ρ = 1.04 g/ml, 5.03 mmol, 1.4 eq.) benzyl alcohol to a suspension of 187 mg (60 % in mineral oil, 4.67 mmol, 1.3 eq.) sodium hydride in 2.2 ml anhydrous THF / 6.5 ml anhydrous DMSO and stirring at room temperature for 9 h. After addition of the nucleophile to the α-chloro boronic ester solution, the mixture was stirred for 16 h at room temperature, worked up and purified by flash chromatography (petroleum ether, ethyl acetate 97:3) to give **D'** in 79 % yield (1.13 g, 2.84 mmol) as a colorless oil, R_f = 0.18 (petroleum ether, ethyl acetate 97:3); $[\alpha]_D^{20}$ = –22.2 (c =1.0, $CHCl_3$).

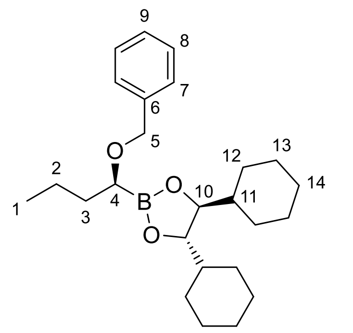

^{1}H-NMR (400 MHz, $CDCl_3$): δ = 0.91 (t, $^3J_{1,2}$ = 7.3 Hz, 3 H, 1-H), 0.98 (m, 2 H, 13-H), 1.08 (m, 2 H, 12-H), 1.15–1.29 (m, 6 H, 12-H‘, 13-H‘), 1.35 (m, 2 H, 11-H), 1.45 (m, 2 H, 2-H), 1.56–1.72 (m, 6 H, 3-H, 12-H‘‘, 14-H), 1.72–1.86 (m, 6 H, 12-H‘‘‘, 13-H‘‘, 14-H‘), 3.35 (dd, $^3J_{4,3a}$ = 7.6 Hz, $^3J_{4,3b}$ = 6.0 Hz, 1 H, 4-H), 3.91 (m, 2 H, 10-H), 4.48 (d, $^2J_{5a,5b}$ = 12.0 Hz, 1 H, 5-H_a), 4.60 (d, $^2J_{5b,5a}$ = 11.7 Hz, 1 H, 5-H_b), 7.25 (m, 1 H, 9-H), 7.29–7.41 (m, 4 H, 7-H, 8-H). **^{13}C-NMR** (100 MHz, $CDCl_3$): δ = 14.1 (q, C-1), 19.8 (t, C-2), 25.9 (t, C-13), 26.0 (t, C-12), 26.4 (t, C-14), 27.4 (t, C-13‘), 28.3 (t, C-12‘), 33.8 (t, C-3), 42.9 (d, C-11), 67.6 (bs, C-4), 72.2 (t, C-5), 83.6 (d, C-10), 127.3 (d, C-9), 127.8 (d, C-7), 128.2 (d, C-8), 139.3 (s, C-6).

(4*S*,5*S*)-2-[(2*R*,3*R*)-3-(Benzyloxy)hexan-2-yl]-4,5-dicyclohexyl-1,3,2-dioxaborolane (D)

According to GP4, 600 mg (1.51 mmol) **D'** were treated with 291 µl (ρ = 1.32 g/ml, 4.52 mmol) dichloromethane, 1.18 ml (1.6 M in hexane, 1.18 mmol) *n*-butyllithium, 290 µl (ρ = 0.71 g/ml, 2.03 mmol) diisopropylamine and 616 mg (4.52 mmol) zinc chloride. To the α-chloro boronic ester solution were added 1.51 ml (3.0 M in THF, 4.52 mmol) methylmagnesium chloride and the mixture was stirred for 3 d at room temperature, worked up and purified by flash chromatography (petroleum ether, ethyl acetate 97:3) to give **D** in 70 % yield (450 mg, 1.06 mmol) as a colorless oil, R_f = 0.21 (petroleum ether, ethyl acetate 97:3); $[\alpha]_D^{20}$ = –26.8 (c =1.0, $CHCl_3$).

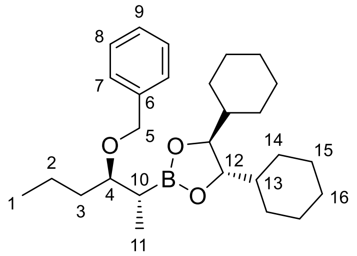

^{1}H-NMR (400 MHz, $CDCl_3$): δ = 0.91 (t, $^3J_{1,2}$ = 7.3 Hz, 3 H, 1-H), 0.98 (m, 2 H, 13-H), 1.08 (m, 2 H, 12-H), 1.15–1.29 (m, 6 H, 12-H‘, 13-H‘), 1.35 (m, 2 H, 11-H), 1.45 (m, 2 H, 2-H), 1.56–1.72 (m, 6 H, 3-H, 12-H‘‘, 14-H), 1.72–1.86 (m, 6 H, 12-H‘‘‘, 13-H‘‘, 14-H‘), 3.35 (dd,

$^{3}J_{4,3a}$ = 7.6 Hz, $^{3}J_{4,3b}$ = 6.0 Hz, 1 H, 4-H), 3.91 (m, 2 H, 10-H), 4.48 (d, $^{2}J_{5a,5b}$ = 12.0 Hz, 1 H, 5-H$_a$), 4.60 (d, $^{2}J_{5b,5a}$ = 11.7 Hz, 1 H, 5-H$_b$), 7.25 (m, 1 H, 9-H), 7.29–7.41 (m, 4 H, 7-H, 8-H).
^{13}C-NMR (100 MHz, $CDCl_3$): δ = 14.1 (q, C-1), 19.8 (t, C-2), 25.9 (t, C-13), 26.0 (t, C-12), 26.4 (t, C-14), 27.4 (t, C-13'), 28.3 (t, C-12'), 33.8 (t, C-3), 42.9 (d, C-11), 67.6 (bs, C-4), 72.2 (t, C-5), 83.6 (d, C-10), 127.3 (d, C-9), 127.8 (d, C-7), 128.2 (d, C-8), 139.3 (s, C-6).

(4S,5S)-2,4,5-Tricyclohexyl-1,3,2-dioxaborolane (E)

500 mg (3.91 mmol, 1.2 eq.) cyclohexylboronic acid and 737 mg (3.26 mmol, 1.0 eq.) (*S*,*S*)-DICHED were dissolved in 17 ml diethyl ether. After the addition of 890 mg anhydrous $MgSO_4$, the mixture was stirred 16 h at room temperature, filtered and concentrated in vacuo. Filtration through a short silica gel pad (pentane, diethyl ether 98:2) gave **E** in 100 % yield (1.04 g, 3.26 mmol) as colorless needles, R_f = 0.50 (pentane, diethyl ether 98:2); m.p. 44–45 °C; $[\alpha]_D^{20}$ = –46.7 (c =1.0, $CHCl_3$).

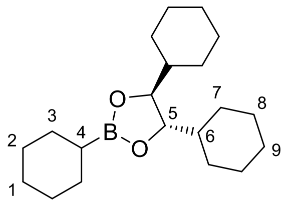

^{1}H-NMR (400 MHz, $CDCl_3$): δ = 0.88 (m, 1 H, 4-H), 0.96 (m, 2 H, 8-H), 1.01–1.40 (m, 15 H, 1-H, 2-H, 3-H, 6-H, 7-H, 8-H'), 1.52–1.85 (m, 15 H, 1-H', 2-H', 3-H', 7-H', 8-H'', 9-H), 3.82 (m, 2 H, 5-H).
^{13}C-NMR (100 MHz, $CDCl_3$): δ = 25.9 (t, C-8), 26.0 (t, C-7), 26.5 (t, C-9), 26.8 (t, C-1), 27.2 (t, C-2/C-3), 27.3 (t, C-8'), 28.2 (t, C-7'), 28.2 (t, C-2/3), 28.3 (t, C-2'/3'), 43.0 (d, C-6), 83.0 (d, C-5).

(4*S*,5*S*)-4,5-Dicyclohexyl-2-[(*R*)-hexan-3-yl]-1,3,2-dioxaborolane (F)

According to GP4, 420 mg (1.51 mmol) **A** were treated with 291 µl (ρ = 1.32 g/ml, 4.52 mmol) dichloromethane, 1.18 ml (1.6 M in hexane, 1.18 mmol) *n*-butyllithium, 290 µl (ρ = 0.71 g/ml, 2.03 mmol) diisopropylamine and 411 mg (3.02 mmol) zinc chloride. To the α-chloro boronic ester solution were added 1.25 ml (3.0 M in diethyl ether, 3.77 mmol) ethylmagnesium bromide and the mixture was stirred for 20 h at room temperature, worked up and purified by flash chromatography (pentane, diethyl ether 98:2) to give **F** in 78 % yield (377 mg, 1.18 mmol) as a colorless oil, R_f = 0.16 (pentane); $[\alpha]_D^{20}$ = –51.4 (c =1.0, $CHCl_3$).

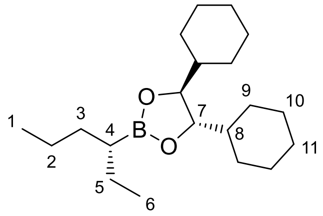

^{1}H-NMR (400 MHz, $CDCl_3$): δ = 0.84–0.93 (m, 6 H, 1-H, 6-H), 0.97 (m, 2 H, 9-H), 1.05 (m, 2 H, 10-H), 1.11–1.25 (m, 7 H, 4-H, 9-H', 10-H'), 1.25–1.36 (m, 5 H, 2-H, 3-H$_a$, 8-H), 1.37–1.48 (m, 3 H, 3-H$_b$, 5-H), 1.60 (m, 2 H, 9-H''), 1.68 (m, 2 H, 11-H), 1.72–1.84 (m, 6 H, 9-H''', 10-H'', 11-H'), 3.82 (m, 2 H, 4-H).
^{13}C-NMR (100 MHz, $CDCl_3$): δ = 13.7 (q, C-6), 14.4 (q, C-1), 22.3 (t, C-2), 24.2 (t, C-5), 25.9 (t, C-10), 26.0 (t, C-9), 26.5 (t, C-11), 27.5 (t, C-10'), 28.4 (t, C-9'), 33.4 (t, C-3), 43.1 (d, C-8), 83.2 (d, C-7).
The signal of C-4 could not be detected.
HRMS (CI) calcd for $C_{20}H_{38}O_2B$ $[M+H]^+$: 321.2959, found: 321.2981.

Ethyl (2*E*,5*S*)-5-[(4*S*,5*S*)-4,5-dicyclohexyl-1,3,2-dioxaborolan-2-yl]-2-methyloct-2-enoate (7a)

According to GP 1 245 mg (882 µmol) boronic ester **A**, 185 µl (ρ = 2.49 g/ml, 2.65 mmol) dibromomethane, 689 µl (1.6 M in hexanes, 1.10 mmol) *n*-butyllithium, 170 µl (ρ = 0.71 g/ml, 1.21 mmol) diisopropylamine and 240 mg (1.79 mmol) zinc chloride were converted to the crude α-bromo boronic ester which was directly used in the next step.

The freshly prepared α-bromo boronic ester, 153 µl (ρ = 0.923 g/ml, 1.10 mmol) ethyl tiglate, 145 µl (ρ = 0.71 g/ml, 1.01 mmol) diisopropylamine, 606 µl (1.6 M in hexanes, 970 µmol) *n*-butyllithium and 160 µl (ρ = 1.06 g/ml, 1.32 mmol) DMPU were reacted according to GP 2. Purification of the crude produt by flash chromatography (petroleum ether, ethyl acetate 95:5) gave **7a** in 61 % yield (224 mg, 535 µmol) as a colorless oil, R_f = 0.24 (petroleum ether, ethyl acetate 95:5); $[\alpha]_D^{20}$ = –37.4 (c =1.0, $CHCl_3$).

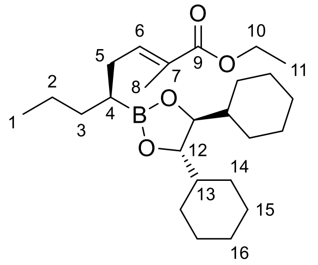

^{1}H-NMR (400 MHz, $CDCl_3$): δ = 0.85–0.99 (m, $^3J_{1,2}$ = 6.8 Hz, 5 H, 1-H, 15-H), 1.04 (m, 2 H, 14-H), 1.13–1.44 (m, $^3J_{11,10}$ = 7.1 Hz, 16 H, 2-H, 3-H_a, 4-H, 11-H, 13-H, 14-H', 15-H'), 1.46 (m, 1 H, 3-H_b), 1.57 (m, 2 H, 14-H''), 1.67 (m, 2 H, 16-H), 1.70–1.80 (m, 6 H, 14-H''', 15-H'', 26-H'), 1.82 (s, 3 H, 8-H), 2.26 (m, 2 H, 5-H), 3.82 (m, 2 H, 12-H), 4.17 (q, $^3J_{10,11}$ = 7.1 Hz, 2 H, 10-H), 6.78 (tq, $^3J_{6,5}$ = 7.5 Hz, $^4J_{6,8}$ = 1.2 Hz, 1 H, 6-H).
^{13}C-NMR (100 MHz, $CDCl_3$): δ = 12.4 (q, C-8), 14.3 (q, C-1, C-11), 22.2 (t, C-2), 22.7 (bs, C-4), 25.9 (t, C-15), 26.0 (t, C-14), 26.5 (t, C-16), 27.4 (t, C-15'), 28.4 (t, C-14'), 30.4 (t, C-5), 33.5 (t, C-3), 43.0 (d, C-13), 60.3 (t, C-10), 83.4 (d, C-12), 127.7 (s, C-7), 142.5 (d, C-6), 168.3 (s, C-9).
The α-Isomer could not be detected.
HRMS (CI) calcd for $C_{25}H_{44}O_4B$ $[M+H]^+$: 419.3327, found: 419.3329.

Ethyl (2*E*,5*S*)-5-[(4*S*,5*S*)-4,5-diisopropyl-1,3,2-dioxaborolan-2-yl]-2,7-dimethyloct-2-enoate (7b)

According to GP 1 187 mg (882 µmol) boronic ester **B**, 185 µl (ρ = 2.49 g/ml, 2.65 mmol) dibromomethane, 689 µl (1.6 M in hexanes, 1.10 mmol) *n*-butyllithium, 170 µl (ρ = 0.71 g/ml, 1.21 mmol) diisopropylamine and 240 mg (1.79 mmol) zinc chloride were converted to the crude α-bromo boronic ester which was directly used in the next step.

The freshly prepared α-bromo boronic ester, 153 µl (ρ = 0.923 g/ml, 1.10 mmol) ethyl tiglate, 145 µl (ρ = 0.71 g/ml, 1.01 mmol) diisopropylamine, 606 µl (1.6 M in hexanes, 970 µmol) *n*-butyllithium and 160 µl (ρ = 1.06 g/ml, 1.32 mmol) DMPU were reacted according to GP 2. Purification of the crude produt by flash chromatography (petroleum ether, ethyl acetate 95:5) gave **7b** (γ:α > 96:4) in 62 % yield (191 mg, 542 µmol) as a colorless oil, R_f = 0.25 (petroleum ether, ethyl acetate 95:5); $[\alpha]_D^{20}$ = –28.2 (c =1.0, $CHCl_3$).

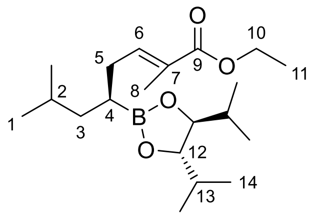

^{1}**H-NMR** (400 MHz, $CDCl_3$): δ = 0.83–0.94 (m, 18 H, 1-H, 14-H), 1.14–1.32 (m, $^{3}J_{11,10}$ = 7.1 Hz, 5 H, 3-H$_a$, 4-H, 11-H), 1.41 (ddd, $^{2}J_{3b,3a}$ = 12.6 Hz, $^{3}J_{3b,2}$ = 8.4 Hz, $^{3}J_{3b,2}$ = 6.0 Hz, 1 H, 3-H$_b$), 1.52–1.71 (m, 3 H, 2-H, 13-H), 1.82 (s, 3 H, 8-H), 2.25 (m, 2 H, 5-H), 3.79 (m, 2 H, 12-H), 4.16 (q, $^{3}J_{10,11}$ = 7.1 Hz, 2 H, 10-H), 6.78 (tq, $^{3}J_{6,5}$ = 7.5 Hz, $^{4}J_{6,8}$ = 1.3 Hz, 1 H, 6-H).
^{13}**C-NMR** (100 MHz, $CDCl_3$): δ = 12.4 (q, C-8), 14.3 (q, C-11), 16.8 (q, C-14), 17.9 (q, C-14'), 20.6 (bs, C-4), 22.5 (q, C-1), 22.9 (q, C-1'), 27.1 (d, C-2), 30.7 (t, C-5), 33.1 (d, C-13), 40.7 (t, C-3), 60.2 (t, C-10), 84.1 (d, C-12), 127.7 (s, C-7), 142.3 (d, C-6), 168.2 (s, C-9).
The α-Isomer could not be detected.
HRMS (CI) calcd for $C_{20}H_{38}O_4B$ $[M+H]^+$: 353.2858, found: 353.2848.

Ethyl (2*E*,5*S*)-9-[(*tert*-butyldimethylsilyl)oxy]-5-[(4*S*,5*S*)-4,5-dicyclohexyl-1,3,2-dioxaborolan-2-yl]-2-methylnon-2-enoate (7c)

According to GP 1 373 mg (882 µmol) boronic ester **C**, 185 µl (ρ = 2.49 g/ml, 2.65 mmol) dibromomethane, 689 µl (1.6 M in hexanes, 1.10 mmol) *n*-butyllithium, 170 µl (ρ = 0.71 g/ml, 1.21 mmol) diisopropylamine and 240 mg (1.79 mmol) zinc chloride were converted to the crude α-bromo boronic ester which was directly used in the next step.

The freshly prepared α-bromo boronic ester, 153 µl (ρ = 0.923 g/ml, 1.10 mmol) ethyl tiglate, 145 µl (ρ = 0.71 g/ml, 1.01 mmol) diisopropylamine, 606 µl (1.6 M in hexanes, 970 µmol) *n*-butyllithium and 160 µl (ρ = 1.06 g/ml, 1.32 mmol) DMPU were reacted according to GP 2. Purification of the crude produt by flash chromatography (petroleum ether, ethyl acetate 95:5) gave **7c** (γ:α > 96:4) in 69 % yield (342 mg, 608 µmol) as a colorless oil, R_f = 0.25 (petroleum ether, ethyl acetate 95:5); $[\alpha]_D^{20}$ = –32.0 (c =1.0, $CHCl_3$).

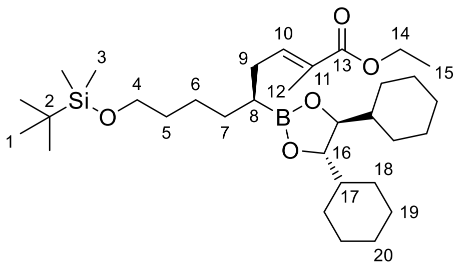

^{1}**H-NMR** (400 MHz, $CDCl_3$): δ = 0.04 (s, 6 H, 3-H), 0.89 (s, 9 H, 1-H), 0.94 (m, 2 H, 19-H), 1.04 (m, 2 H, 18-H), 1.12–1.44 (m, $^{3}J_{14,15}$ = 7.1 Hz, 15 H, 6-H, 7-H$_a$, 8-H, 15-H, 17-H, 18-H', 19-H'), 1.45–1.62 (m, 5 H, 5-H, 7-H$_b$, 18-H''), 1.67 (m, 2 H, 20-H), 1.70–1.79 (m, 6 H, 18-H''', 19-H'', 20-H'), 1.82 (s, 3 H, 11-H), 2.26 (m, 2 H, 9-H), 3.58 (t, $^{3}J_{4,5}$ = 6.6 Hz, 2 H, 4-H), 3.82 (m, 2 H, 16-H), 4.16 (q, $^{3}J_{13,14}$ = 7.1 Hz, 2 H, 14-H), 6.77 (tq, $^{3}J_{10,9}$ = 7.5 Hz, $^{4}J_{10,12}$ = 1.2 Hz, 1 H, 10-H).
^{13}**C-NMR** (100 MHz, $CDCl_3$): δ = –5.3 (q, C-3), 12.4 (q, C-12), 14.3 (q, C-15), 18.4 (s, C-2), 22.8 (bs, C-8), 25.4 (t, C-6), 25.9 (t, C-19), 26.0 (q, C-1), 26.0 (t, C-18), 26.5 (t, C-20), 27.4 (t, C-19'), 28.4 (t, C-18'), 30.3 (t, C-9), 31.0 (t, C-7), 33.1 (t, C-5), 43.0 (d, C-17), 60.3 (t, C-14), 63.3 (t, C-4), 83.4 (d, C-16), 127.7 (s, C-11), 142.4 (d, C-10), 168.2 (s, C-13).
The α-Isomer could not be detected.
HRMS (CI) calcd for $C_{32}H_{60}O_5BSi$ $[M+H]^+$: 563.4298, found: 563.4291.

Ethyl (2*E*,5*R*,6*S*,7*R*)-7-(benzyloxy)-5-[(4*S*,5*S*)-4,5-dicyclohexyl-1,3,2-dioxaborolan-2-yl]-2,6-dimethyldec-2-enoate (7d)

According to GP 1 363 mg (851 µmol) boronic ester **D** 178 µl (ρ = 2.49 g/ml, 2.55 mmol) dibromomethane, 665 µl (1.6 M in hexanes, 1.06 mmol) *n*-butyllithium, 164 µl (ρ = 0.71 g/ml, 1.15 mmol) diisopropylamine and 348 mg (2.55 mmol) zinc chloride were converted to the crude α-bromo boronic ester which was directly used in the next step.

The freshly prepared α-bromo boronic ester, 148 µl (ρ = 0.923 g/ml, 1.06 mmol) ethyl tiglate, 140 µl (ρ = 0.71 g/ml, 979 µmol) diisopropylamine, 585 µl (1.6 M in hexanes, 936 µmol) *n*-butyllithium and 154 µl (ρ = 1.06 g/ml, 1.28 mmol) DMPU were reacted according to GP 2. Purification of the crude produt by flash chromatography (petroleum ether, ethyl acetate 95:5) gave **7d** (γ:α > 96:4) in 68 % yield (329 mg, 581 µmol) as a colorless oil, R_f = 0.20 (petroleum ether, ethyl acetate 95:5); $[\alpha]_D^{20}$ = –21.1 (c =1.0, $CHCl_3$).

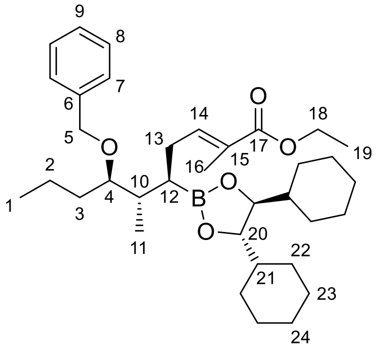

^{1}H-NMR (400 MHz, $CDCl_3$): δ = 0.85–1.06 (m, $^3J_{11,10}$ = 7.0 Hz, 10 H, 1-H, 11-H, 22-H, 23-H), 1.09–1.39 (m, $^3J_{19,18}$ = 7.1 Hz, 12 H, 2-H$_a$, 12-H, 21-H, 22-H‘, 23-H‘), 1.41–1.51 (m, 4 H, 2-H$_b$, 3-H, 12-H), 1.55 (m, 2 H, 22-H‘‘), 1.66 (m, 2 H, 24-H), 1.68–1.81 (m, 6 H, 22-H‘‘‘, 23-H‘‘, 24-H‘), 1.83 (s, 3 H, 16-H), 1.91 (ddq, $^3J_{10,4} \approx {}^3J_{10,11} \approx {}^3J_{10,12}$ = 6.5 Hz, 1 H, 10-H), 2.20 (ddd, $^2J_{13a,13b}$ = 14.8 Hz, $^3J_{13a,12} \approx {}^3J_{13a,14}$ = 6.6 Hz, 1 H, 13-H$_a$), 2.36 (ddd, $^2J_{13b,13a}$ = 14.8 Hz, $^3J_{13b,12} \approx {}^3J_{13b,14}$ = 8.3 Hz, 1 H, 13-H$_b$), 3.37 (dt, $^3J_{4,3} \approx {}^3J_{4,10}$ = 5.5 Hz, 1 H, 4-H), 3.75 (m, 2 H, 20-H), 4.17 (q, $^3J_{18,19}$ = 7.1 Hz, 2 H, 18-H), 4.46 (d, $^2J_{5a,5b}$ = 11.5 Hz, 1 H, 5-H$_a$), 4.57 (d, $^2J_{5b,5a}$ = 11.6 Hz, 1 H, 5-H$_b$), 6.77 (tq, $^3J_{14,13}$ = 7.5 Hz, $^4J_{14,16}$ = 1.3 Hz, 1 H, 14-H), 7.25 (m, 1 H, 9-H), 7.29–7.40 (m, 4 H, 7-H, 8-H).
^{13}C-NMR (100 MHz, $CDCl_3$): δ = 12.4 (q, C-16), 14.1 (q, C-11), 14.3 (q, C-19), 14.4 (q, C-1), 18.8 (t, C-2), 24.9 (bs, C-12), 25.9 (t, C-23), 26.0 (t, C-22), 26.4 (t, C-24), 27.8 (t, C-23’), 28.7 (t, C-22’), 29.1 (t, C-13), 32.4 (t, C-3), 37.4 (d, C-10), 43.0 (d, C-21), 60.3 (t, C-18), 71.4 (t, C-5), 81.9 (d, C-4), 83.5 (d, C-20), 127.2 (d, C-9), 127.7 (d, C-7), 127.7 (s, C-15), 128.2 (d, C-8), 139.3 (s, C-6), 142.5 (d, C-14), 168.2 (s, C-17).
The α-Isomer could not be detected.
HRMS (CI) calcd for $C_{35}H_{56}O_5B$ $[M+H]^+$: 567.4215, found: 567.4215.

Ethyl (2*E*,5*R*)-5-cyclohexyl-5-[(4*S*,5*S*)-4,5-dicyclohexyl-1,3,2-dioxaborolan-2-yl]-2-methylpent-2-enoate (7e)

According to GP 1 281 mg (882 µmol) boronic ester **E**, 185 µl (ρ = 2.49 g/ml, 2.65 mmol) dibromomethane, 689 µl (1.6 M in hexanes, 1.10 mmol) *n*-butyllithium, 170 µl (ρ = 0.71 g/ml, 1.21 mmol) diisopropylamine and 240 mg (1.79 mmol) zinc chloride were converted to the crude α-bromo boronic ester which was directly used in the next step.

The freshly prepared α-bromo boronic ester, 153 µl (ρ = 0.923 g/ml, 1.10 mmol) ethyl tiglate, 145 µl (ρ = 0.71 g/ml, 1.01 mmol) diisopropylamine, 606 µl (1.6 M in hexanes, 970 µmol) *n*-butyllithium and 160 µl (ρ = 1.06 g/ml, 1.32 mmol) DMPU were reacted according to GP 2. Purification of the crude produt by flash chromatography (petroleum ether, ethyl acetate 98:2)

gave **7e** (γ:α > 96:4) in 78 % yield (316 mg, 689 µmol) as a colorless oil, R_f = 0.15 (petroleum ether, ethyl acetate 98:2); $[\alpha]_D^{20}$ = –40.3 (c =1.0, $CHCl_3$).

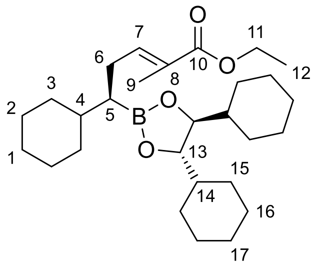

^{1}H-NMR (500 MHz, $CDCl_3$): δ = 0.91–1.23 (m, 14 H, 1-H, 2-H, 3-H, 15-H, 16-H), 1.25–1.31 (m, $^3J_{12,11}$ = 7.1 Hz, 5 H, 12-H, 14-H), 1.40 (m, 1 H, 4-H), 1.58 (m, 2 H, 15-H'), 1.60–1.80 (m, 14 H, 1-H', 2-H', 3-H', 15-H'', 16-H', 17-H), 1.82 (q, $^4J_{9,7}$ = 1.3 Hz, 3 H, 9-H), 2.27 (m, 2 H, 6-H), 3.80 (m, 2 H, 13-H), 4.16 (q, $^3J_{11,12}$ = 7.3 Hz, 2 H, 11-H), 6.76 (tq, $^3J_{7,6}$ = 7.6 Hz, $^4J_{7,9}$ = 1.6 Hz, 1 H, 7-H).
^{13}C-NMR (125 MHz, $CDCl_3$): δ = 25.9 (t, C-16), 26.0 (t, C-15), 26.5 (t, C-17), 26.7 (t, C-2), 26.7 (t, C-2'), 27.6 (t, C-16'), 28.0 (t, C-1), 28.5 (t, C-15'), 30.1 (bs, C-5), 32.1 (t, C-3), 32.7 (t, C-3'), 39.5 (d, C-4), 43.0 (d, C-14), 60.3 (t, C-11), 83.6 (d, C-13), 127.4 (s, C-8), 143.0 (d, C-7), 168.3 (s, C-10).
The α-Isomer could not be detected.
HRMS (CI) calcd for $C_{28}H_{48}O_4B$ $[M+H]^+$: 459.3640, found: 459.3648.

Ethyl (2*E*,5*R*,6*R*)-5-((4S,5S)-4,5-dicyclohexyl-1,3,2-dioxaborolan-2-yl)-6-ethyl-2-methylnon-2-enoate (7f)

According to GP 1 283 mg (882 µmol) boronic ester **F**, 185 µl (ρ = 2.49 g/ml, 2.65 mmol) dibromomethane, 689 µl (1.6 M in hexanes, 1.10 mmol) *n*-butyllithium, 170 µl (ρ = 0.71 g/ml, 1.21 mmol) diisopropylamine and 240 mg (1.79 mmol) zinc chloride were converted to the crude α-bromo boronic ester which was directly used in the next step.

The freshly prepared α-bromo boronic ester, 153 µl (ρ = 0.923 g/ml, 1.10 mmol) ethyl tiglate, 145 µl (ρ = 0.71 g/ml, 1.01 mmol) diisopropylamine, 606 µl (1.6 M in hexanes, 970 µmol) *n*-butyllithium and 160 µl (ρ = 1.06 g/ml, 1.32 mmol) DMPU were reacted according to GP 2. Purification of the crude produt by flash chromatography (petroleum ether, diethyl ether 95:5) gave **7f** (γ:α > 96:4) in 61 % yield (249 mg, 541 µmol) as a colorless oil, R_f = 0.21 (petroleum ether, diethyl ether 95:5); $[\alpha]_D^{20}$ = –32.6 (c =1.0, $CHCl_3$).

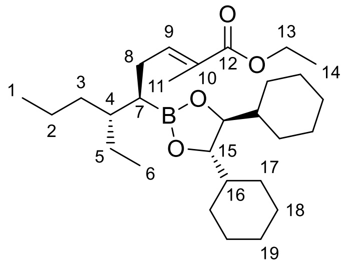

^{1}H-NMR (400 MHz, $CDCl_3$): δ = 0.82–0.90 (m, 6 H, 1-H, 6-H), 0.96 (m, 2 H, 17-H), 1.05 (m, 2 H, 18-H), 1.12–1.23 (m, 6 H, 17-H', 18-H'), 1.24–1.34 (m, 11 H, 2-H, 3-H, 5-H_a, 16-H), 1.34–1.45 (m, 2 H, 4-H, 5-H_b), 1.56 (m, 2 H, 17-H''), 1.67 (m, 2 H, 19-H), 1.70–1.80 (m, 6 H, 17-H''', 18-H'', 19-H'), 1.83 (s, 3 H, 11-H), 2.18 (ddd, $^2J_{8a,8b}$ = 13.6 Hz, $^3J_{8a,7}$ ≈ $^3J_{8a,9}$ = 6.6 Hz, 1 H, 8-H_a), 2.29 (ddd, $^2J_{8b,8a}$ = 14.4 Hz, $^3J_{8b,7}$ ≈ $^3J_{8b,9}$ = 9.0 Hz, 1 H, 8-H_b), 3.79 (m, 2 H, 15-H), 4.16 (q, $^3J_{13,14}$ = 7.1 Hz, 2 H, 13-H), 6.77 (tq, $^3J_{9,8}$ = 7.6 Hz, $^4J_{9,11}$ = 1.2 Hz, 1 H, 9-H).

^{13}C-NMR (100 MHz, $CDCl_3$): δ = 12.2 (q, C-6), 12.3 (q, C-11), 14.3 (q, C-14), 14.5 (q, C-1), 20.2 (t, C-2), 25.4 (t, C-5), 25.9 (t, C-18), 26.0 (t, C-17), 26.5 (t, C-19), 27.5 (t, C-8), 27.6 (t, C-18'), 28.5 (t, C-17'), 34.6 (t, C-3), 41.0 (d, C-4), 43.0 (d, C-16), 60.2 (t, C-13), 83.6 (d, C-15), 127.6 (s, C-10), 143.3 (d, C-9), 168.3 (s, C-12).
The signal of C-7 could not be detected.
The α-Isomer could not be detected.
HRMS (CI) calcd for $C_{28}H_{50}O_4B$ $[M+H]^+$: 461.3797, found: 461.3787.

Ethyl (2*E*,5*R*,6*S*,7*R*,8*S*)-7-(benzyloxy)-5-[(4*S*,5*S*)-4,5-dicyclohexyl-1,3,2-dioxaborolan-2-yl]-2,6,8-trimethyldec-2-enoate (9)

(4*S*,5*S*)-2-[(2*R*,3*S*,4*S*)-3-(benzyloxy)-4-methylhexan-2-yl]-4,5-dicyclohexyl-1,3,2-dioxaborolane **8** was prepared as described previously.[13]

According to GP 1 360 mg (817 µmol) boronic ester **8**, 171 µl (ρ = 2.49 g/ml, 2.45 mmol) dibromomethane, 639 µl (1.6 M in hexanes, 1.02 mmol) *n*-butyllithium, 157 µl (ρ = 0.71 g/ml, 1.10 mmol) diisopropylamine and 334 mg (2.45 mmol) zinc chloride were converted to the crude α-bromo boronic ester which was directly used in the next step.

The freshly prepared α-bromo boronic ester, 142 µl (ρ = 0.923 g/ml, 1.02 mmol) ethyl tiglate, 134 µl (ρ = 0.71 g/ml, 940 µmol) diisopropylamine, 562 µl (1.6 M in hexanes, 899 µmol) *n*-butyllithium and 148 µl (ρ = 1.06 g/ml, 1.23 mmol) DMPU were reacted according to GP 2. Purification of the crude produt by flash chromatography (petroleum ether, ethyl acetate 97:3) gave **9** (γ:α > 96:4) in 76 % yield (362 mg, 623 µmol) as a colorless oil, R_f = 0.25 (petroleum ether, ethyl acetate 95:5); $[\alpha]_D^{20}$ = –38.6 (c =1.0, $CHCl_3$).

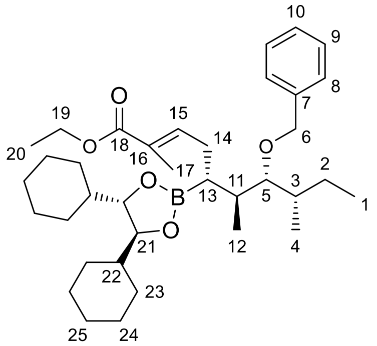

^{1}H-NMR (400 MHz, $CDCl_3$): δ = 0.85–1.05 (m, 13 H, 1-H, 4-H, 12-H, 23-H, 24-H), 1.09–1.39 (m, $^3J_{20,19}$ = 7.2 Hz, 12 H, 2-H_a, 22-H, 23-H', 24-H'), 1.41–1.59 (m, 3 H, 2-H_b, 23-H''), 1.60–1.81 (m, 10 H, 3-H, 13-H, 23-H''', 24-H'', 25-H), 1.81–1.89 (m, 4 H, 17-H, 11-H), 2.15 (ddd, $^2J_{14a,14b}$ = 14.5 Hz, $^3J_{14a,13}$ ≈ $^3J_{14a,15}$ = 7.2 Hz, 1 H, 14-H_a), 2.42 (ddd, $^2J_{14b,14a}$ = 15.3 Hz, $^3J_{14b,13}$ ≈ $^3J_{14b,15}$ = 7.6 Hz, 1 H, 14-H_b), 3.28 (dd, $^3J_{5,3}$ = 7.9 Hz, $^3J_{5,11}$ = 3.2 Hz, 1 H, 5-H), 3.75 (m, 2 H, 21-H), 4.17 (q, $^3J_{19,20}$ = 7.1 Hz, 2 H, 19-H), 4.58 (d, $^2J_{6a,6b}$ = 11.7 Hz, 1 H, 6-H_a), 4.74 (d, $^2J_{6b,6a}$ = 11.6 Hz, 1 H, 6-H_b), 6.79 (tq, $^3J_{15,14}$ = 7.5 Hz, $^4J_{15,17}$ = 1.2 Hz, 1 H, 15-H), 7.24 (m, 1 H, 10-H), 7.29–7.40 (m, 4 H, 8-H, 9-H).
^{13}C-NMR (100 MHz, $CDCl_3$): δ = 12.2 (q, C-1), 12.4 (q, C-17), 13.3 (q, C-4), 14.3 (q, C-20), 14.9 (q, C-12), 25.9 (t, C-24), 26.0 (t, C-23), 26.5 (t, C-25), 27.7 (t, C-2), 27.8 (t, C-24'), 28.7 (t, C-23'), 29.8 (t, C-14), 37.3 (d, C-3), 38.7 (d, C-11), 43.0 (d, C-22), 60.2 (t, C-19), 74.7 (t, C-6), 83.5 (d, C-21), 86.7 (d, C-5), 127.0 (d, C-10), 127.0 (d, C-8), 127.5 (s, C-16), 128.1 (d, C-9), 139.8 (s, C-7), 143.1 (d, C-15), 168.3 (s, C-18).
The signal of C-13 could not be detected.
The α-Isomer could not be detected.
HRMS (CI) calcd for $C_{36}H_{58}O_5B$ $[M+H]^+$: 581.4372, found: 581.4364.

---

[13] J. Gorges, U. Kazmaier, *Org. Lett.* **2018**, *20*, 2033–2036.

Ethyl (2*E*,5R,6S,7R,8S)-7-(benzyloxy)-5-hydroxy-2,6,8-trimethyldec-2-enoate (10)

139 mg (239 µmol) boronic ester **9**, 111 µl (33 % in water, ρ = 1.11 g/ml, 1.20 mmol) hydrogen peroxide and 127 mg (1.20 mmol) sodium carbonate were reacted according to GP 3. After 5 h, the reaction was worked up and the crude product was purified by flash chromatography (petroleum ether, ethyl acetate 9:1, 3:1) to give **10** in 71 % yield (62 mg, 171 µmol) as a colorless oil, R_f = 0.34 (petroleum ether, ethyl acetate 8:2); $[\alpha]_D^{20}$ = +4.7 (c = 1.0, $CHCl_3$), and (*S*,*S*)-DICHED in 79 % yield (43 mg, 190 µmol) as a colorless solid, R_f = 0.19 (petroleum ether, ethyl acetate 8:2); m. p. 142 °C; $[\alpha]_D^{20}$ = +2.7 (c =1.0, $CHCl_3$).

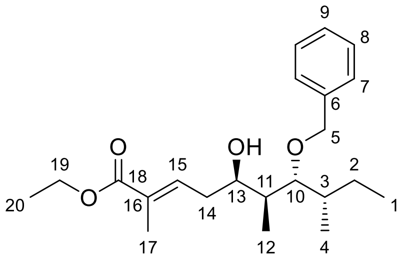

¹H-NMR (400 MHz, $CDCl_3$): δ = 0.85 (t, $^3J_{1,2}$ = 7.4 Hz, 3 H, 1-H), 0.96 (d, $^3J_{4,3}$ = 6.8 Hz, 3 H, 4-H), 0.98 (d, $^3J_{12,11}$ = 7.1 Hz, 3 H, 12-H), 1.12 (m, 1 H, 2-H_a), 1.21 (t, $^3J_{20,19}$ = 7.1 Hz, 3 H, 20-H), 1.43 (m, 1 H, 2-H_b), 1.65–1.76 (m, 2 H, 3-H, 11-H), 1.78 (s, 3 H, 17-H), 2.16 (ddd, $^2J_{14a,14b}$ = 14.1 Hz, $^3J_{14a,15} \approx {}^3J_{14a,13}$ = 7.5 Hz, 1 H, 14-H_a), 2.36 (ddd, $^2J_{14b,14a}$ = 15.0 Hz, $^3J_{14a,15} \approx {}^3J_{14a,13}$ = 7.5 Hz, 1 H, 14-H_b), 3.13 (d, $^3J_{OH,13}$ = 2.2 Hz, 1 H, OH), 3.23 (dd, $^3J_{10,11}$ = 6.7 Hz, $^3J_{10,3}$ = 4.0 Hz, 1 H, 10-H), 4.05 (m, 1 H, 13-H), 4.11 (q, $^3J_{19,20}$ = 7.1 Hz, 2 H, 19-H), 4.51 (d, $^2J_{5a,5b}$ = 11.0 Hz, 1 H, 5-H_a), 4.60 (d, $^2J_{5b,5a}$ = 10.9 Hz, 1 H, 5-H_b), 6.71 (tq, $^3J_{16,14}$ = 7.3 Hz, $^4J_{15,17}$ = 1.3 Hz, 1 H, 16-H), 7.17–7.32 (m, 5 H, 7-H, 8-H, 9-H).
¹³C-NMR (100 MHz, $CDCl_3$): δ = 11.5 (q, C-1), 11.7 (q, C-12), 12.6 (q, C-17), 14.3 (q, C-20), 15.0 (q, C-4), 26.5 (t, C-2), 34.1 (t, C-14), 37.7 (d, C-3), 38.1 (d, C-11), 60.4 (t, C-19), 70.2 (d, C-13), 75.7 (t, C-5), 89.1 (d, C-10), 127.6 (d, C-7), 127.8 (d, C-9), 128.5 (d, C-8), 129.3 (s, C-16), 138.1 (s, C-6), 138.6 (d, C-15), 168.0 (s, C-18).
HRMS (CI) calcd for $C_{22}H_{35}O_4$ $[M+H]^+$: 363.2530, found: 363.2517.

Copies of the NMR spectra and HPLC chromatograms

(4*S*,5*S*)-2-[(*R*)-1-Bromo-3-phenylpropyl]-4,5-dicyclohexyl-1,3,2-dioxaborolane (2, crude product**)**

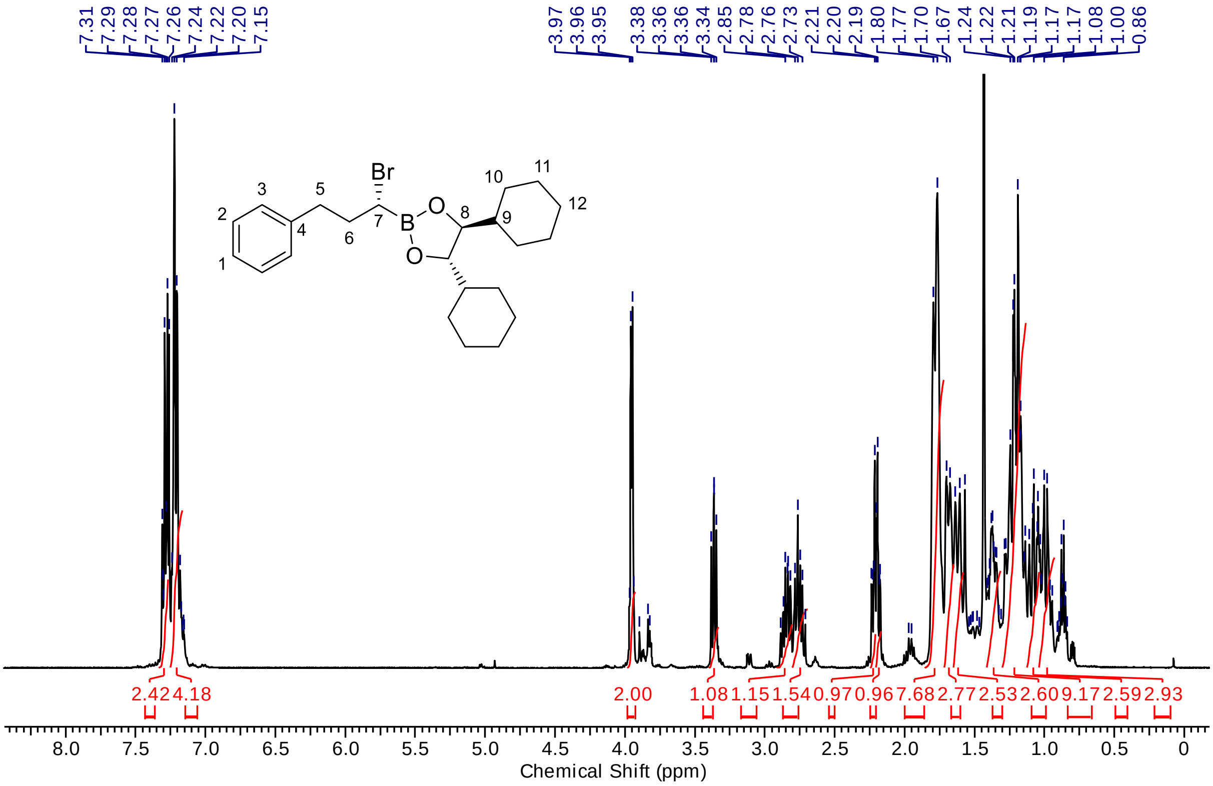

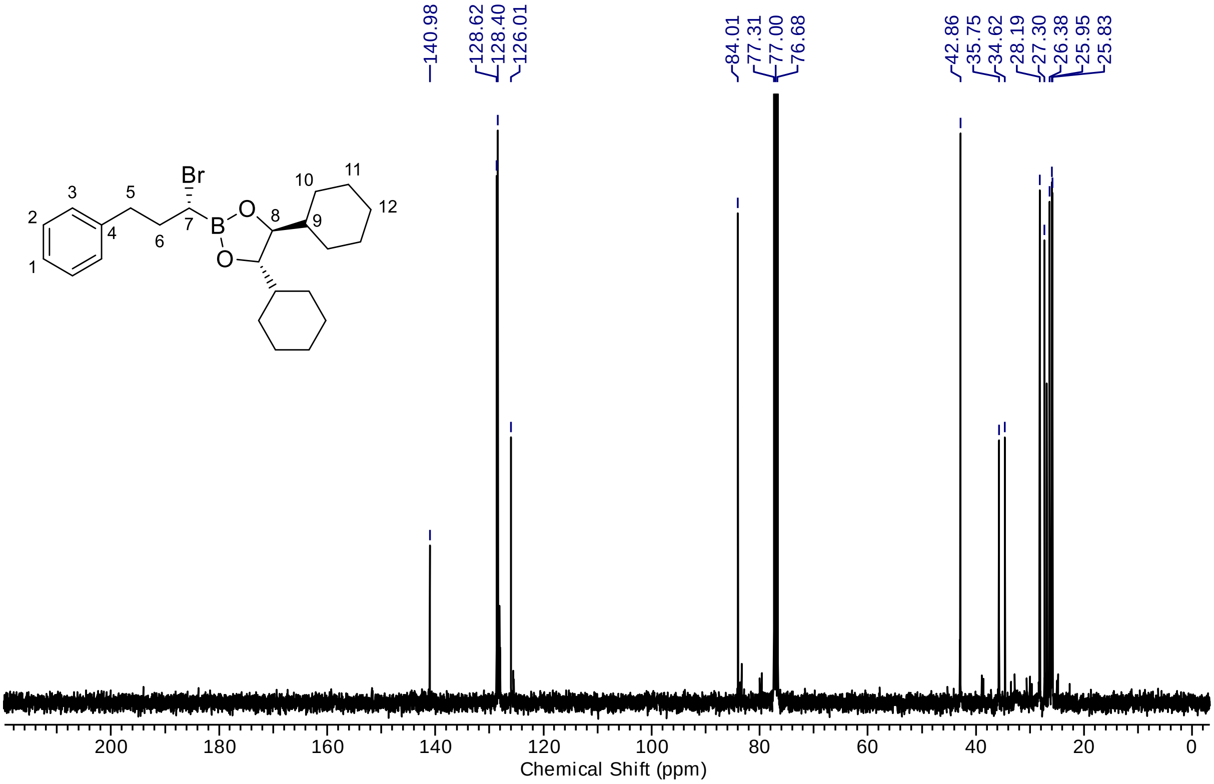

***tert*-Butyl (*S*,*E*)-5-[(4*S*,5*S*)-4,5-dicyclohexyl-1,3,2-dioxaborolan-2-yl]-7-phenylhept-2-enoate (3a**, contains α isomers **3a’** as impurity**)**

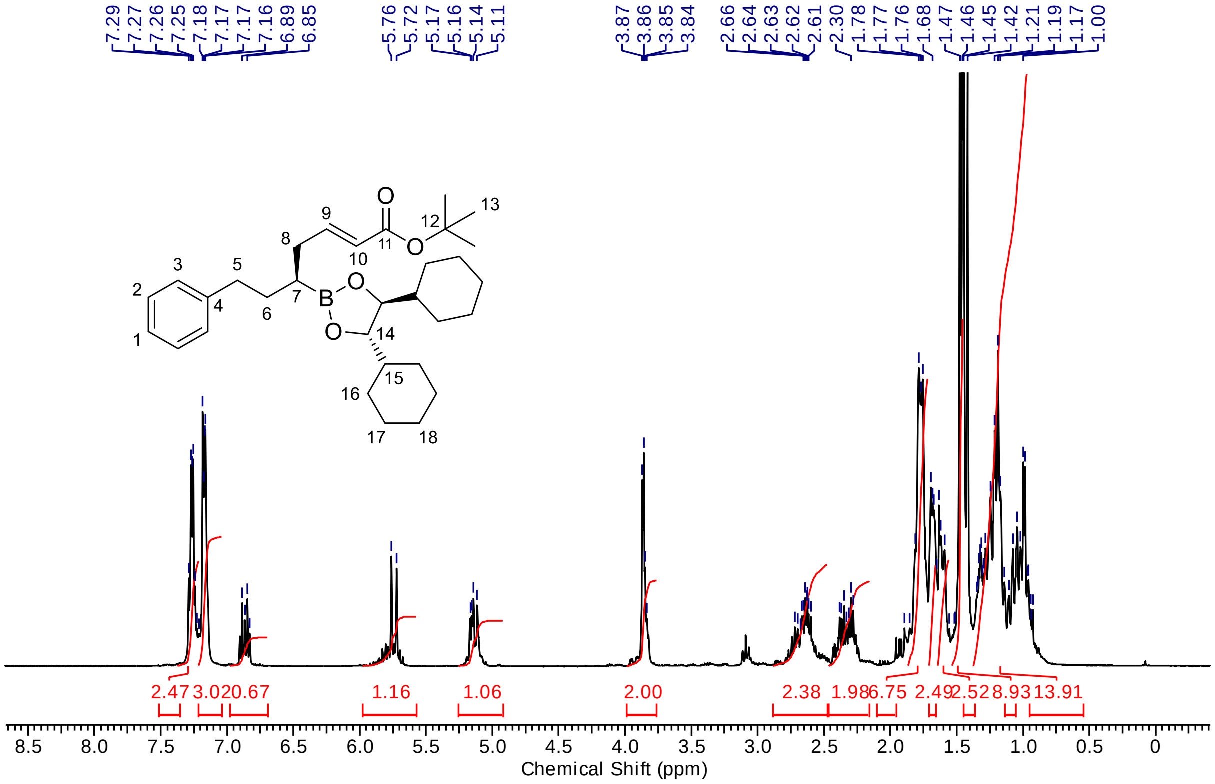

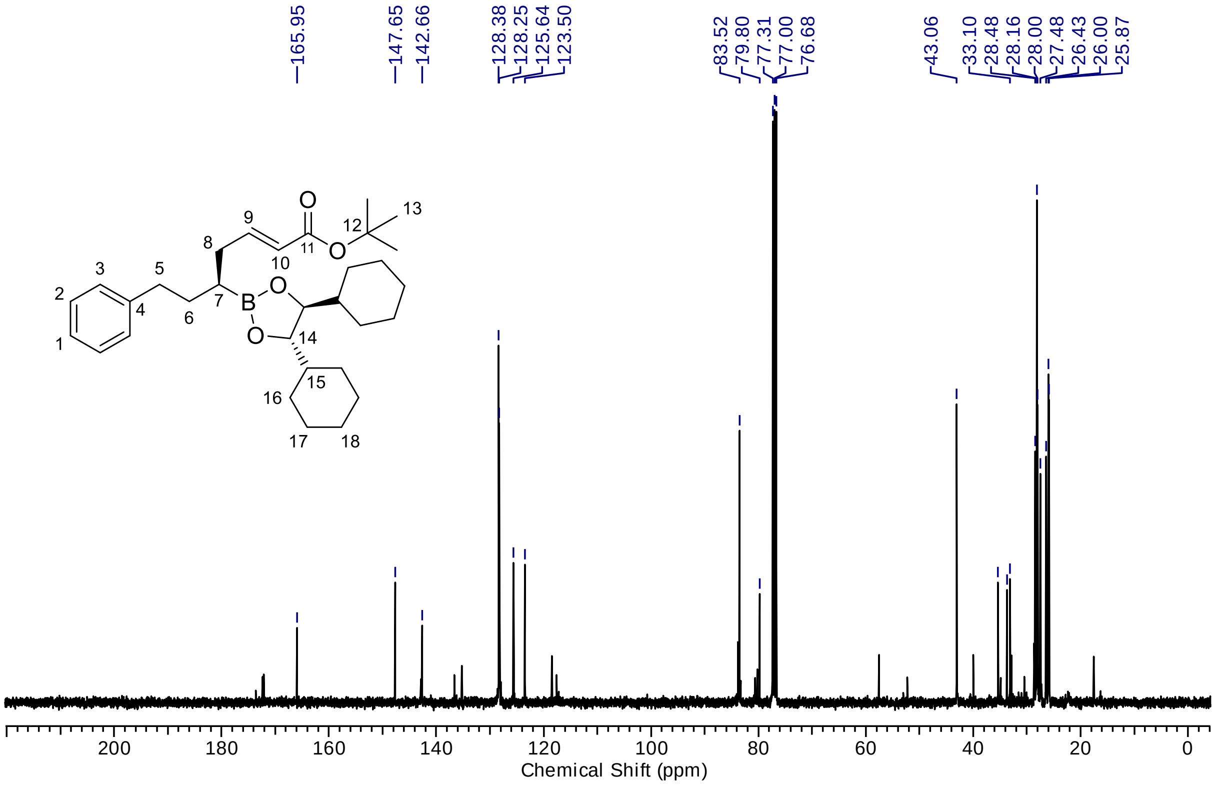

***tert*-Butyl (*S*,*E*)-5-hydroxy-7-phenylhept-2-enoate**

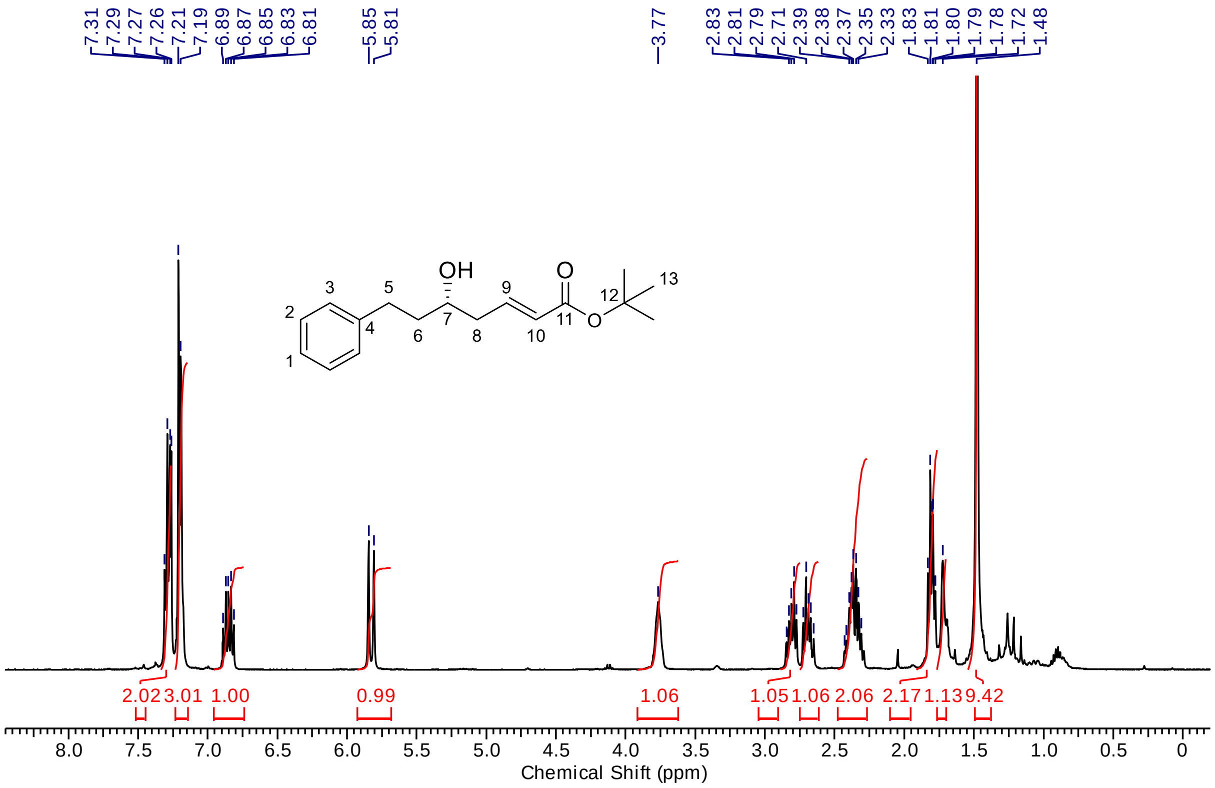

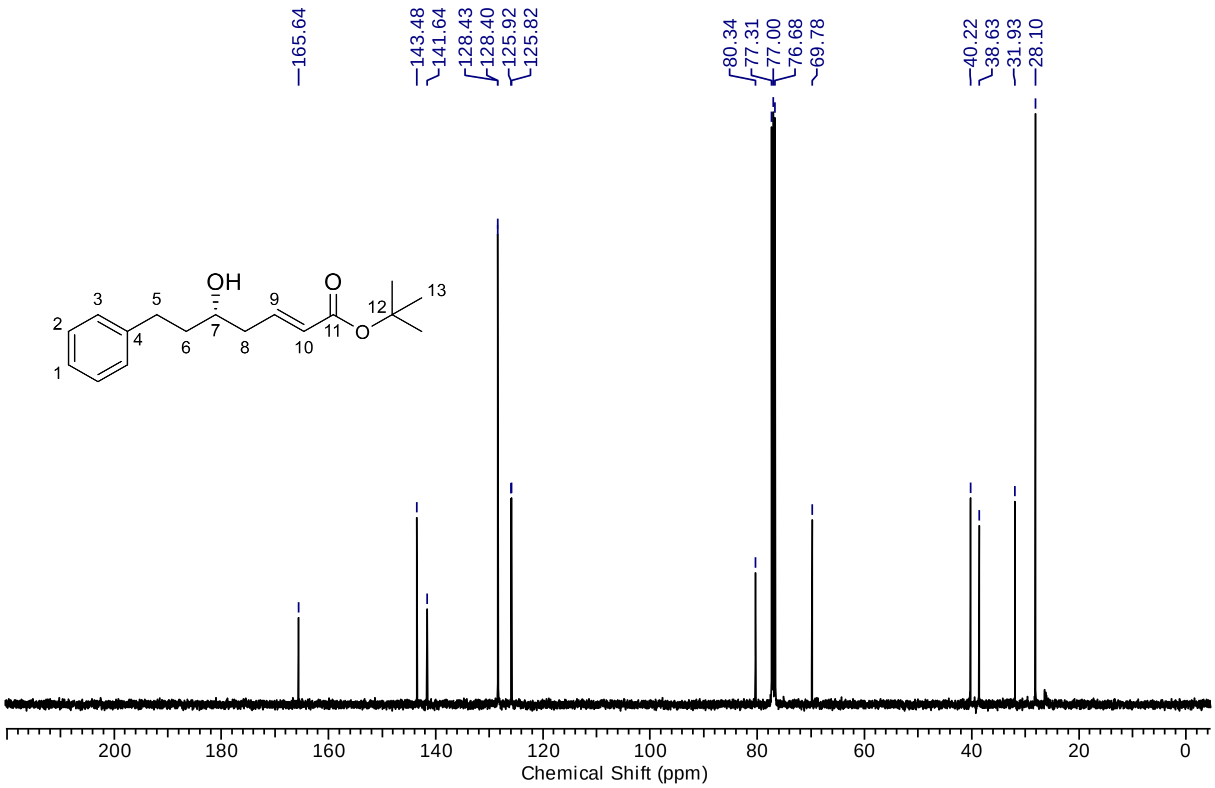

Ethyl (*S*,*E*)-5-[(4*S*,5*S*)-4,5-dicyclohexyl-1,3,2-dioxaborolan-2-yl]-7-phenylhept-2-enoate (3b, contains α isomers **3b'** as impurity**)**

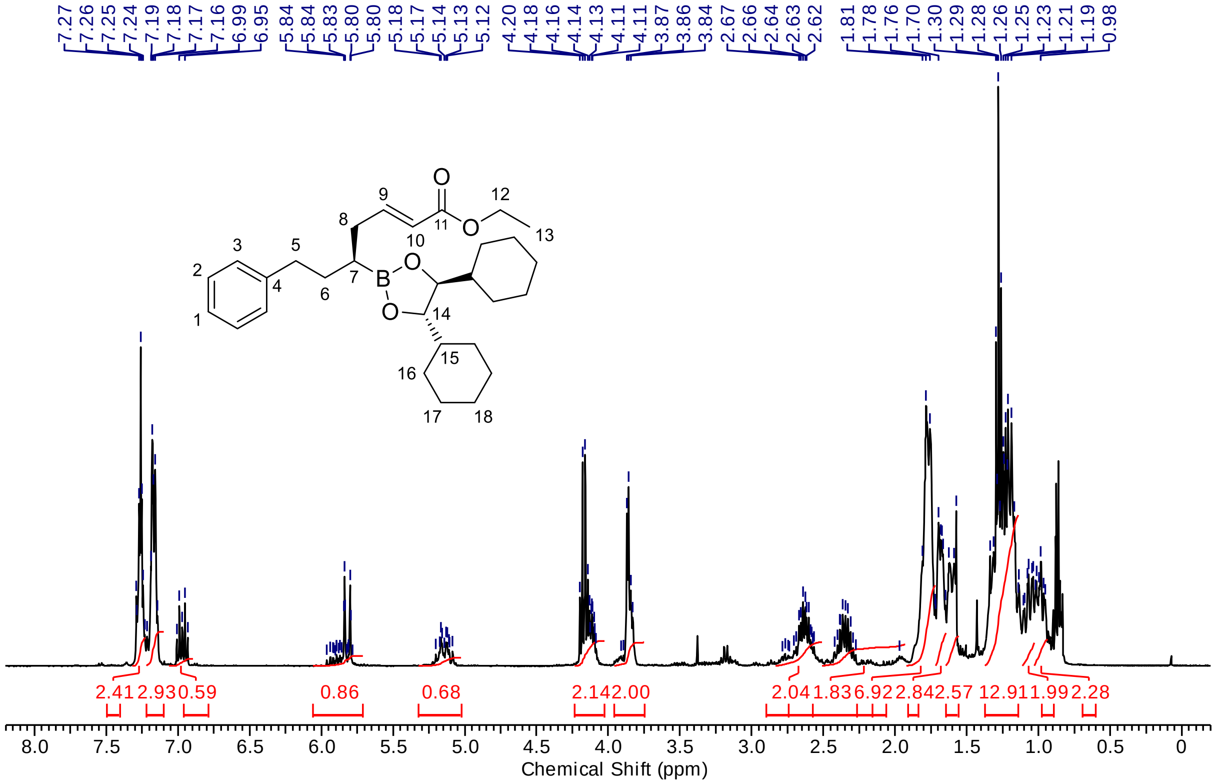

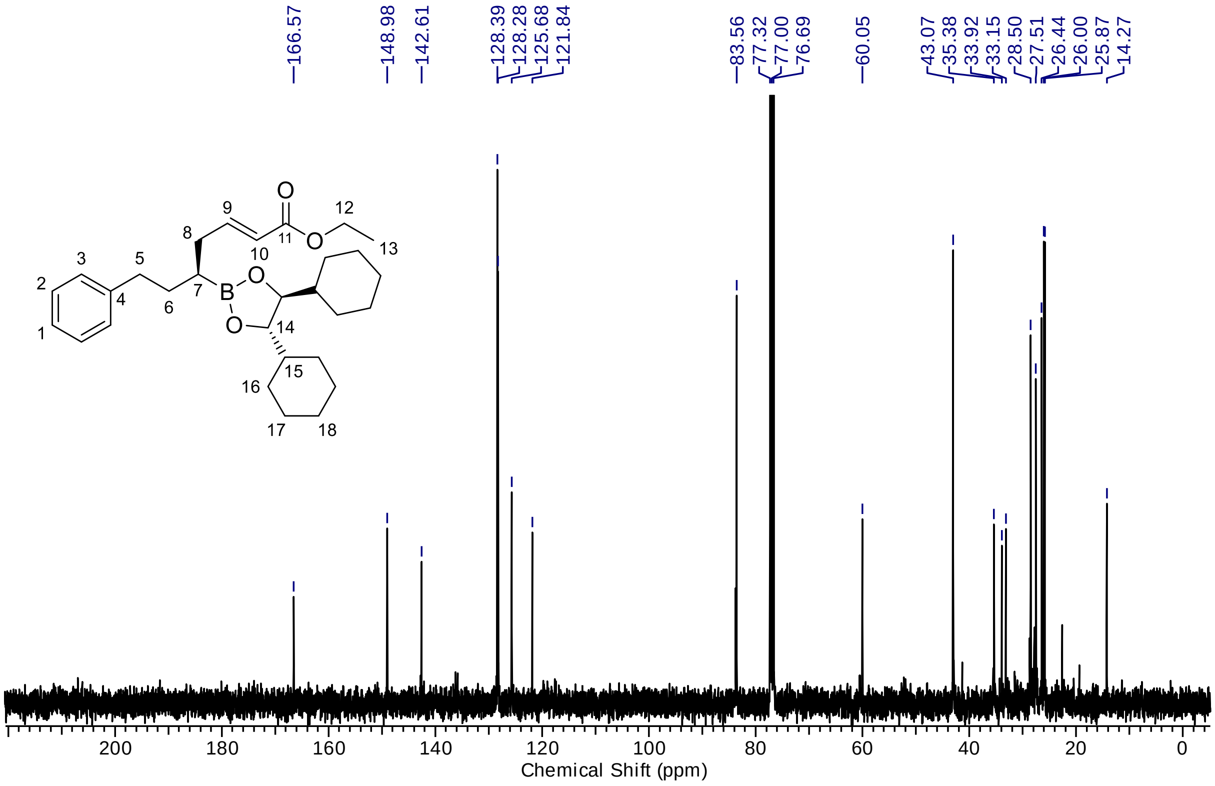

tert-Butyl (_S_,_E_)-5-[(4_S_,5_S_)-4,5-dicyclohexyl-1,3,2-dioxaborolan-2-yl]-2-methyl-7-phenylhept-2-enoate (5a)

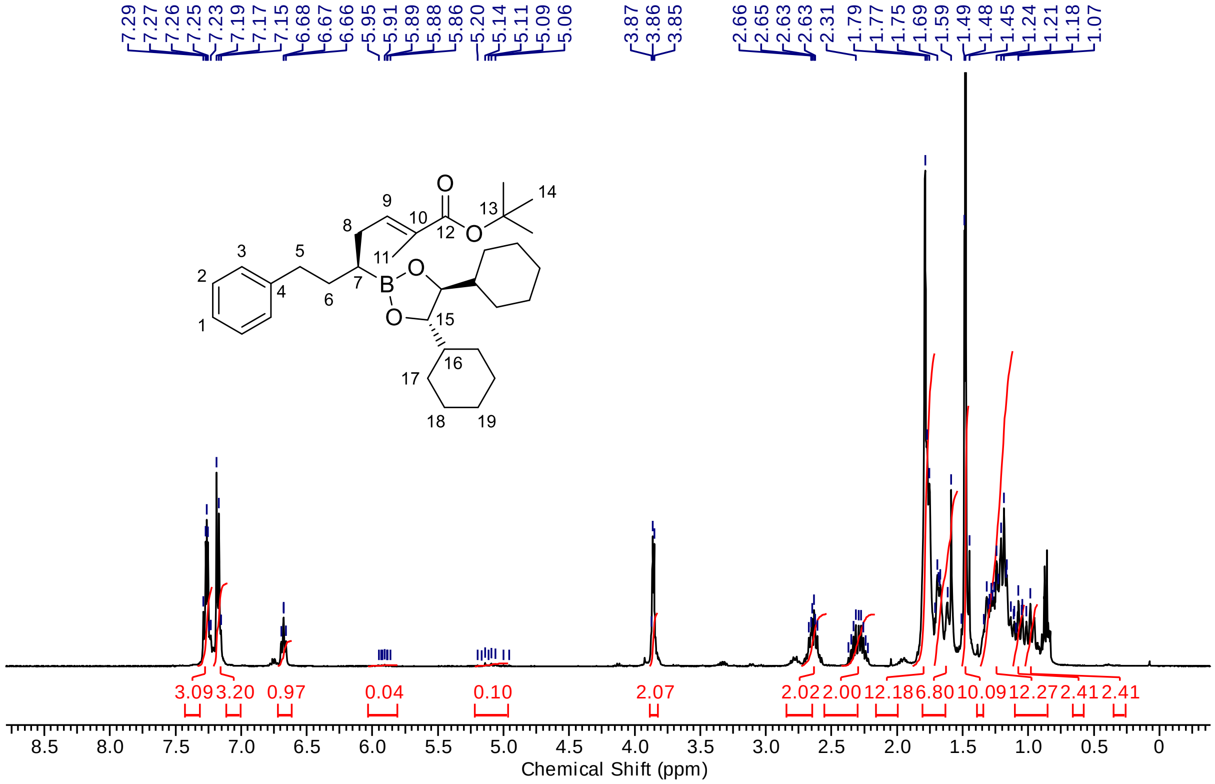

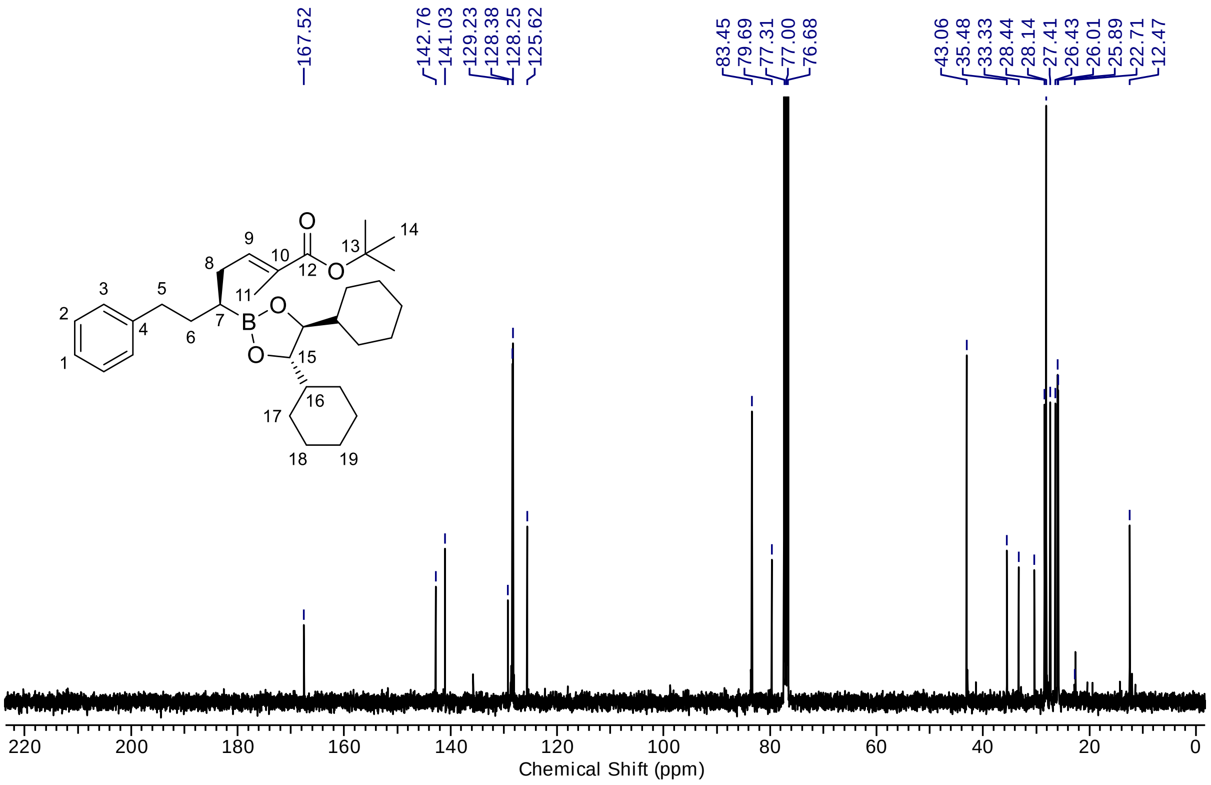

Ethyl (*S*,*E*)-5-[(4*S*,5*S*)-4,5-dicyclohexyl-1,3,2-dioxaborolan-2-yl]-2-methyl-7-phenylhept-2-enoate (5b)

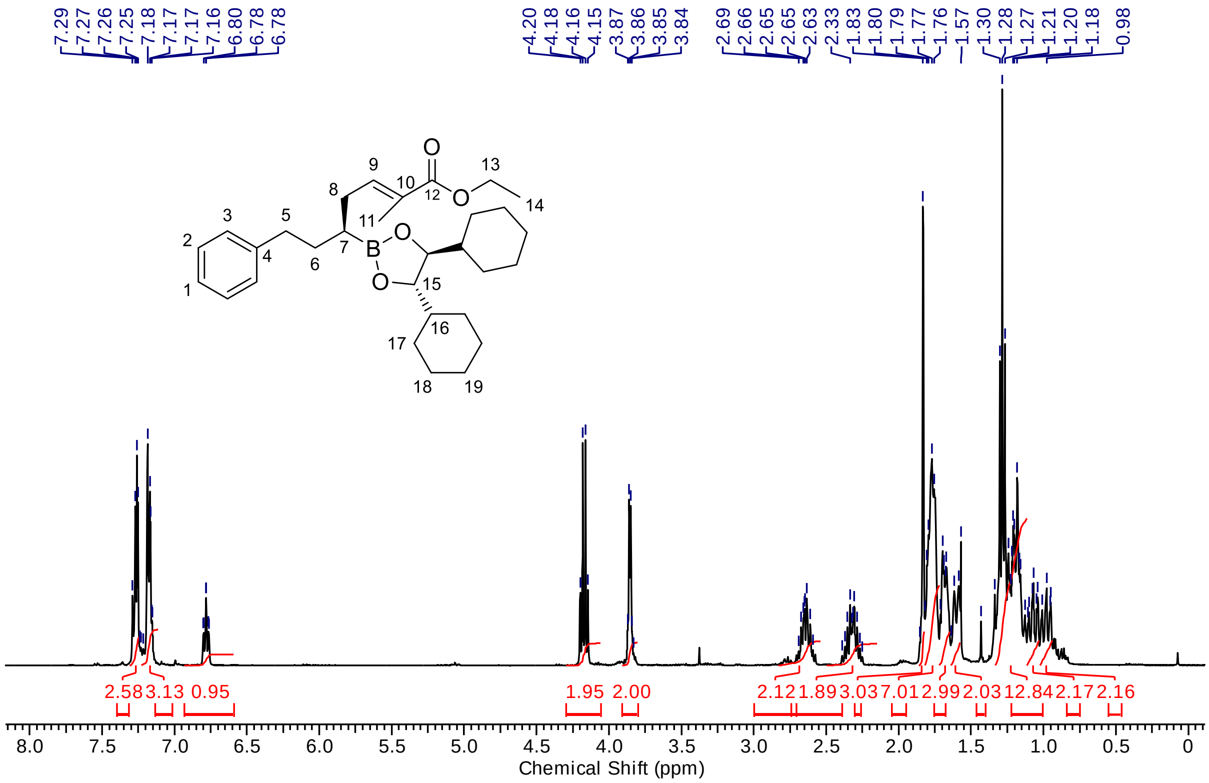

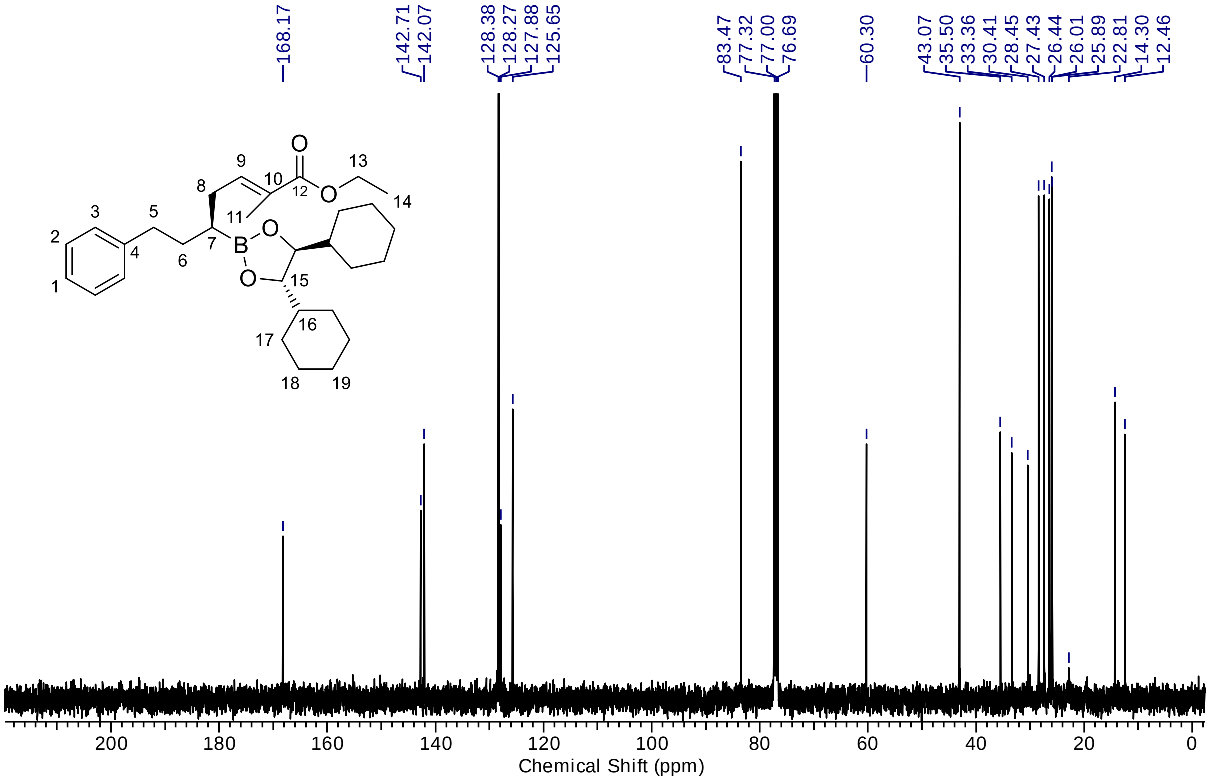

Methyl (*S*,*E*)-5-[(4*S*,5*S*)-4,5-dicyclohexyl-1,3,2-dioxaborolan-2-yl]-2-methyl-7-phenylhept-2-enoate (5c)

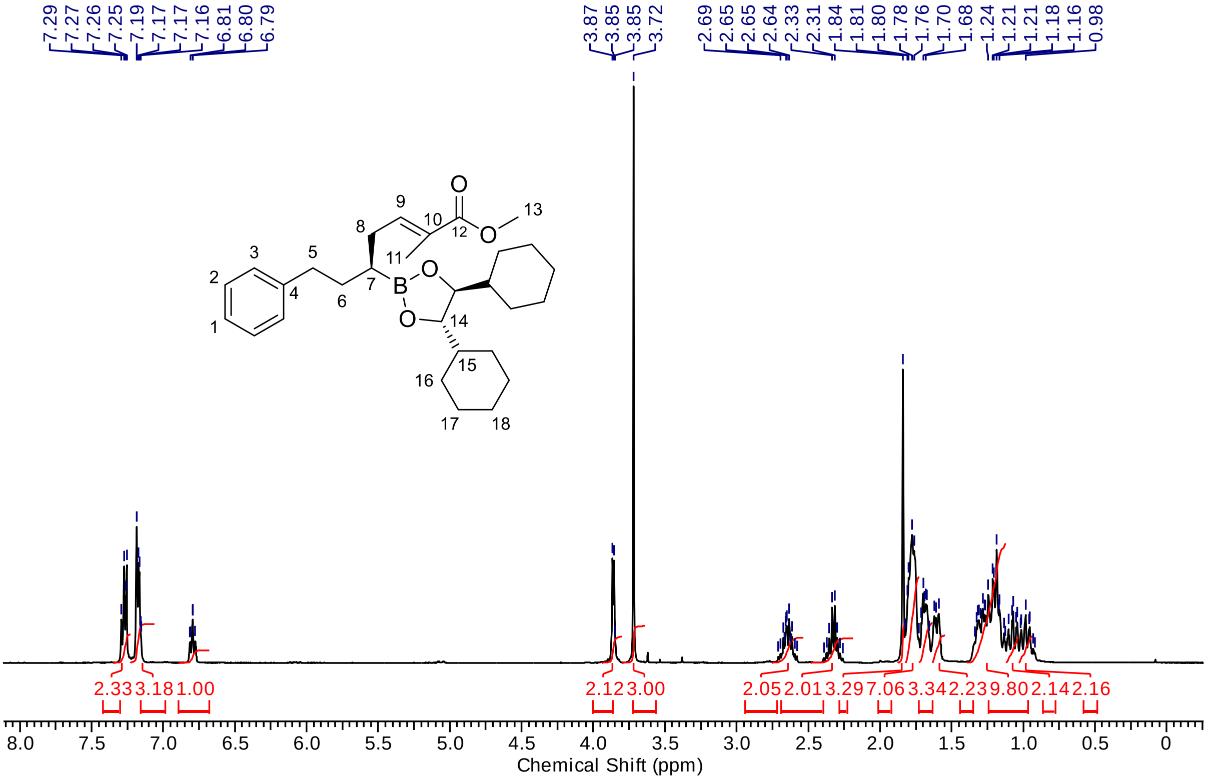

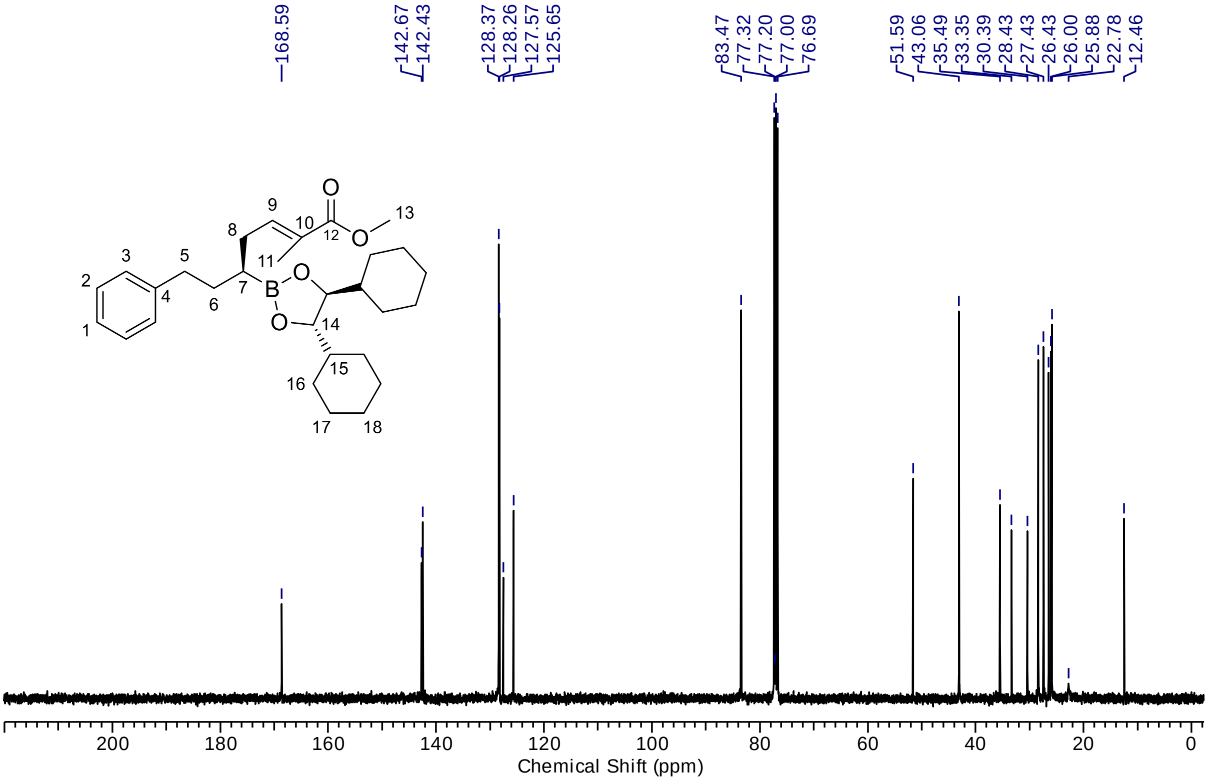

Ethyl (*S*,*E*)-5-[(4*S*,5*S*)-4,5-Dicyclohexyl-1,3,2-dioxaborolan-2-yl]-2,3-dimethyl-7-phenylhept-2-enoate (5d)

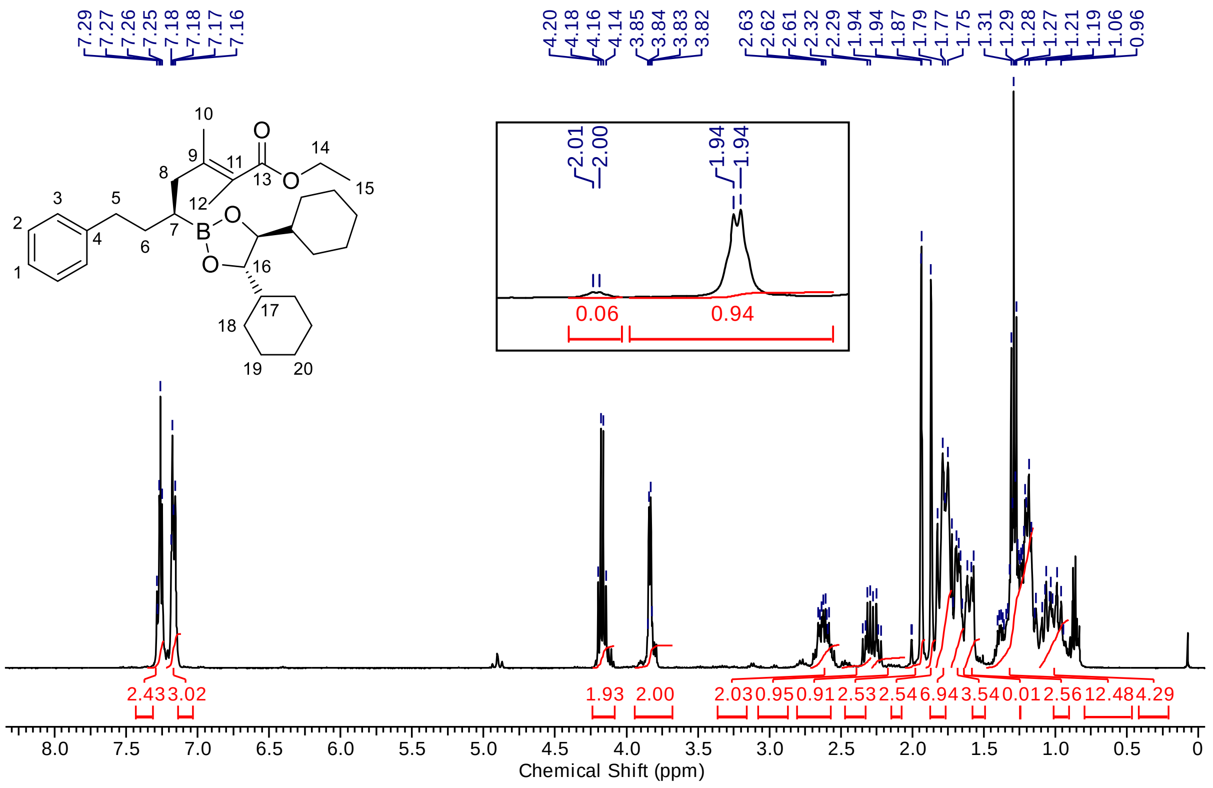

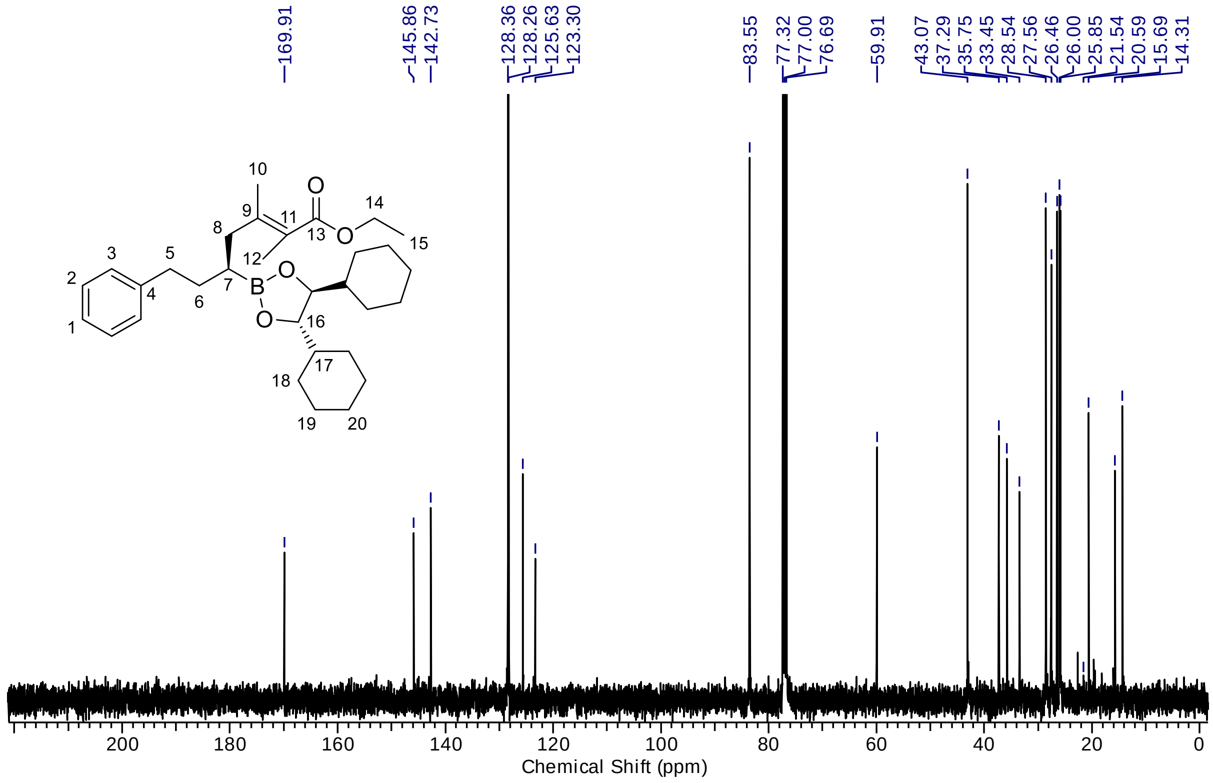

***tert*-Butyl (*S*,*E*)-5-hydroxy-2-methyl-7-phenylhept-2-enoate (6a)**

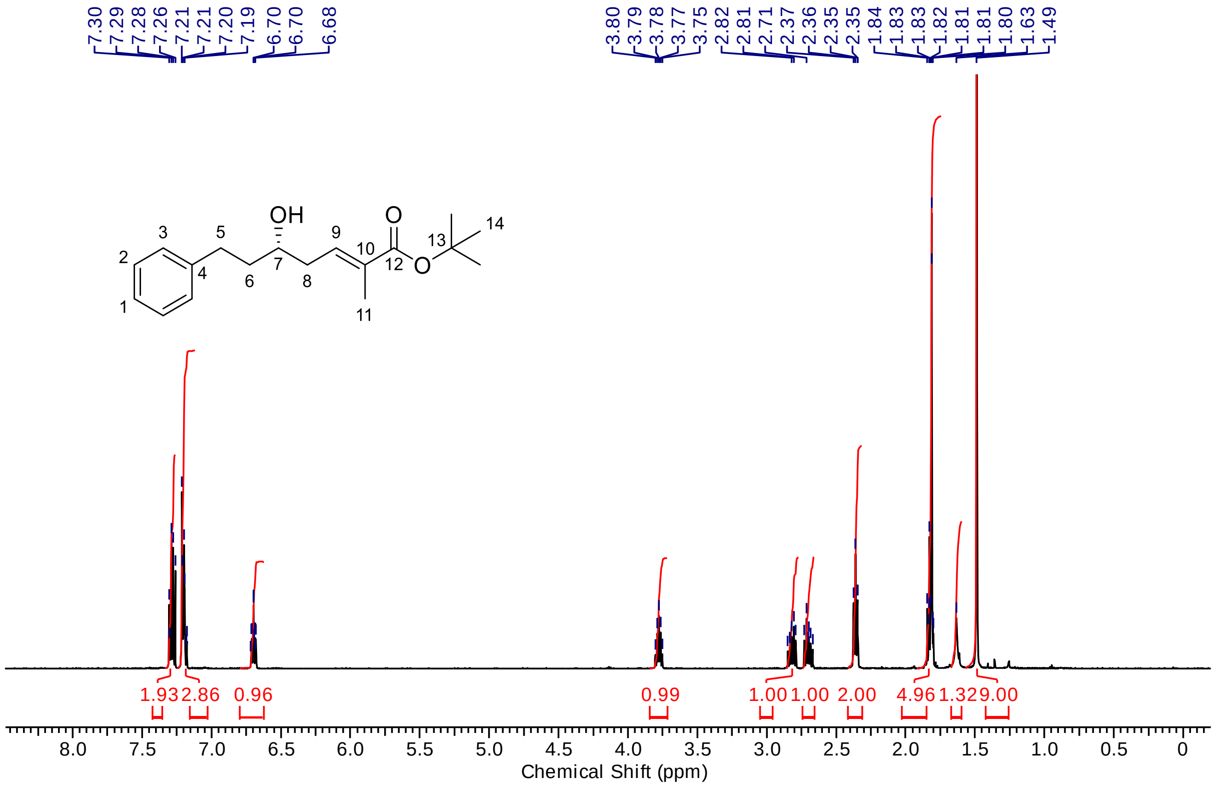

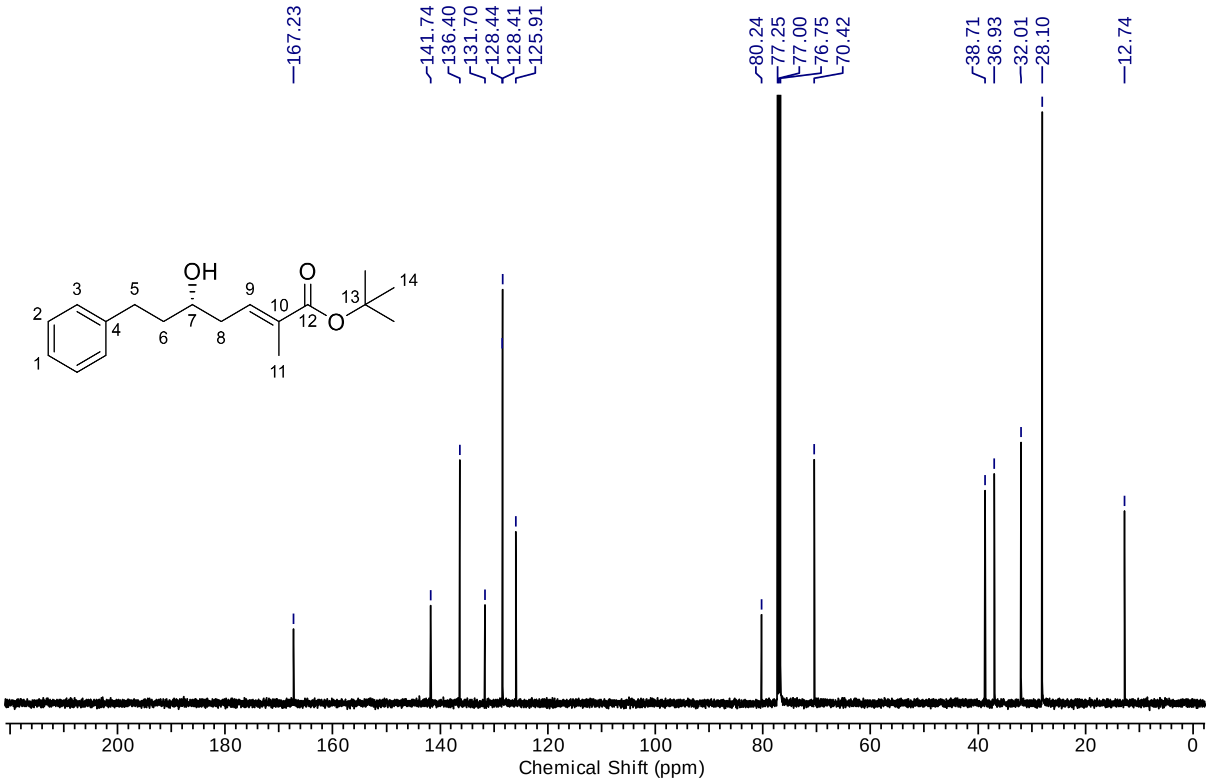

HPLC (6a)

Column: Chiralcel OD-H 5 µm

Eluent: Hexane/*i*PrOH 9/1, 1 ml/min

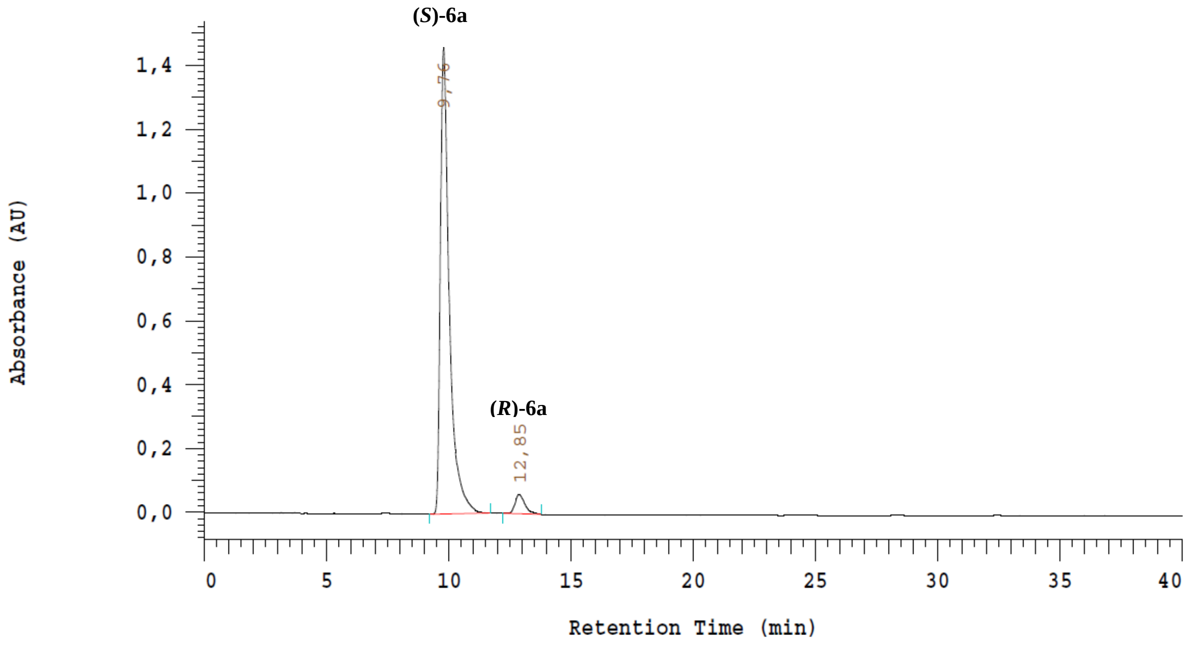

No.	RT	Area	Area %
1	9,76	18825059	95,708
2	12,85	844241	4,292
		19669300	100,000

Ethyl (*S*,*E*)-5-hydroxy-2-methyl-7-phenylhept-2-enoate (6b)

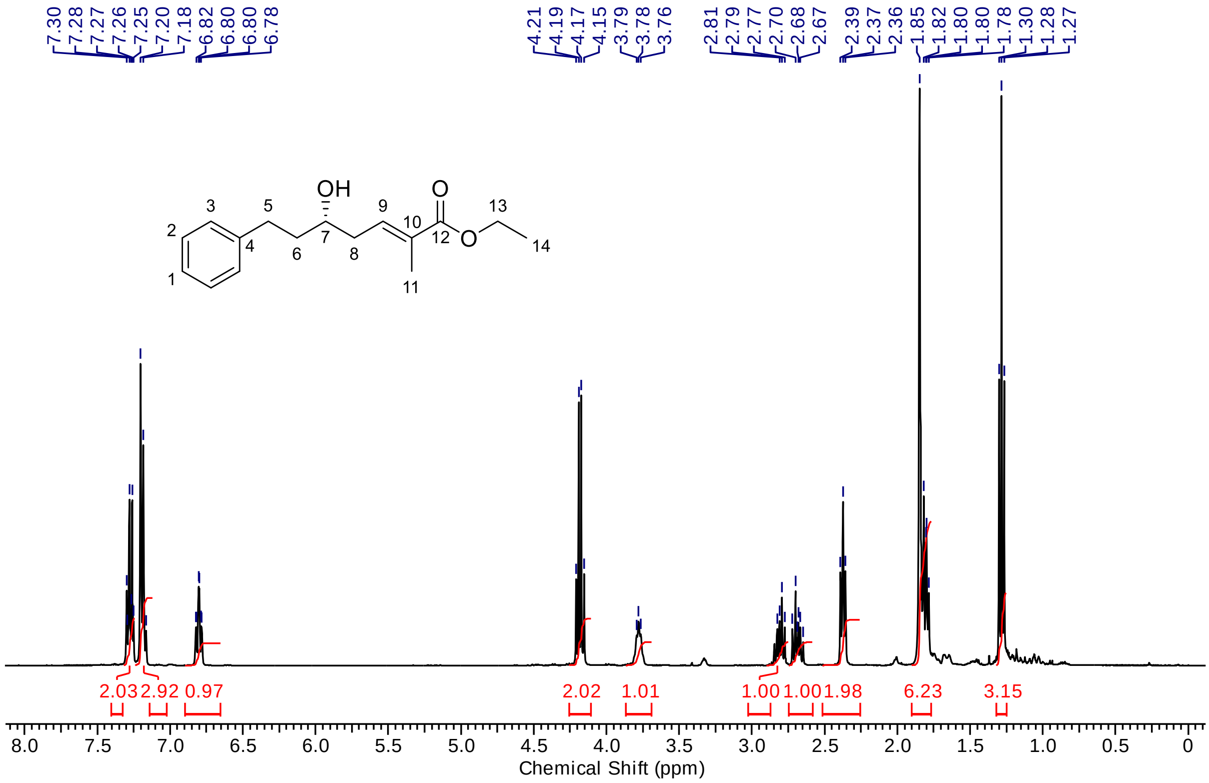

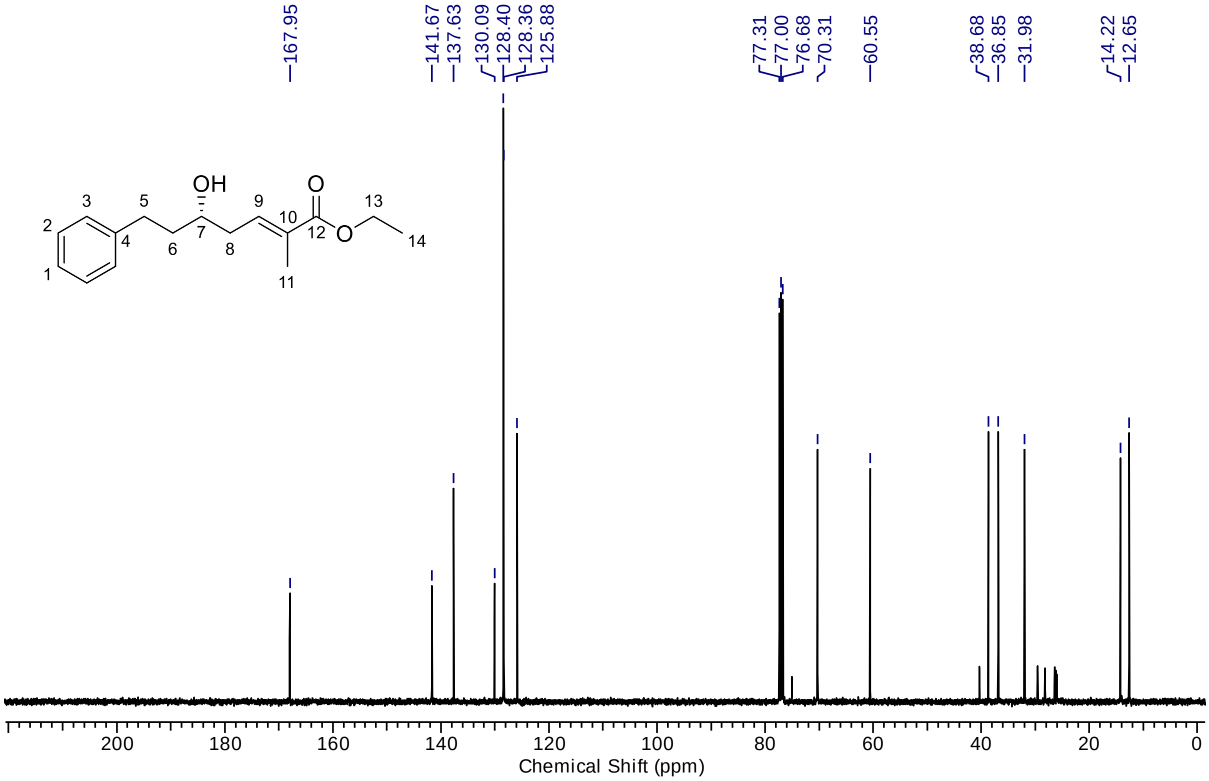

HPLC (6b)

Column: Chiralcel OD-H 5 µm

Eluent: Hexane/*i*PrOH 9/1, 1 ml/min

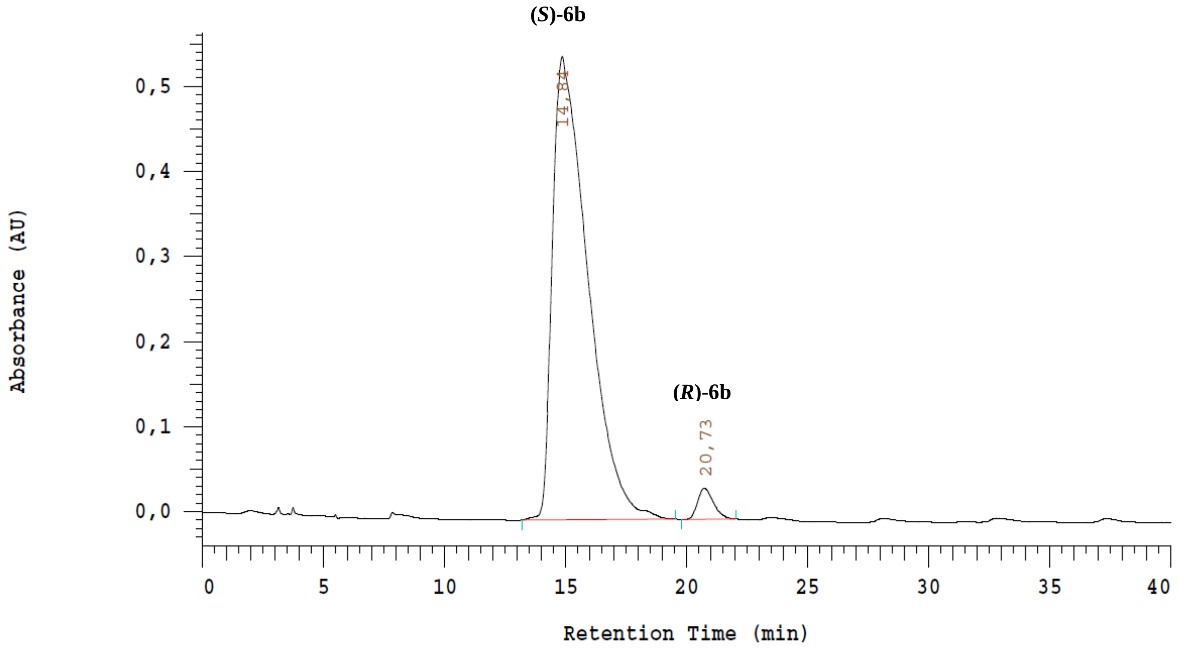

No.	RT	Area	Area %
1	14,84	27742976	97,075
2	20,73	836048	2,925
		28579024	100,000

(*S*,*E*)-5-Hydroxy-2-methyl-7-phenylhept-2-enoic acid (6c)

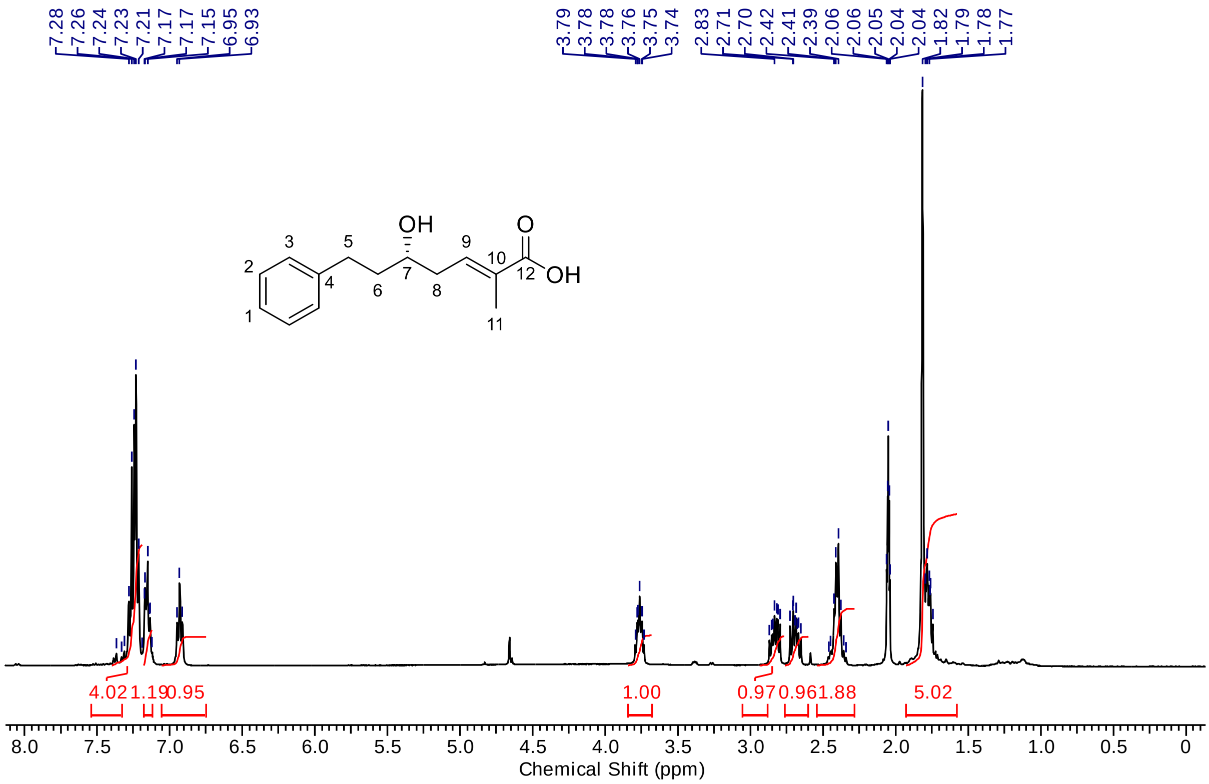

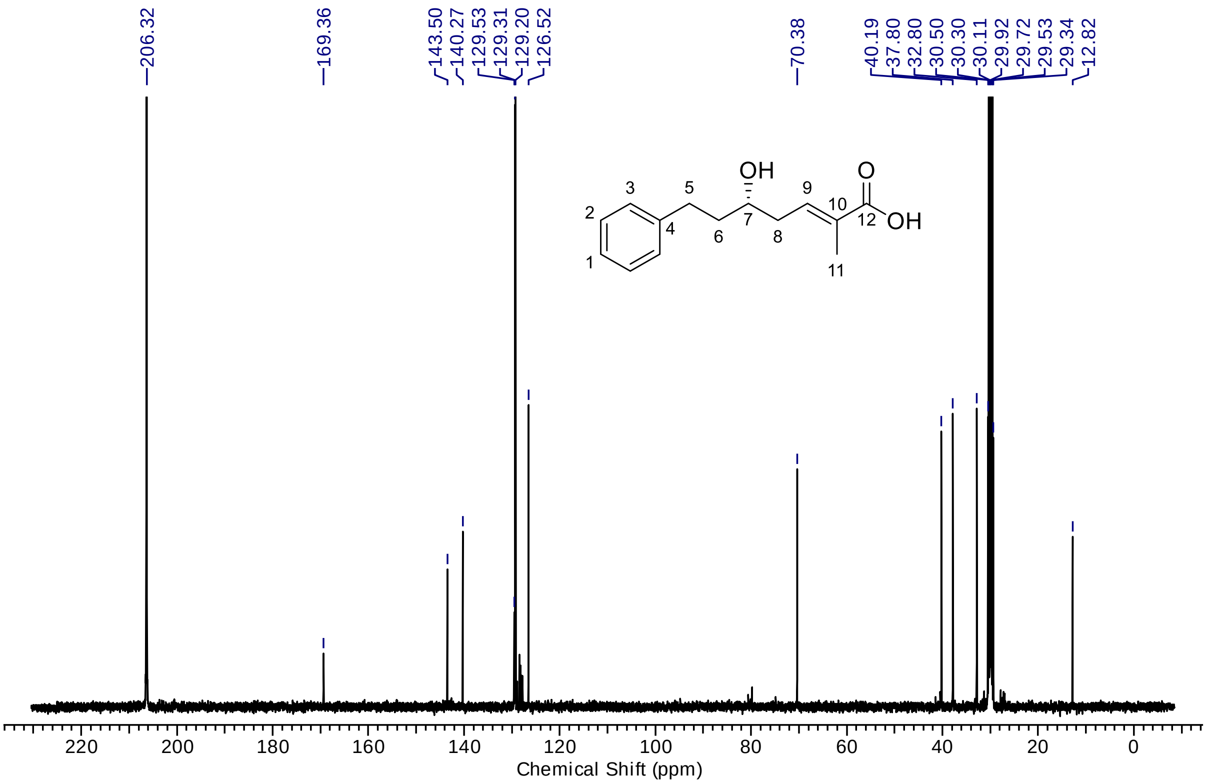

Ethyl (*S*,*E*)-5-hydroxy-2,3-dimethyl-7-phenylhept-2-enoate (6d)

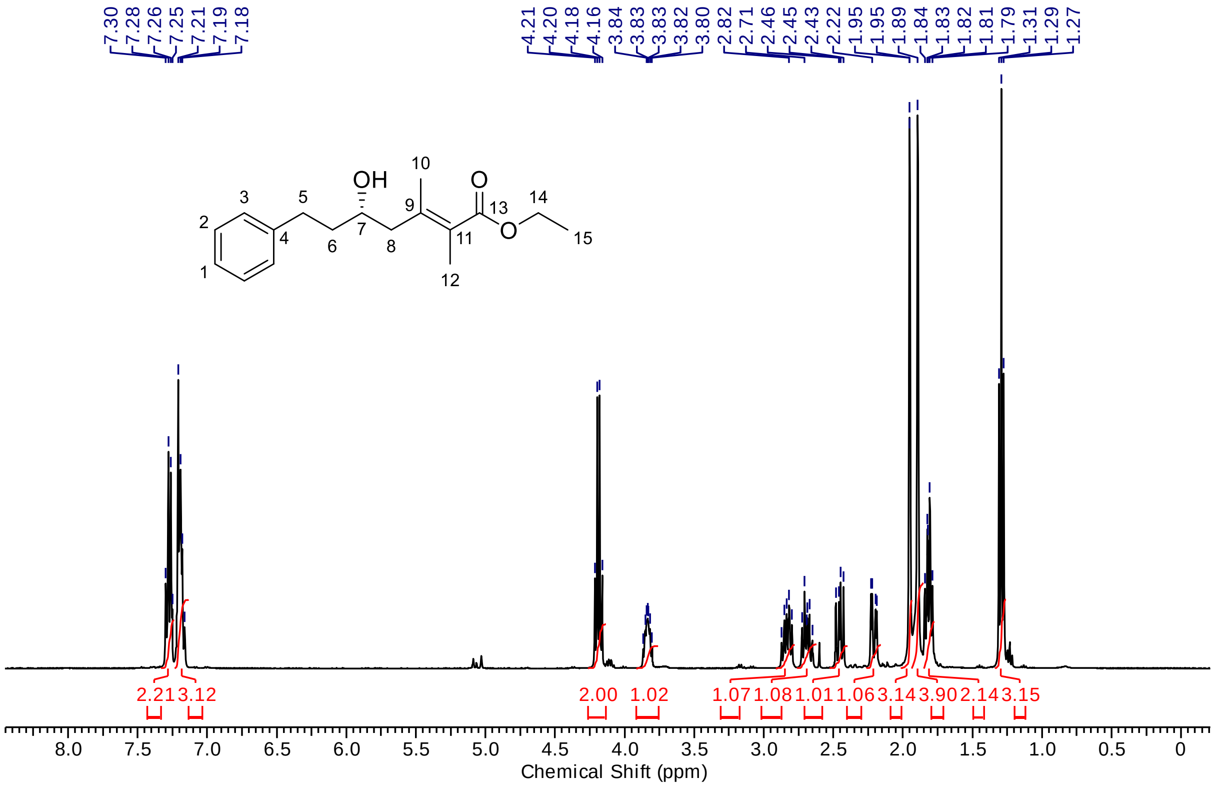

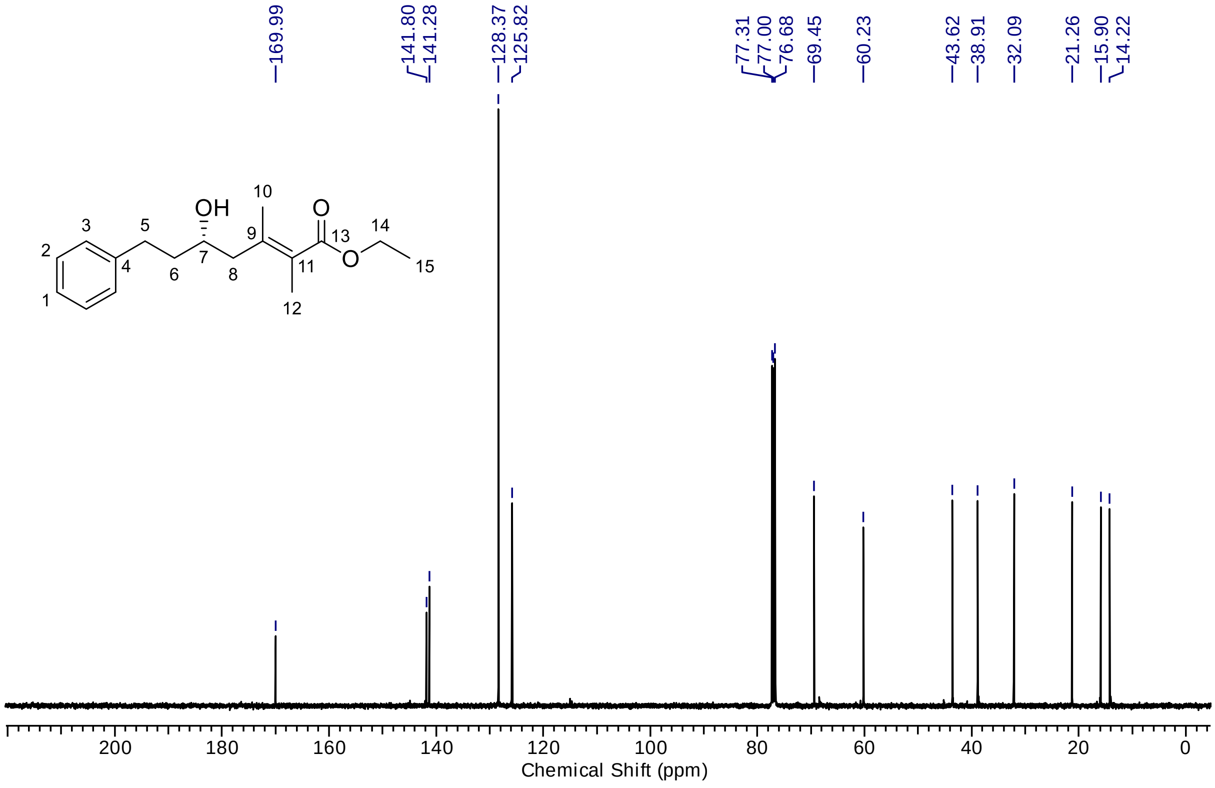

Ethyl (4*S*,5*S*,*E*)-5-hydroxy-2,4-dimethyl-7-phenylhept-2-enoate (6e)

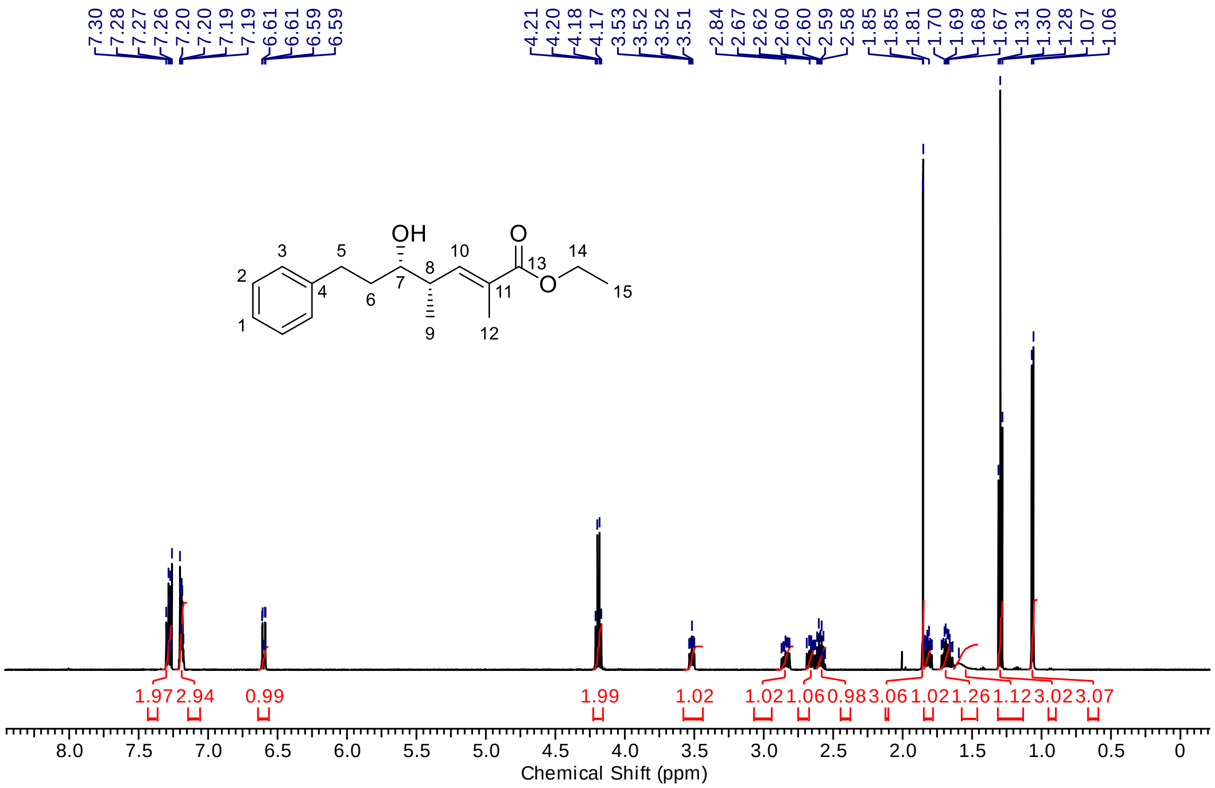

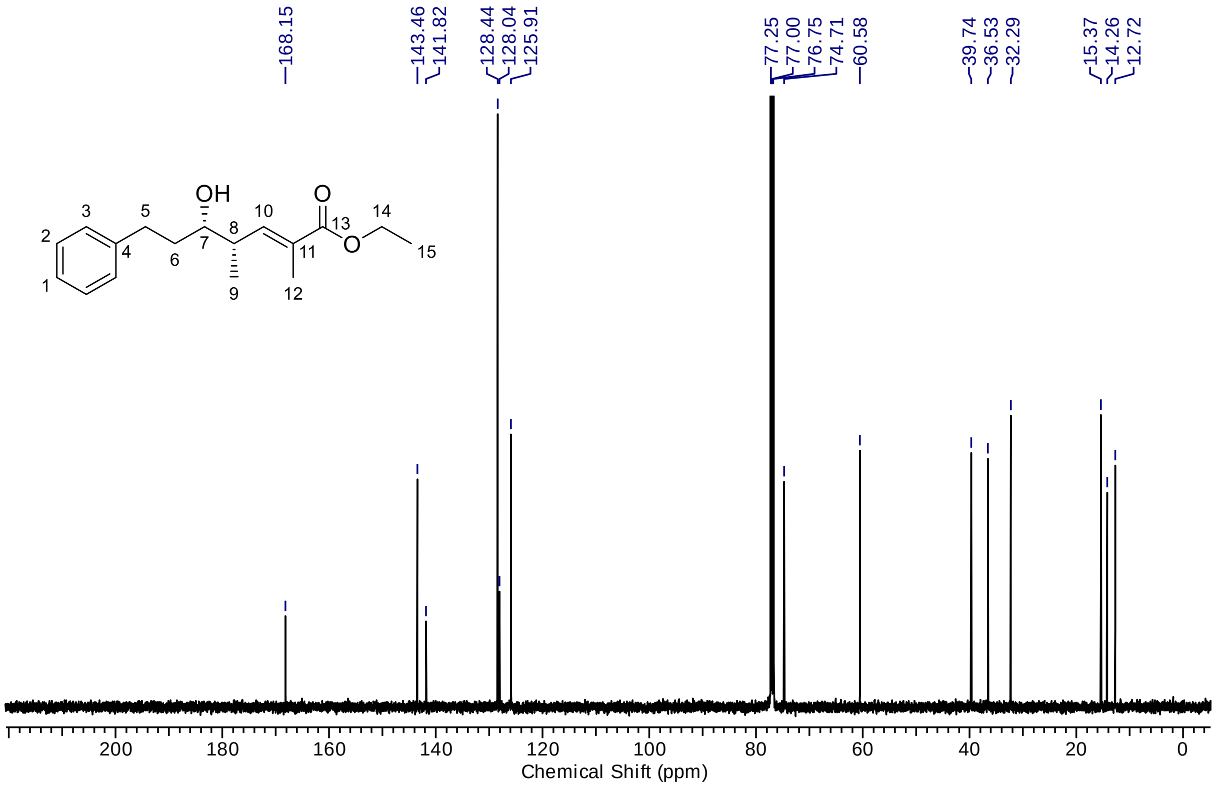

***tert*-Butyl (4*S*,5*S*,*E*)-5-hydroxy-2,4-dimethyl-7-phenylhept-2-enoate (6f)**

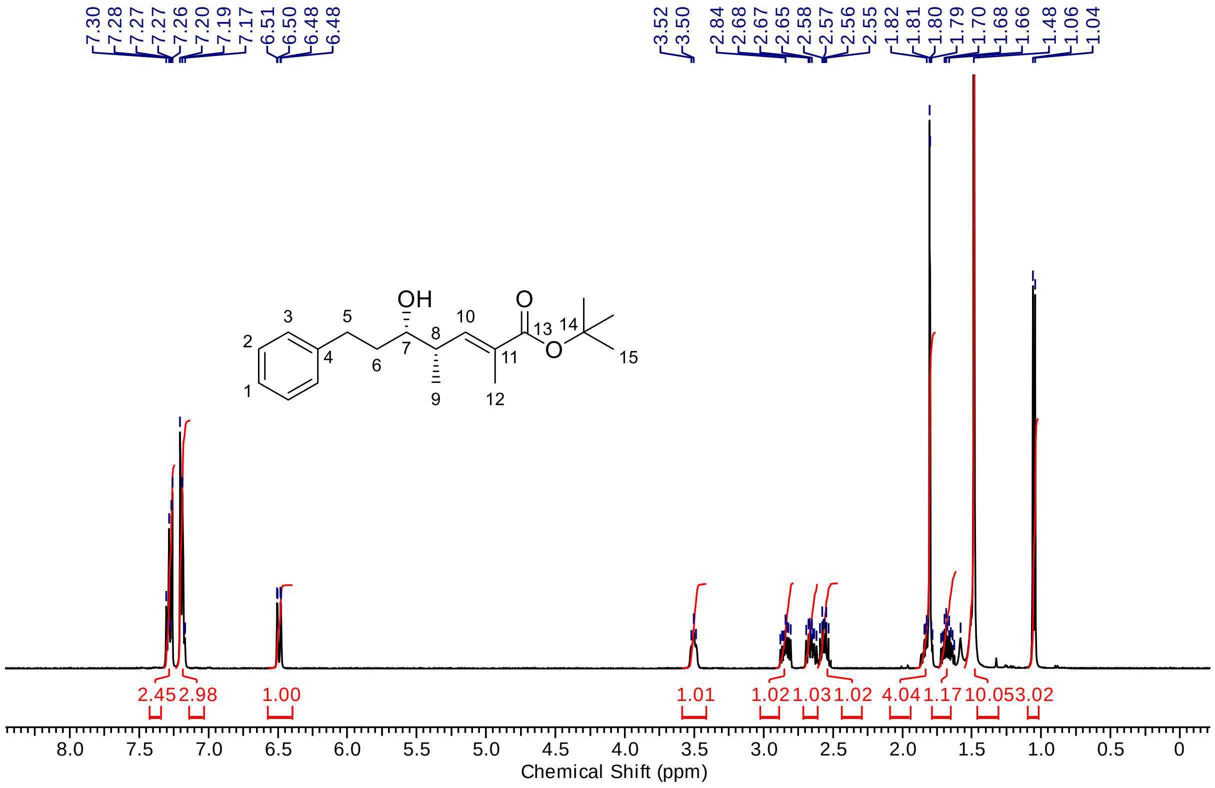

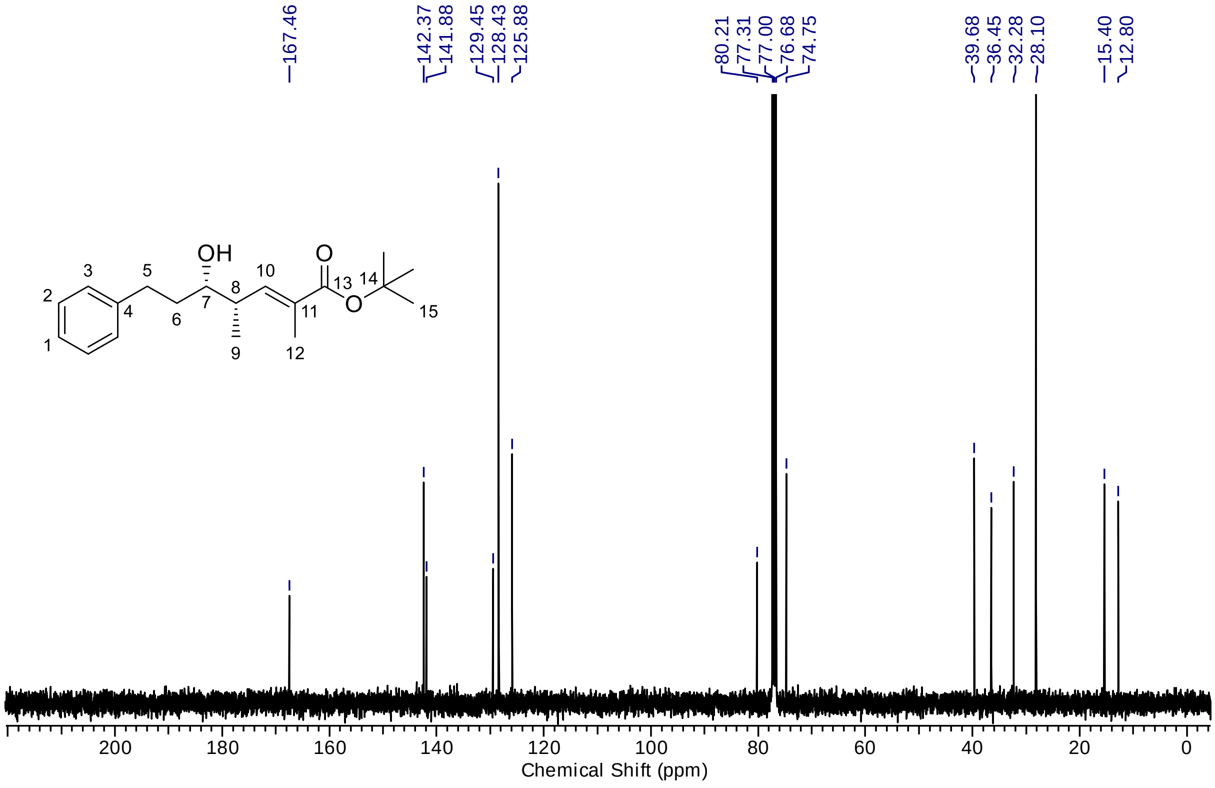

(4*S*,5*S*)-4,5-Diphenyl-2-propyl-1,3,2-dioxaborolane (A')

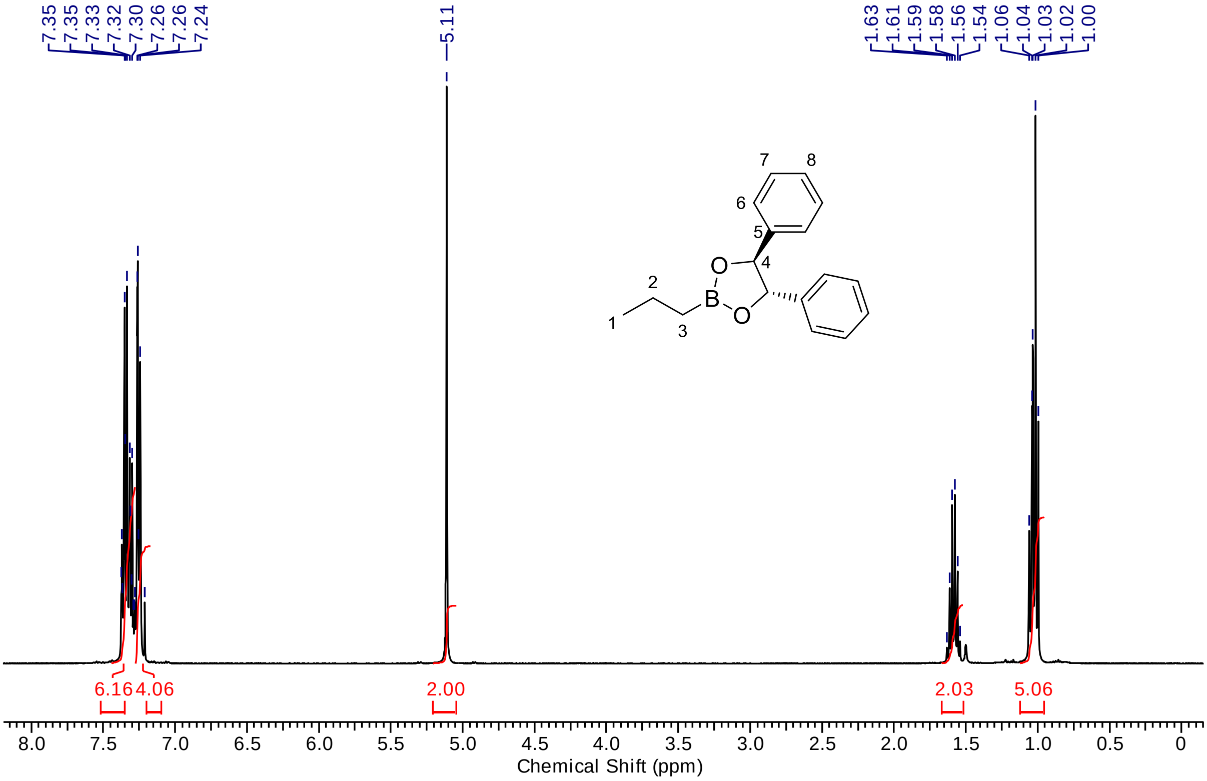

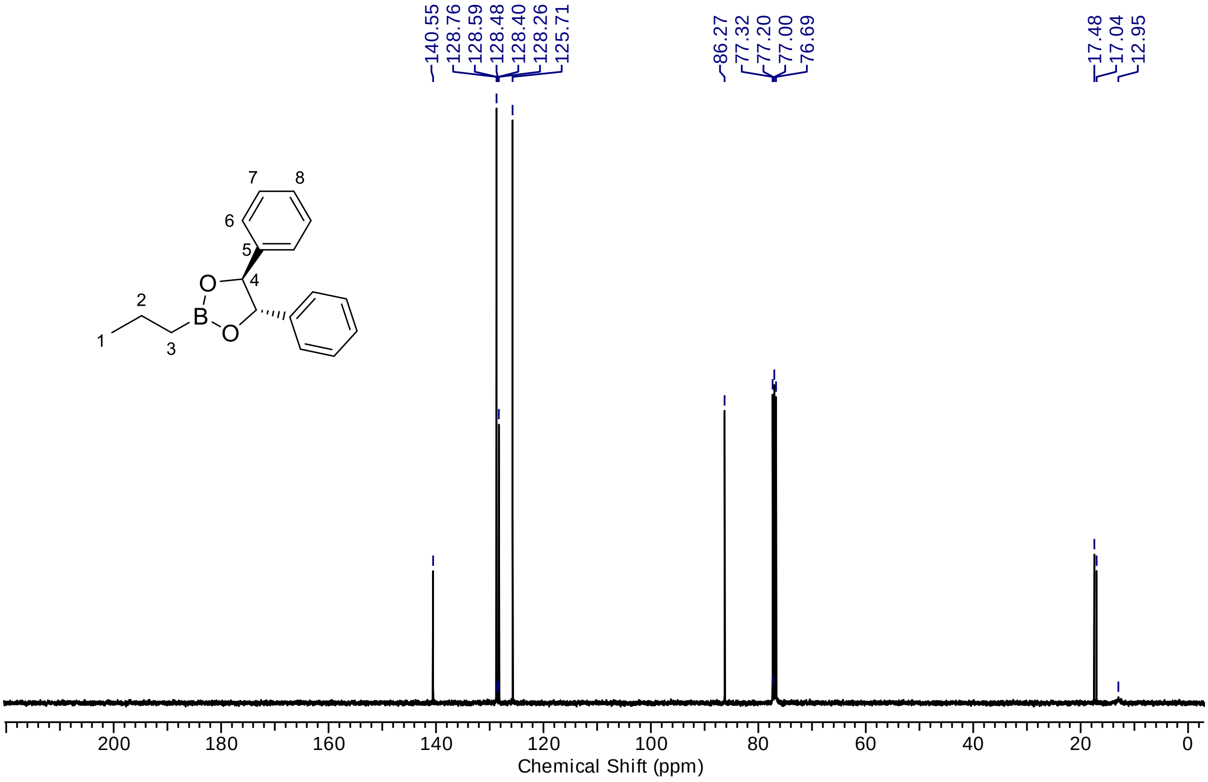

(4*S*,5*S*)-4,5-dicyclohexyl-2-propyl-1,3,2-dioxaborolane (A)

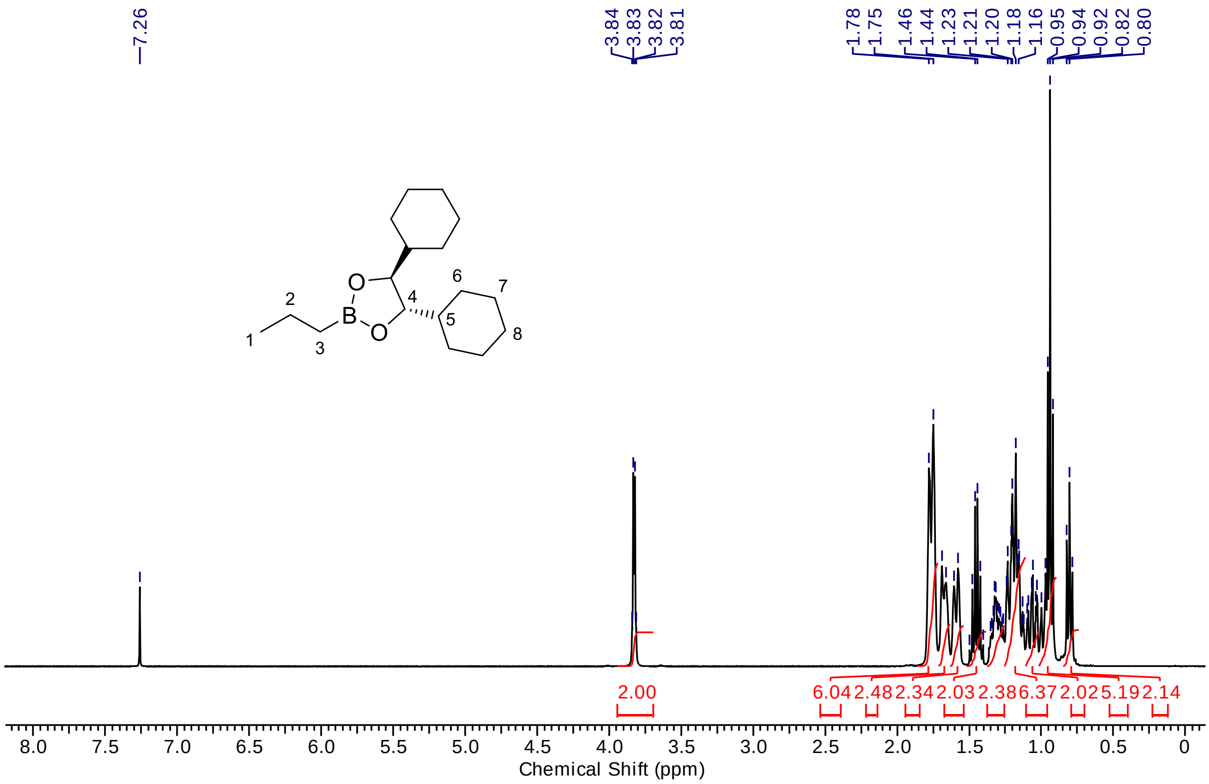

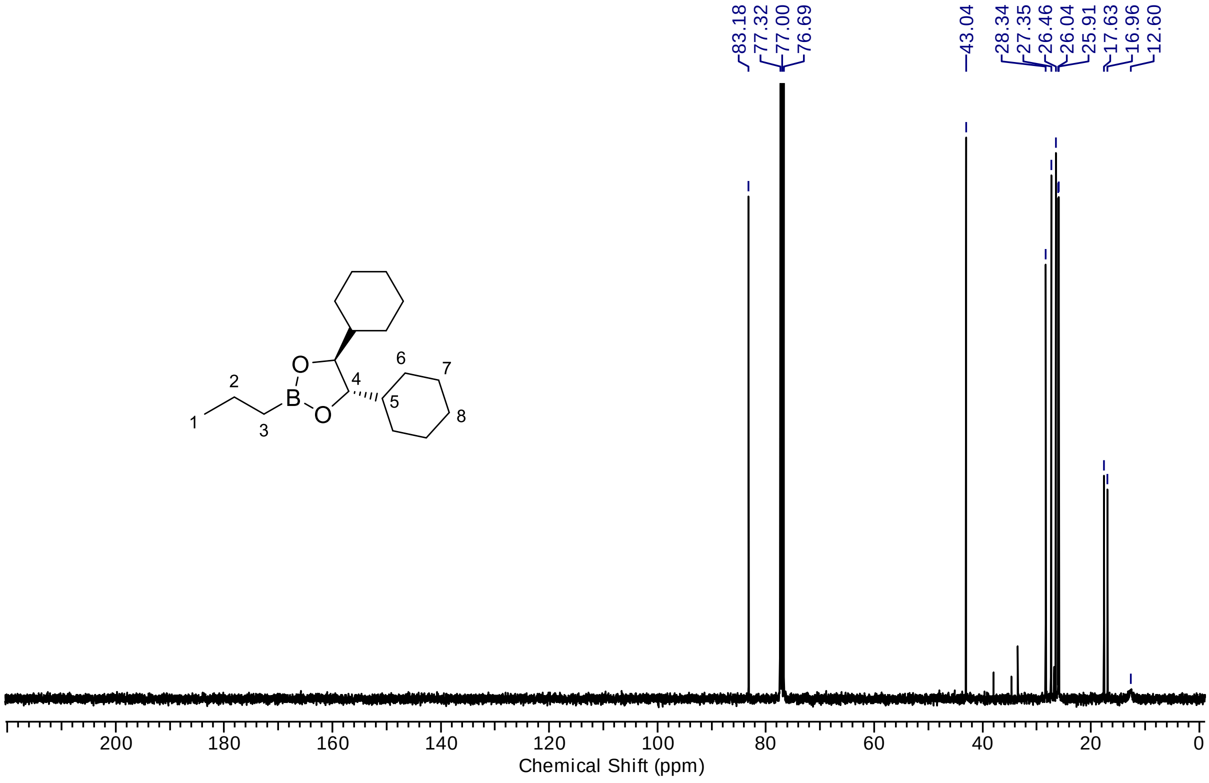

(4*S*,5*S*)-2-Isobutyl-4,5-diisopropyl-1,3,2-dioxaborolane (B)

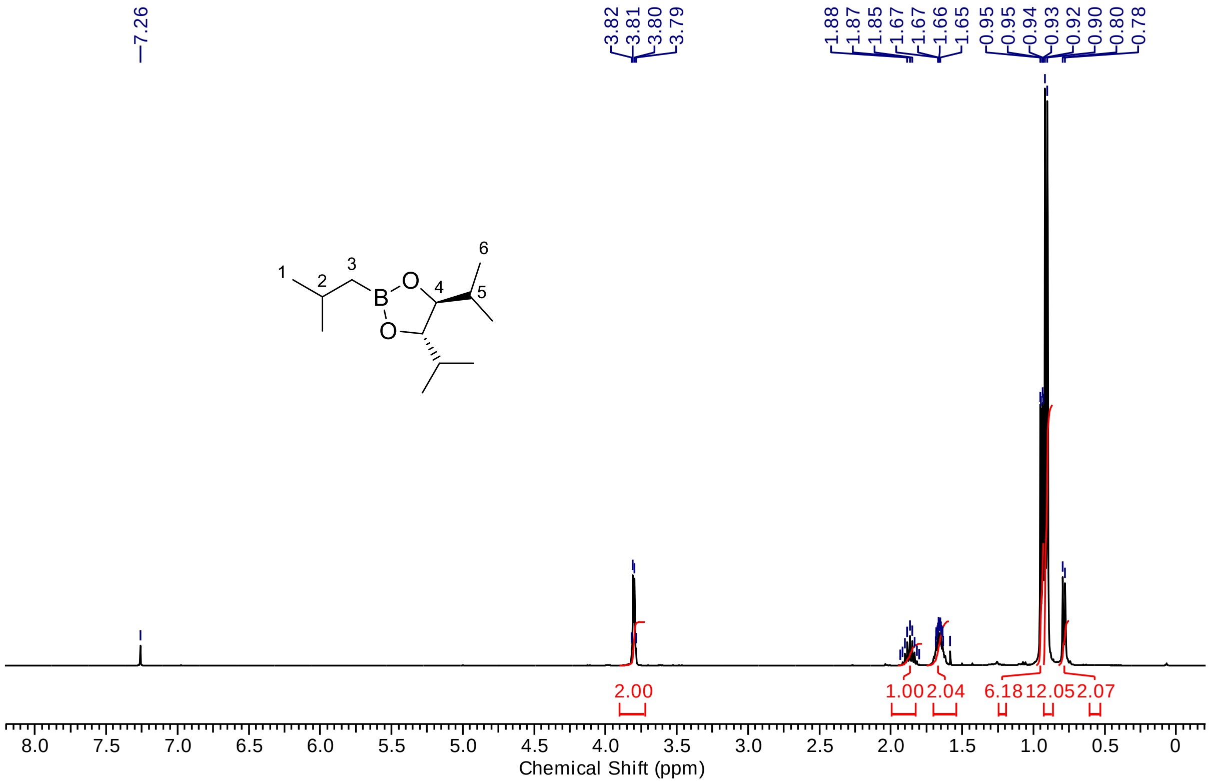

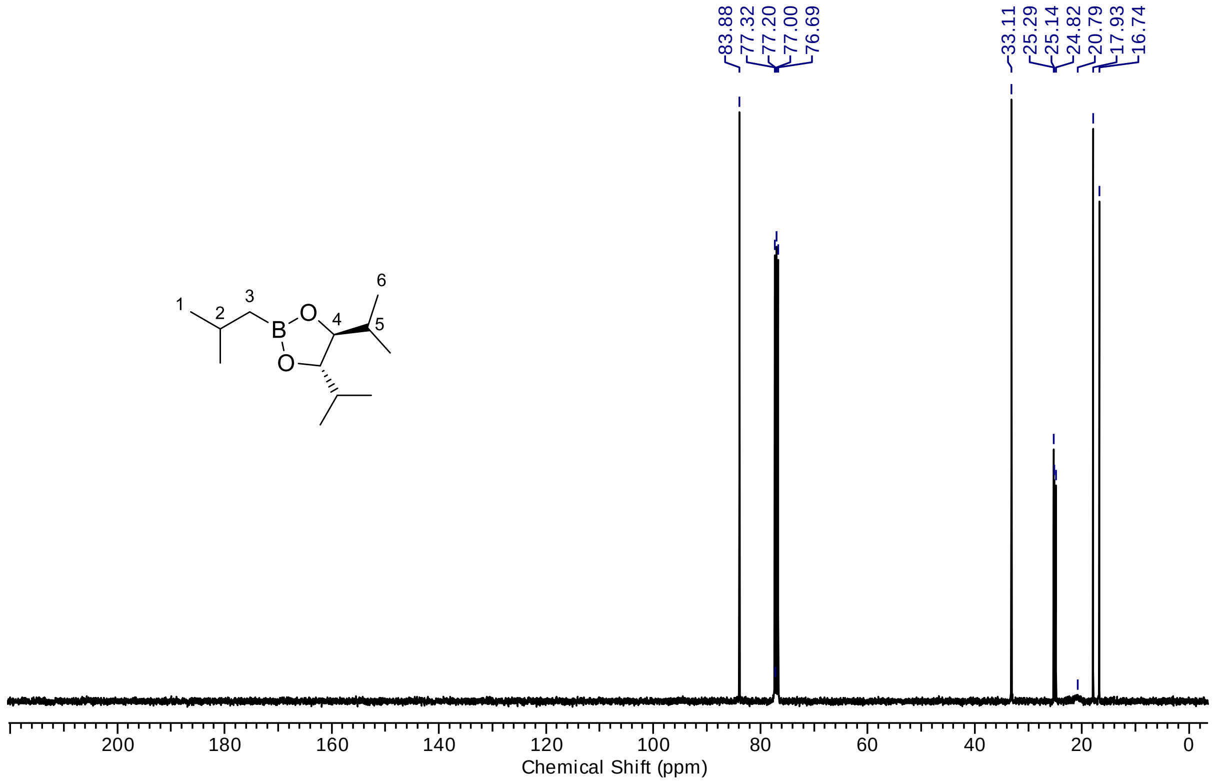

tert-Butyl{4-[(4*S*,5*S*)-4,5-dicyclohexyl-1,3,2-dioxaborolan-2-yl]butoxy}dimethylsilane (C)

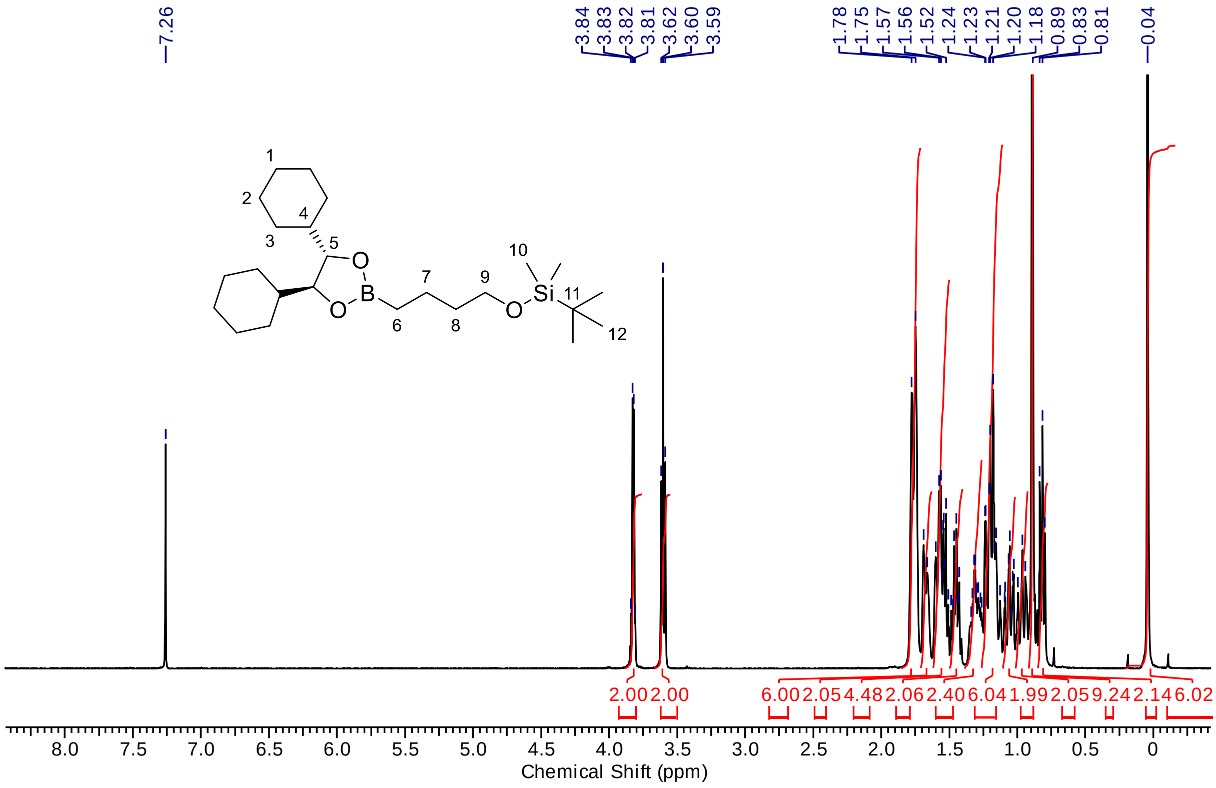

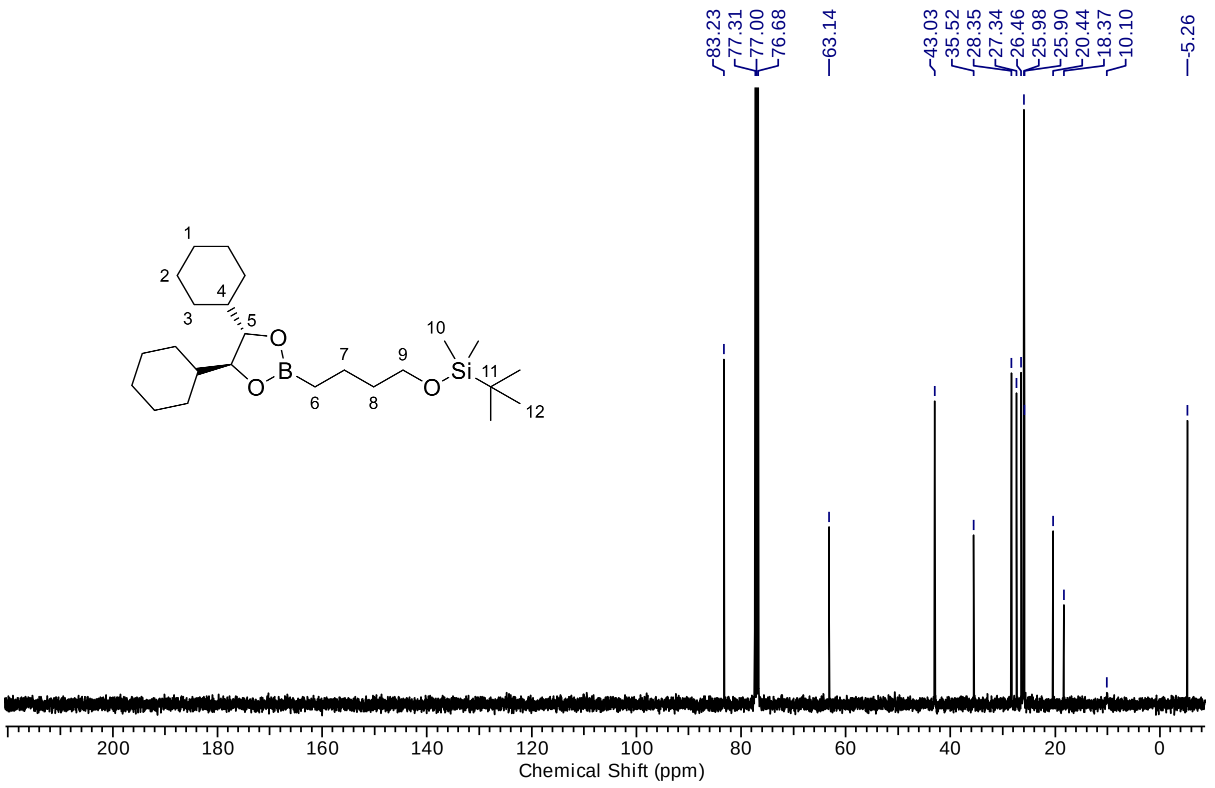

(4*S*,5*S*)-2-[(*S*)-1-(Benzyloxy)butyl]-4,5-dicyclohexyl-1,3,2-dioxaborolane (D')

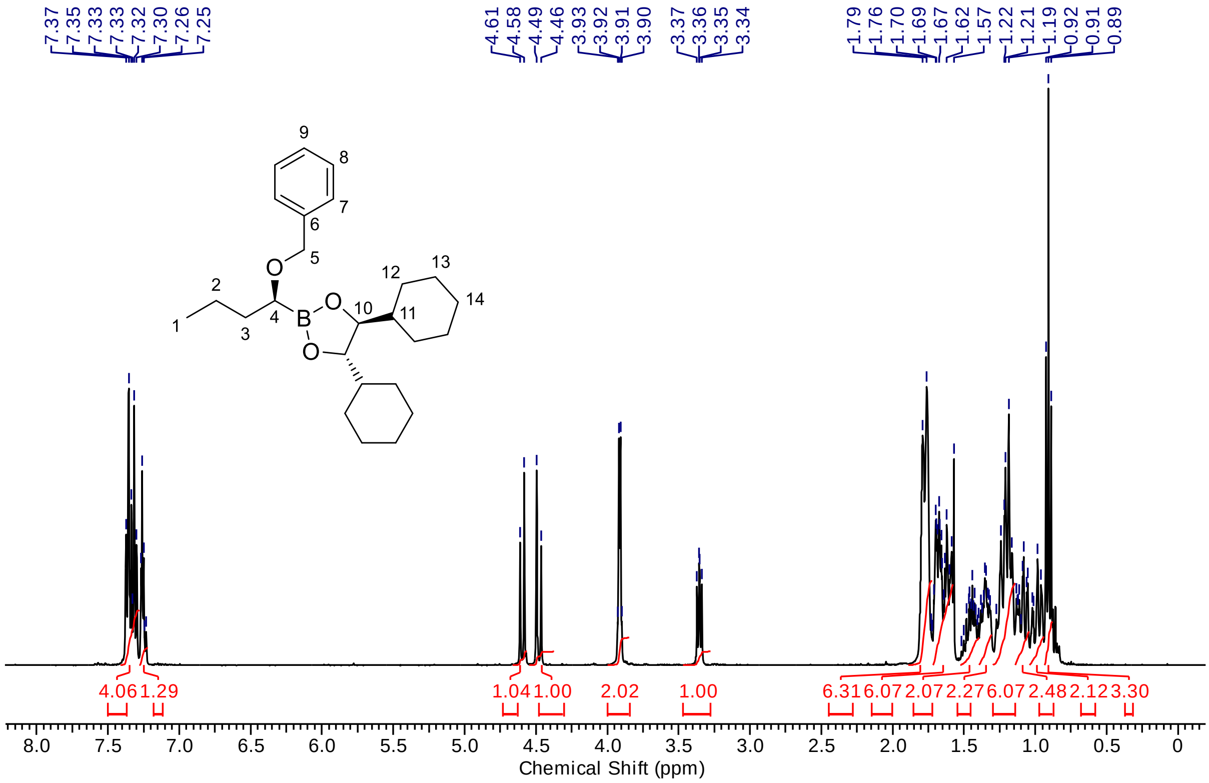

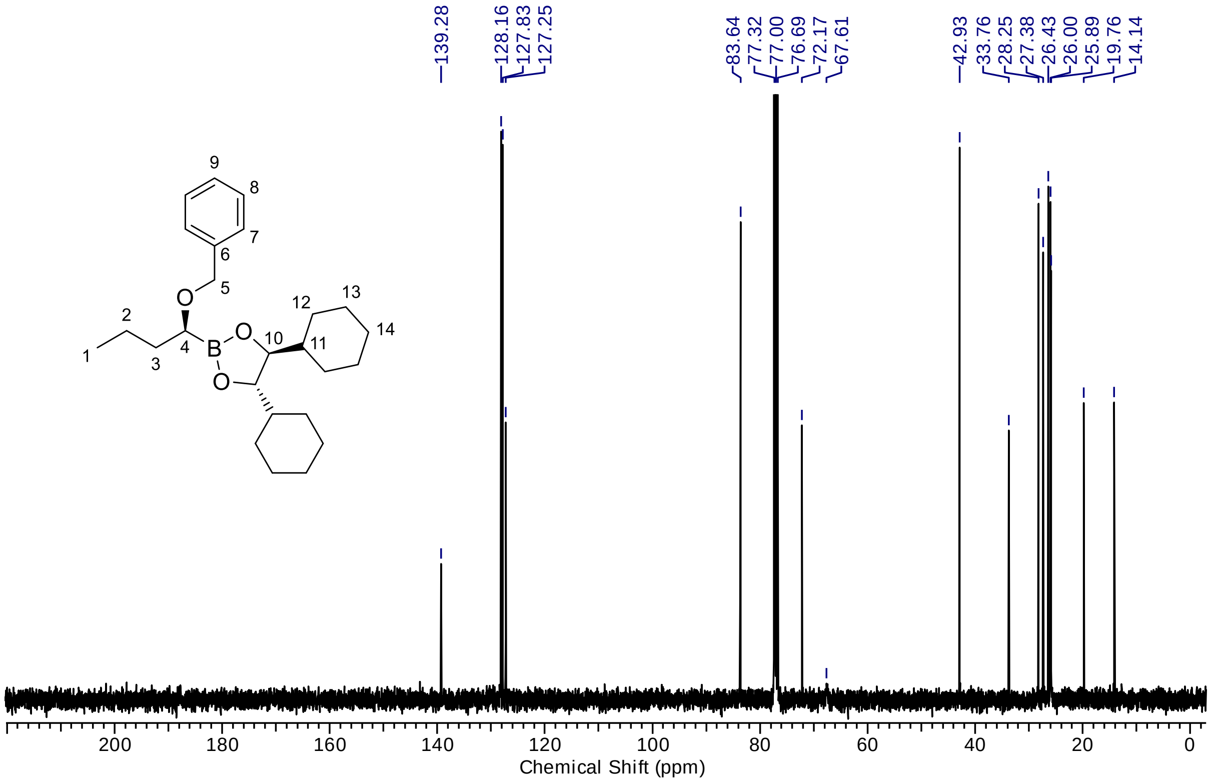

(4*S*,5*S*)-2-[(2*R*,3*R*)-3-(Benzyloxy)hexan-2-yl]-4,5-dicyclohexyl-1,3,2-dioxaborolane (D)

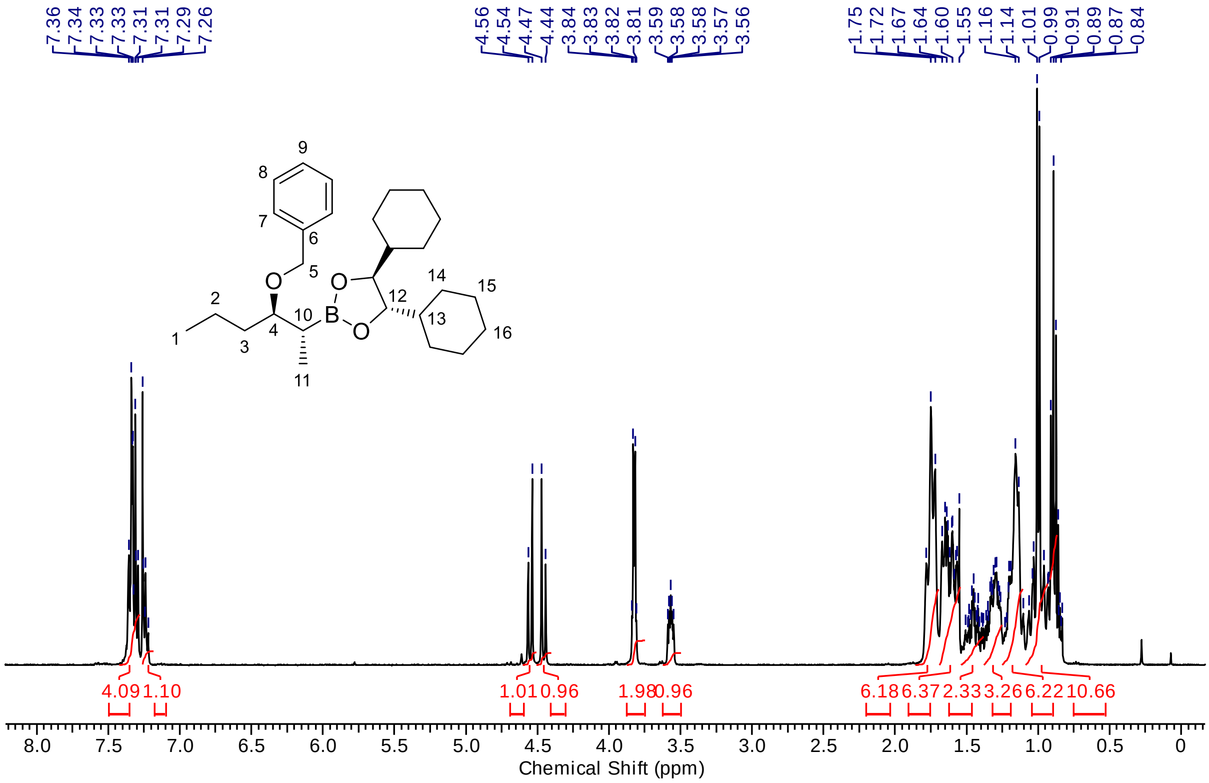

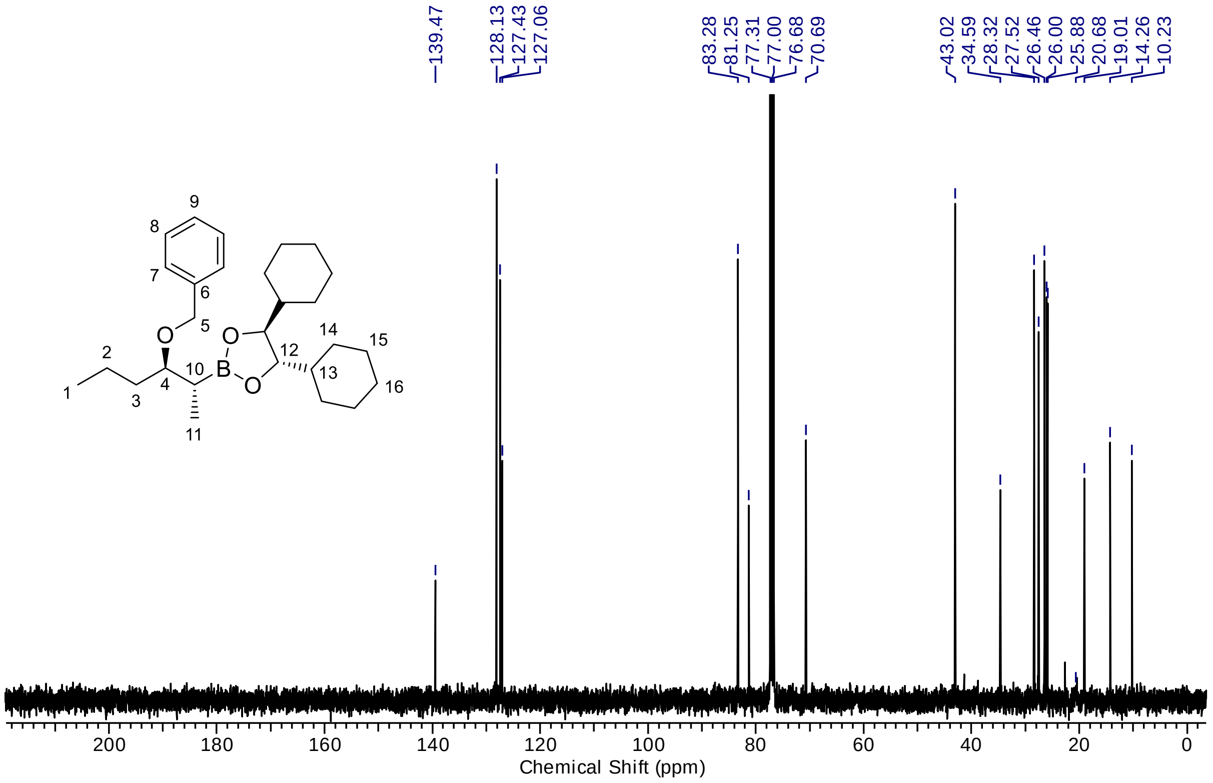

(4S,5S)-2,4,5-Tricyclohexyl-1, 3,2-dioxaborolane (E)

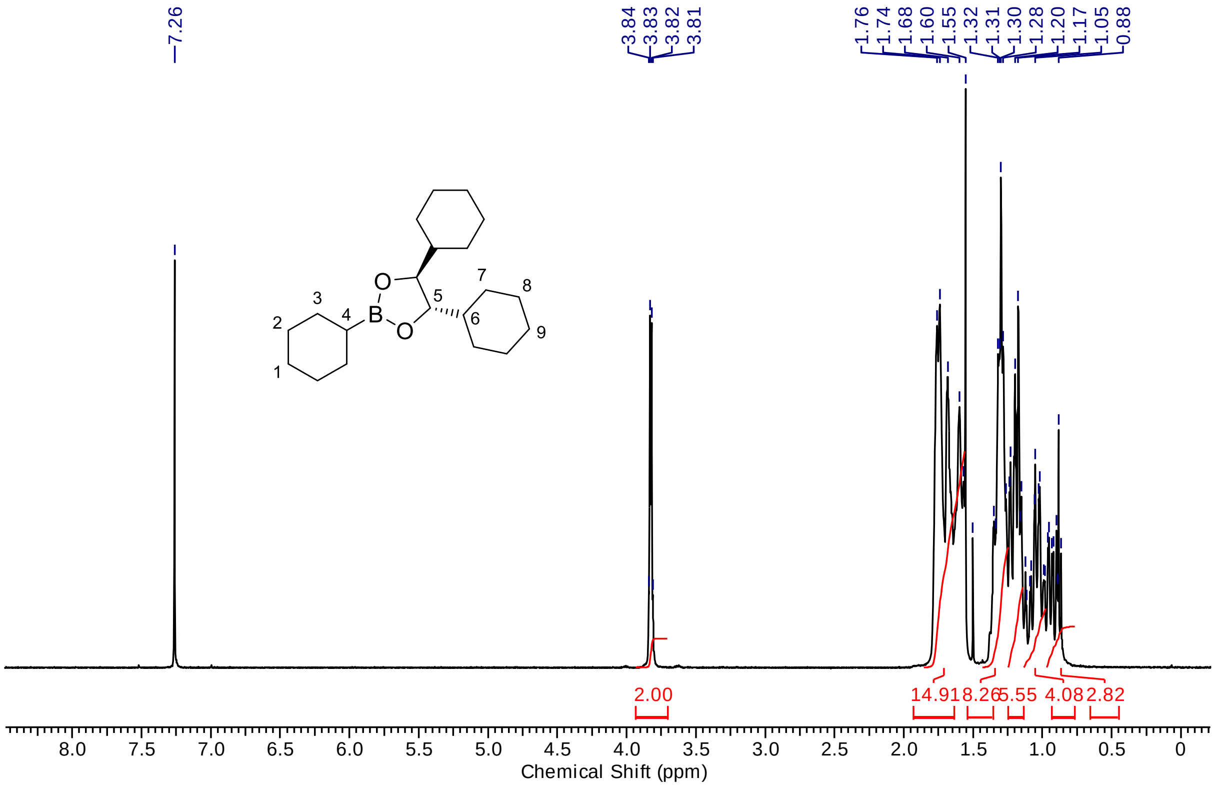

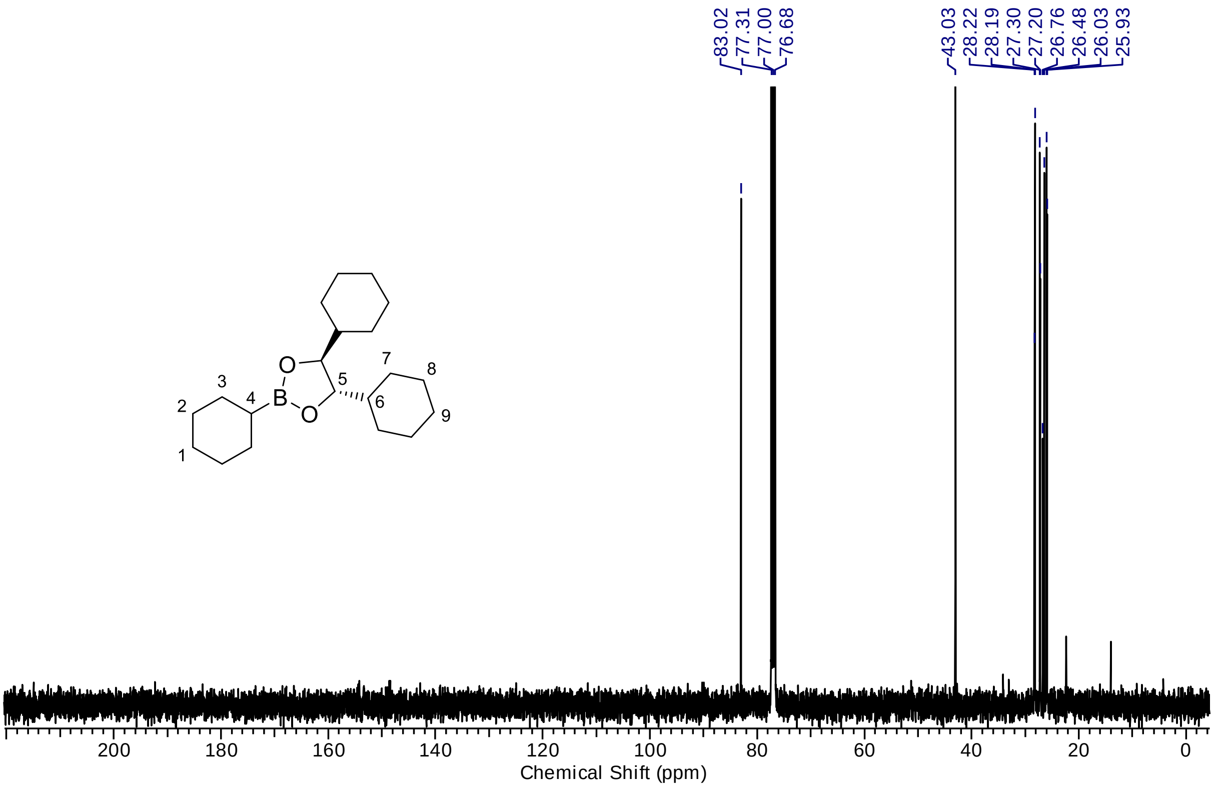

(4*S*,5*S*)-4,5-Dicyclohexyl-2-[(*R*)-hexan-3-yl]-1,3,2-dioxaborolane (F)

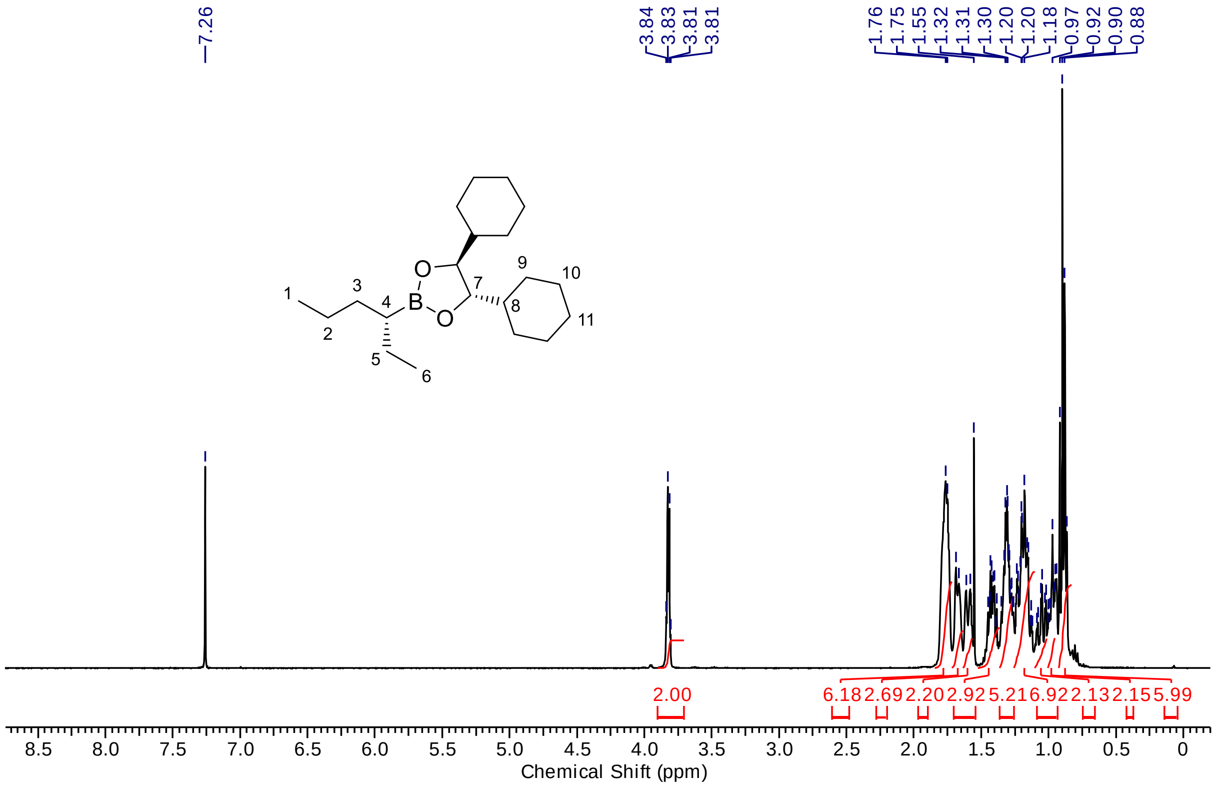

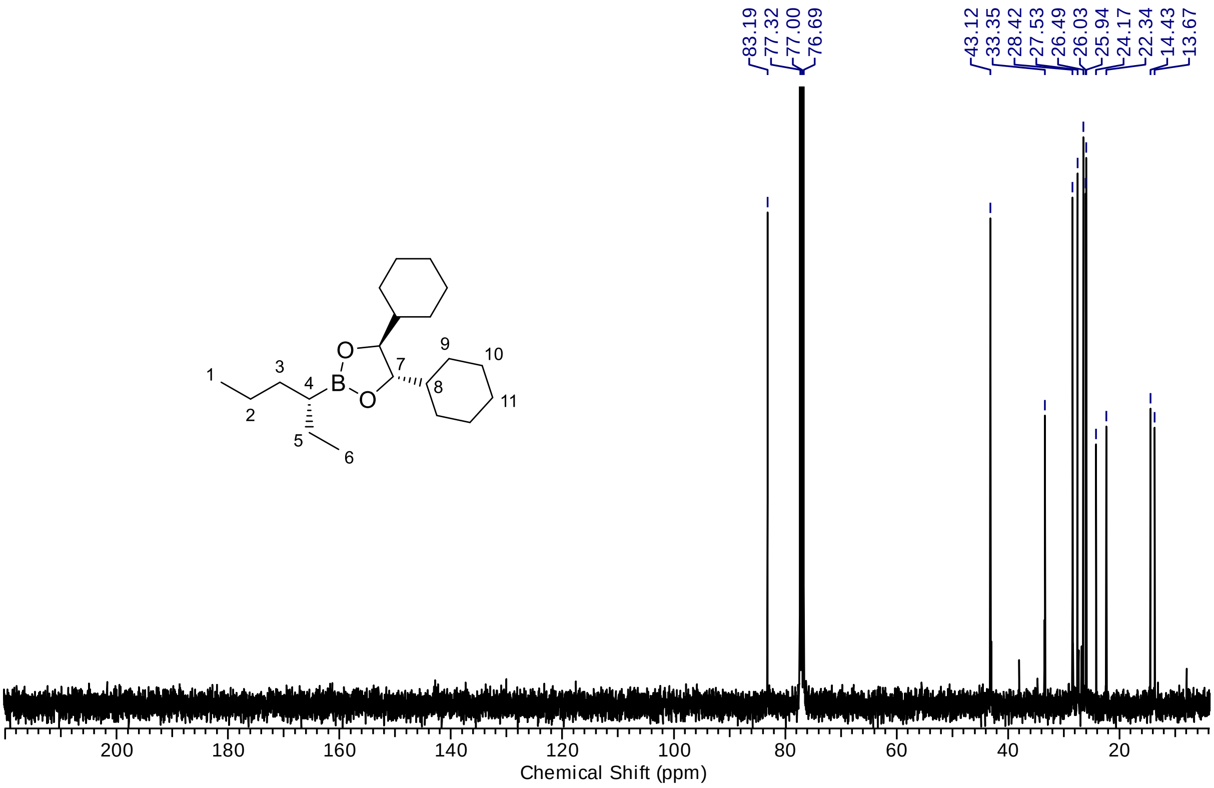

Ethyl (*S*,*E*)-5-[(4*S*,5*S*)-4,5-dicyclohexyl-1, 3,2-dioxaborolan-2-yl]-2-methyloct-2-enoate (7a)

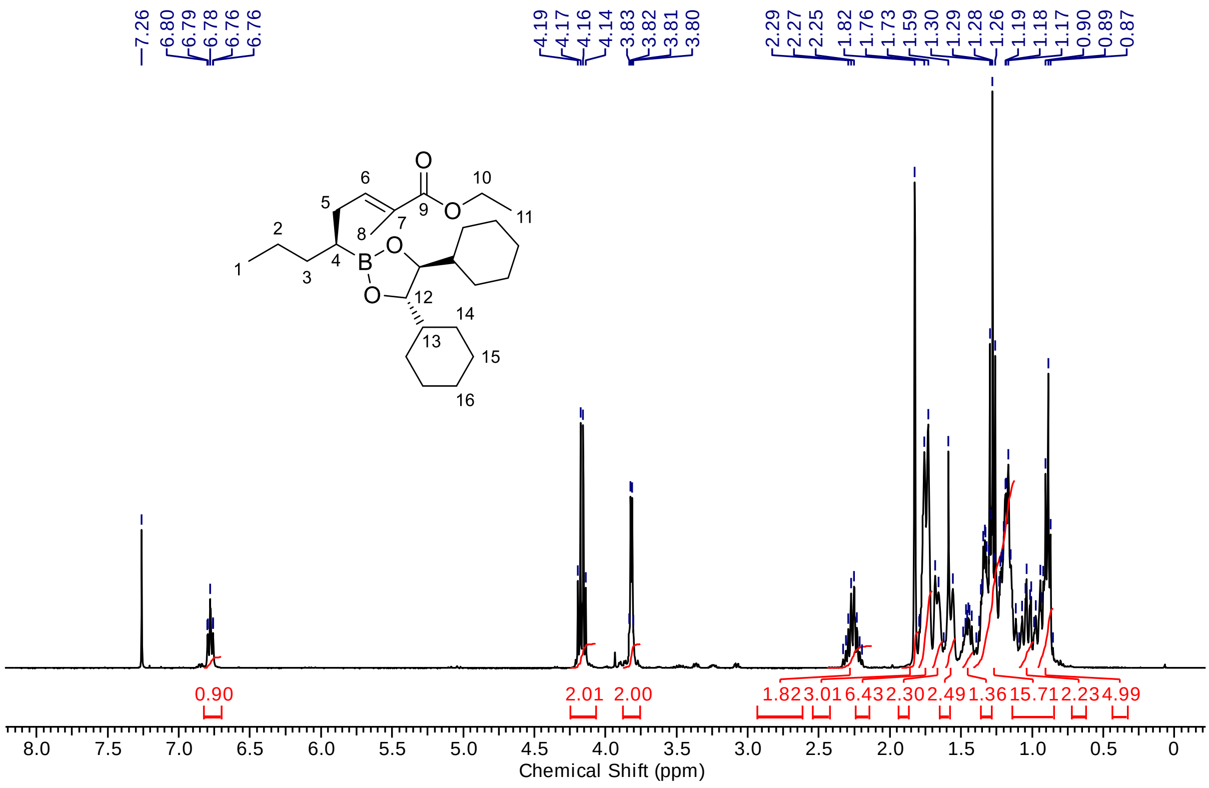

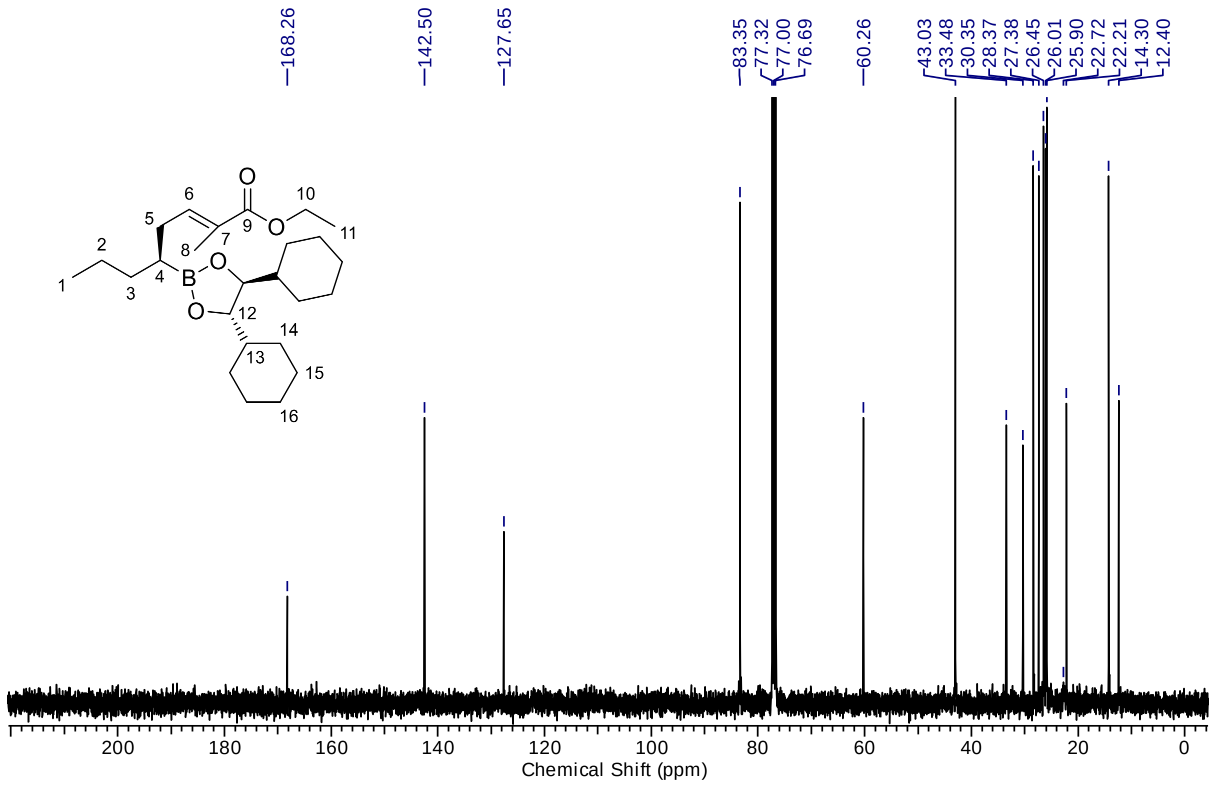

Ethyl (*S*,*E*)- 5-[(4*S*,5*S*)-4,5-Diisopropyl-1,3,2-dioxaborolan-2-yl]-2,7-dimethyloct-2-enoate (7b)

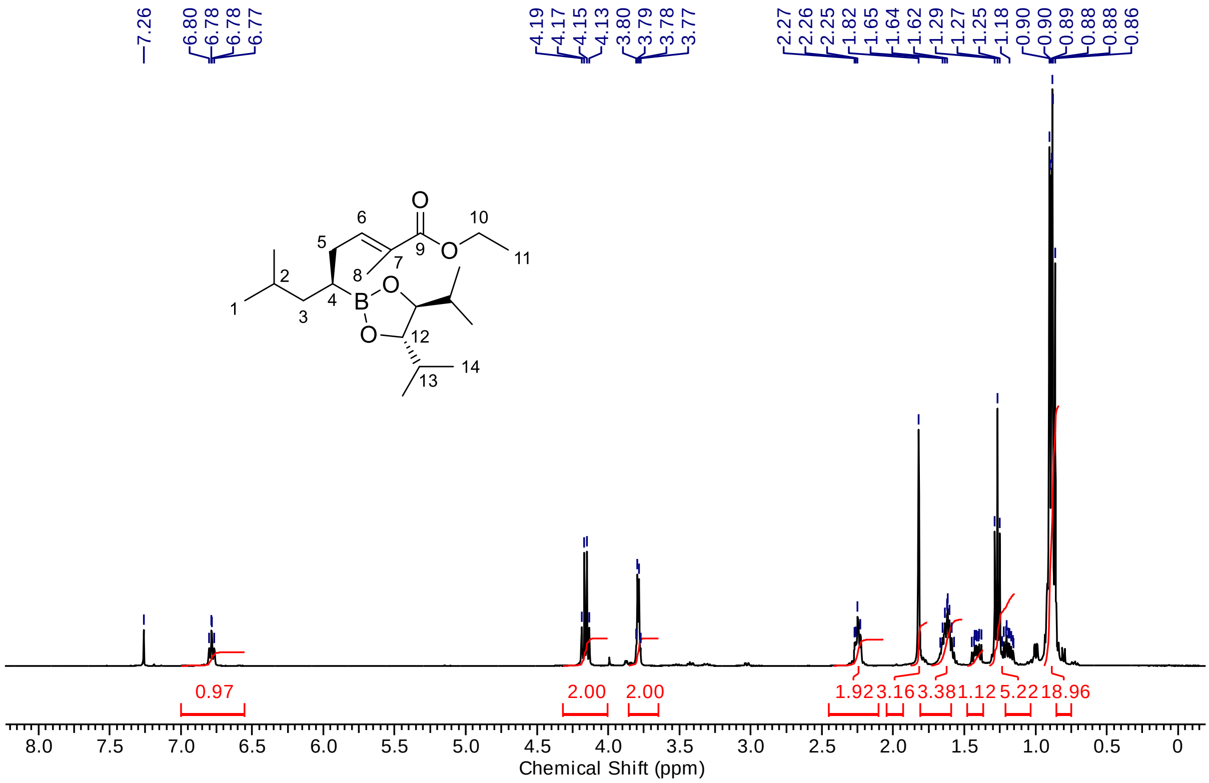

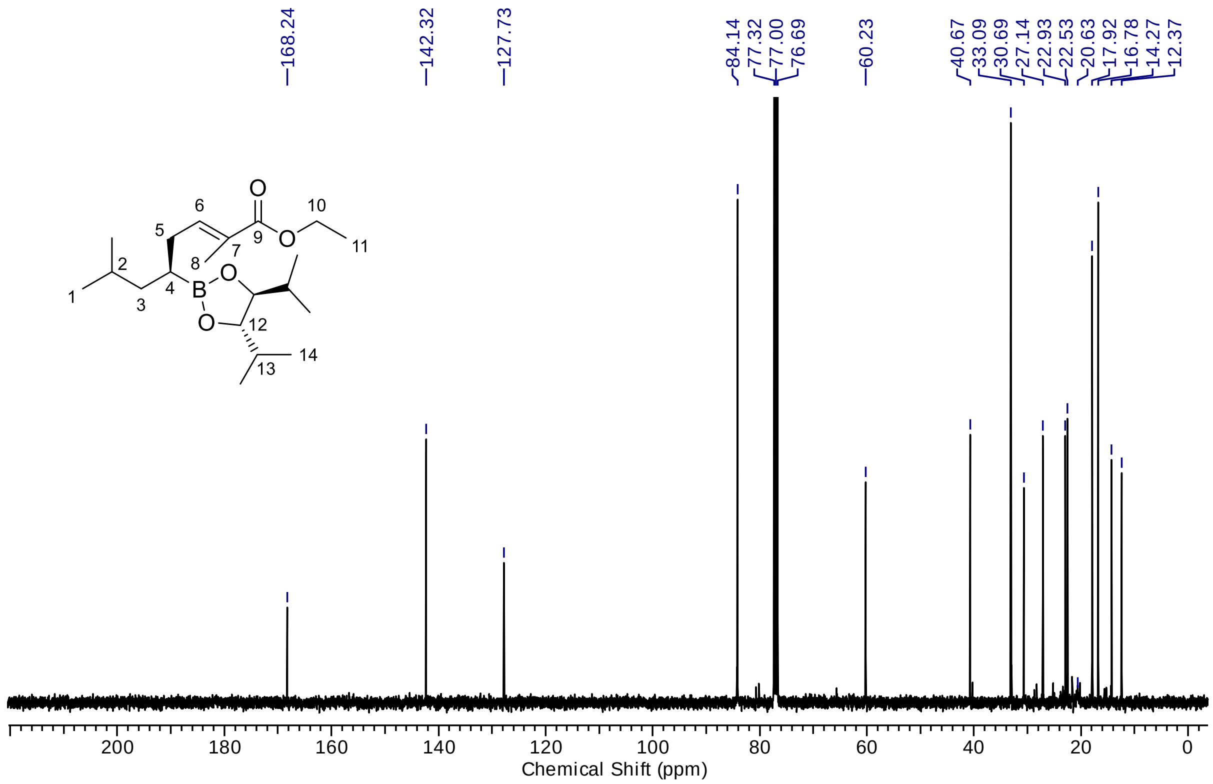

Ethyl (*S*,*E*)-9-[(*tert*-butyldimethylsilyl)oxy]-5-[(4*S*,5*S*)-4,5-dicyclohexyl-1,3,2-dioxaborolan-2-yl]-2-methylnon-2-enoate (7c)

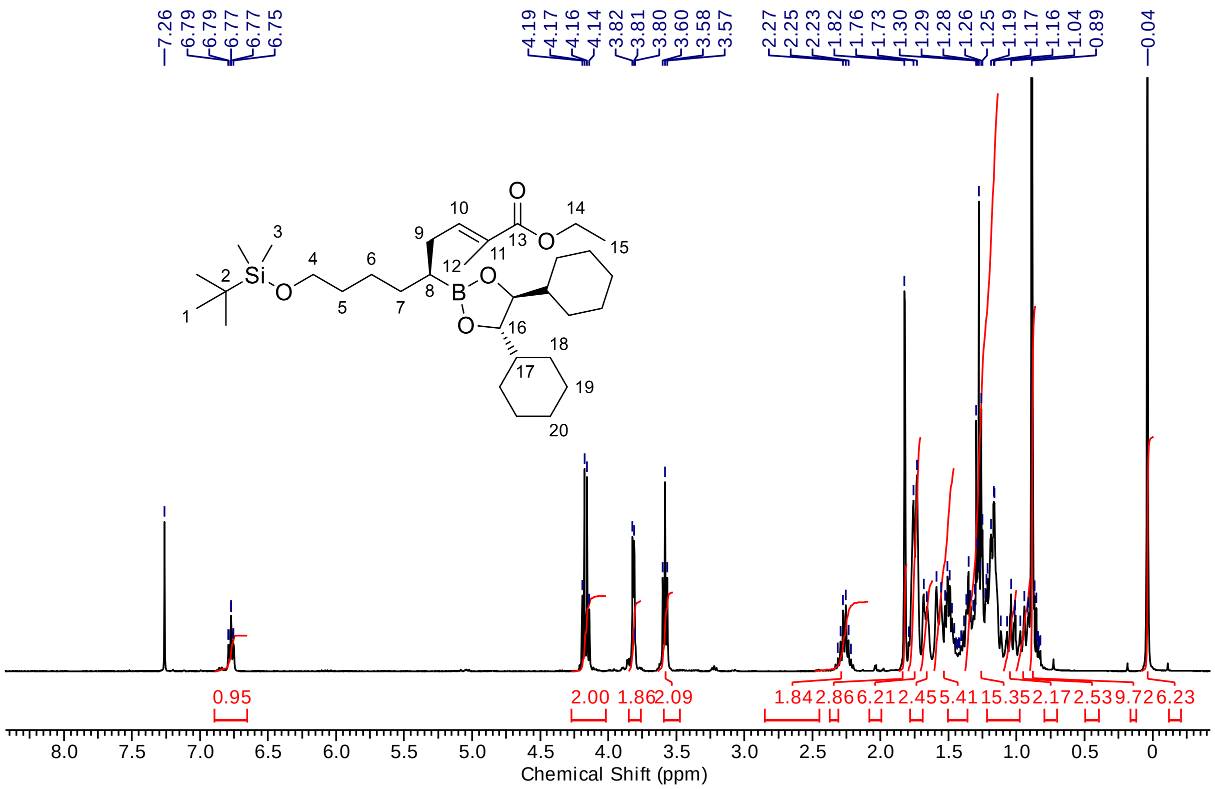

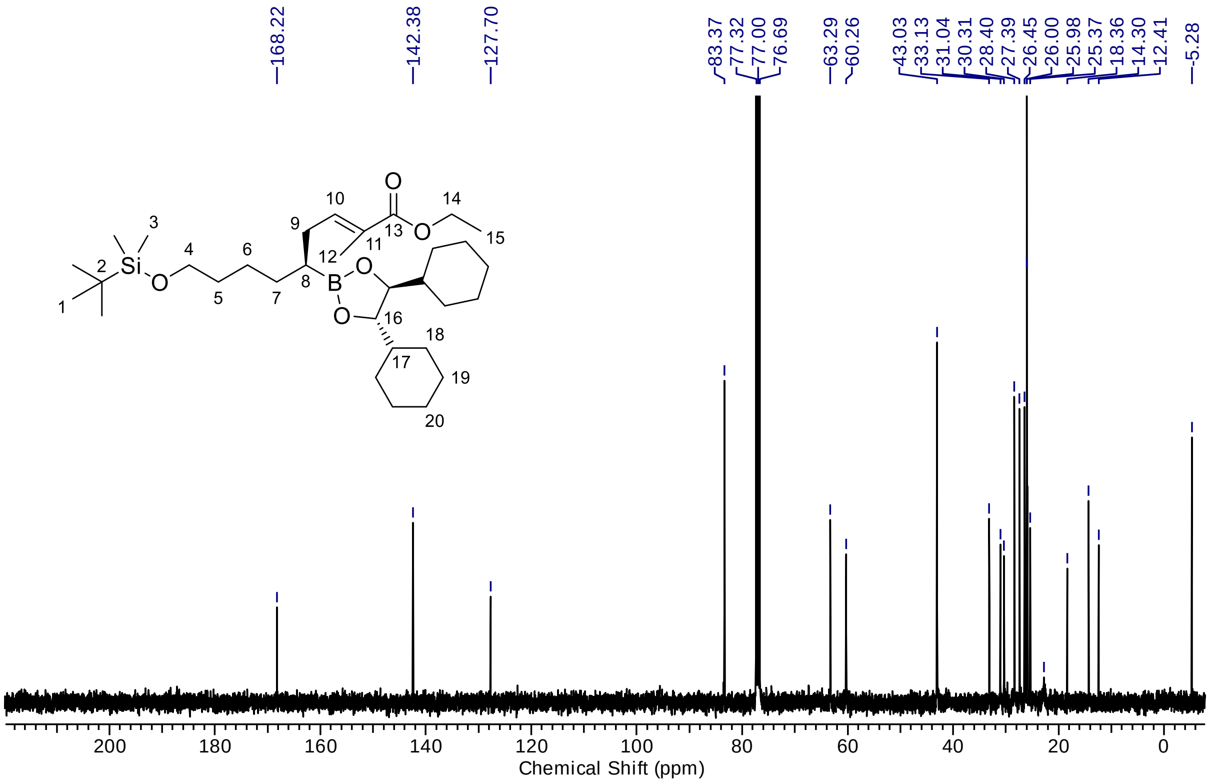

Ethyl (5*R*,6*S*,7*R*,*E*)-7-(benzyloxy)-5-[(4*S*,5*S*)-4,5-dicyclohexyl-1,3,2-dioxaborolan-2-yl]-2,6-dimethyldec-2-enoate (7d)

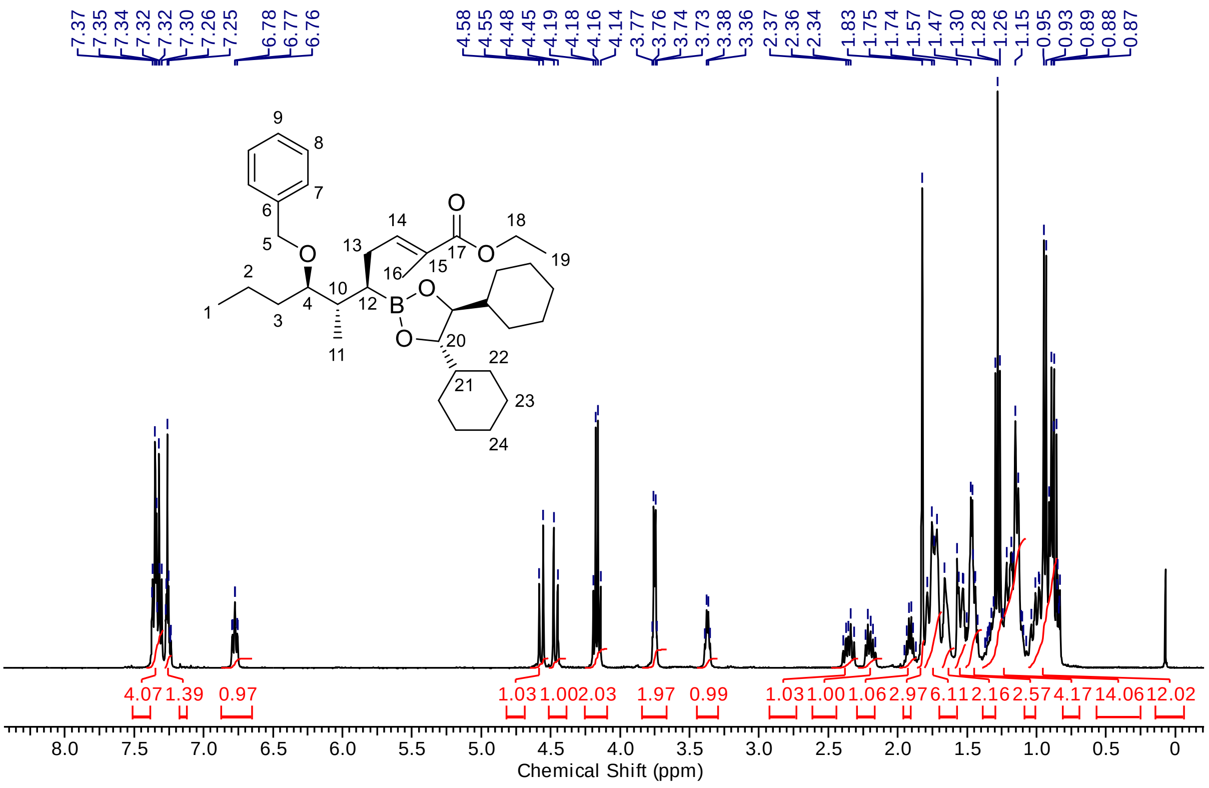

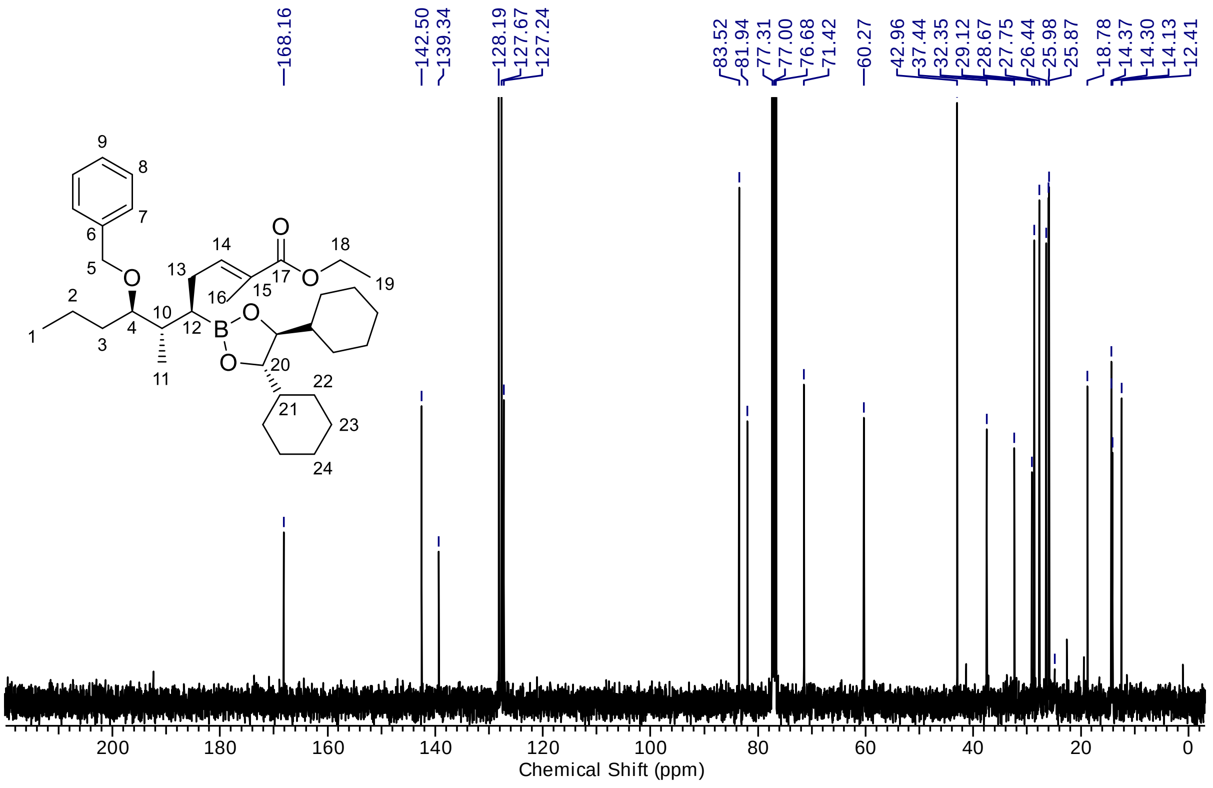

Ethyl (*R*,*E*)-5-cyclohexyl-5- [(4*S*,5*S*)-4,5-dicyclohexyl-1,3,2-dioxaborolan-2-yl]-2-methylpent-2-enoate (7e)

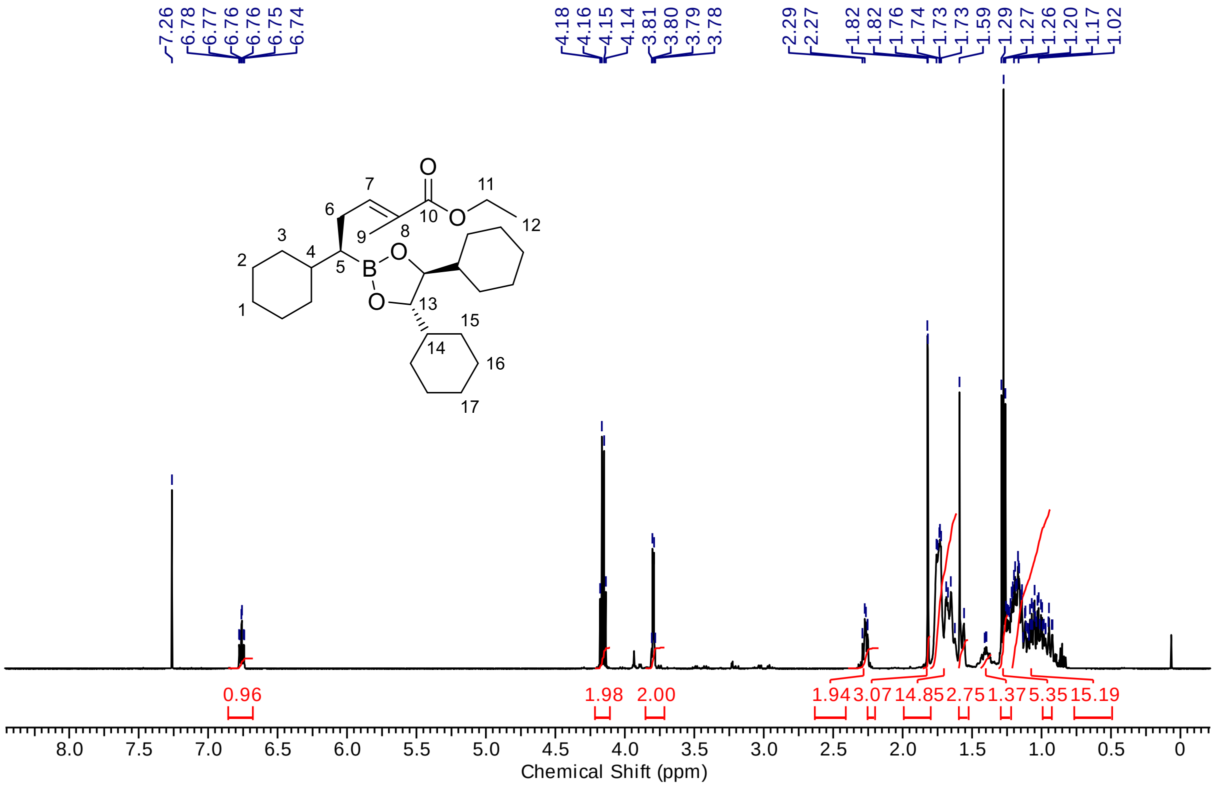

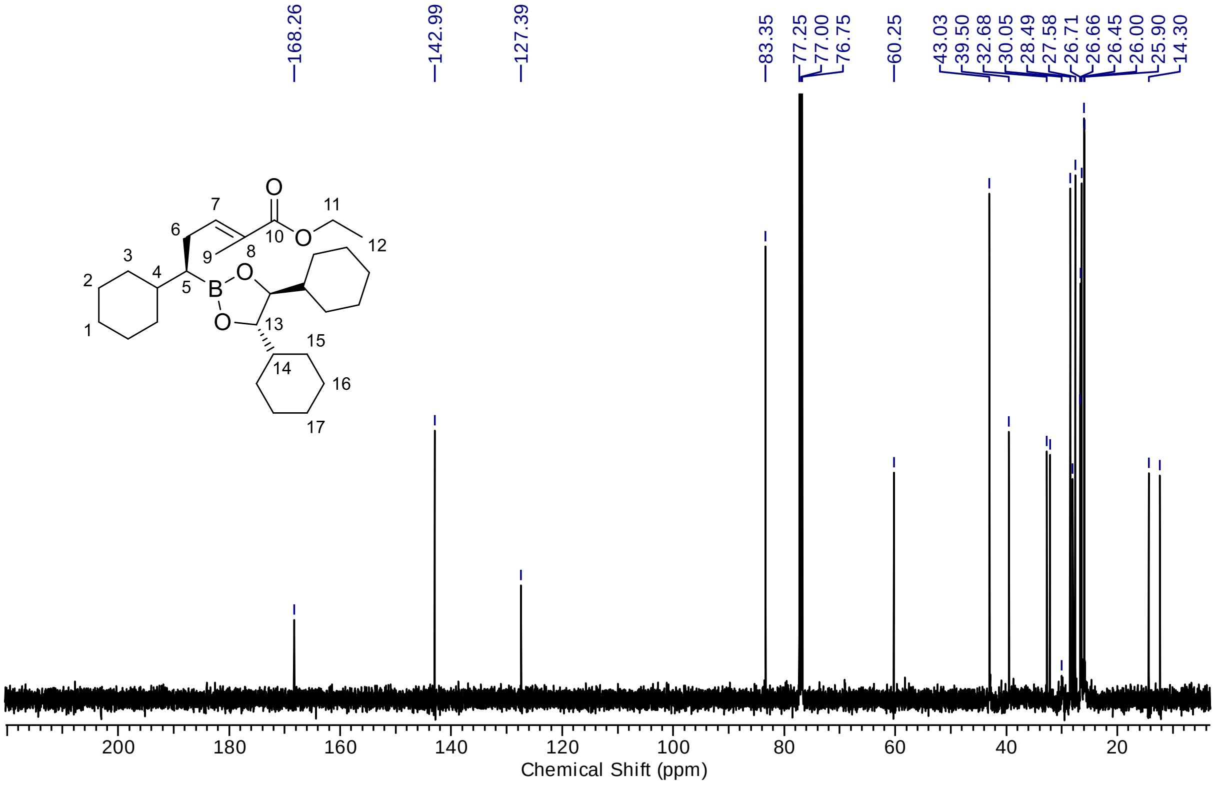

Ethyl (5R,6R,E)-5-((4S,5S)-4,5-dicyclohexyl-1,3,2-dioxaborolan-2-yl)-6-ethyl-2-methylnon-2-enoate (7f)

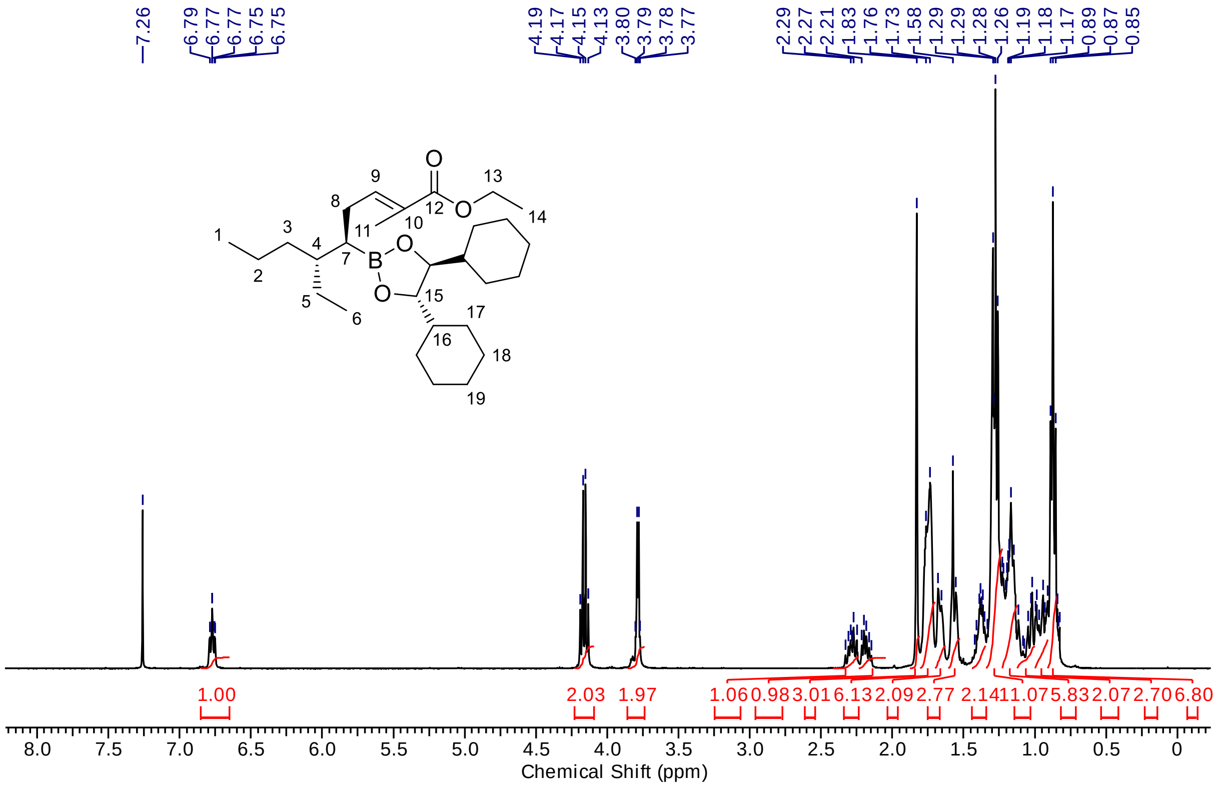

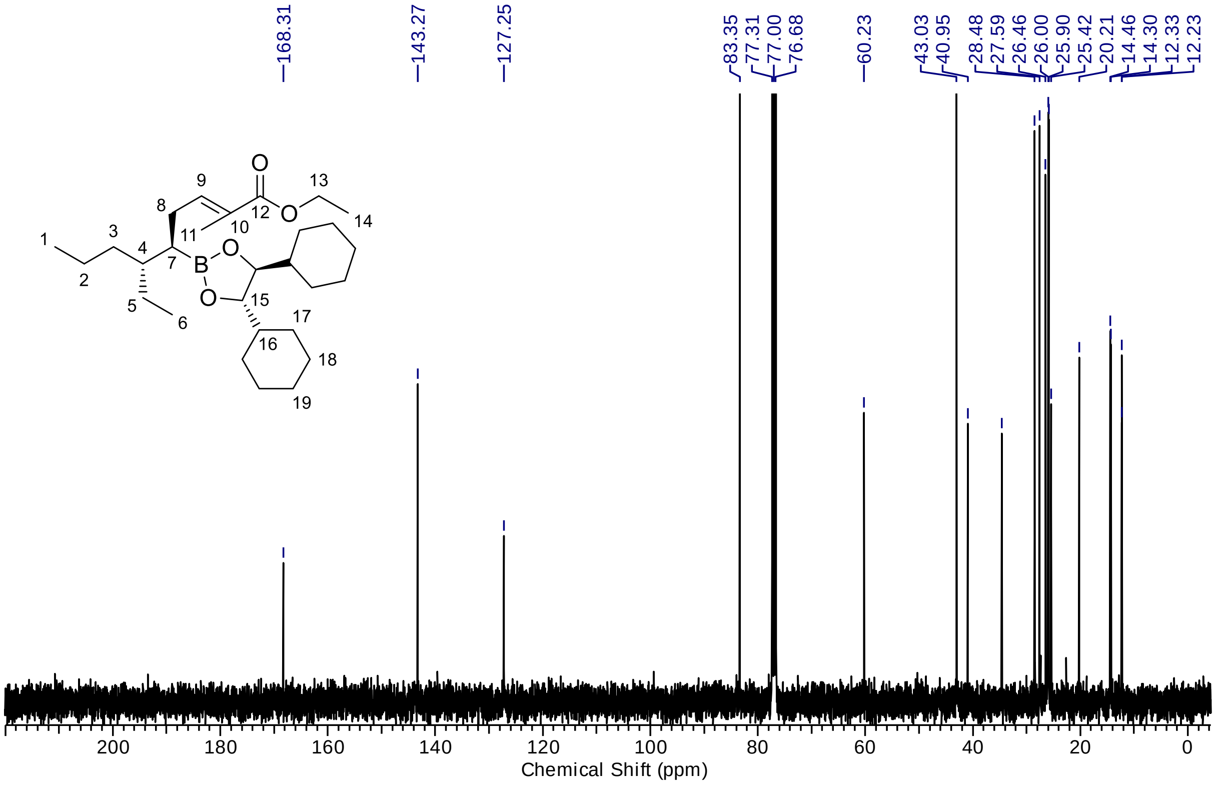

Ethyl (5*R*,6*S*,7*R*,8*S*,*E*)-7-(benzyloxy)-5-[(4*S*,5*S*)-4,5-dicyclohexyl-1,3,2-dioxaborolan-2-yl]-2,6,8-trimethyldec-2-enoate (9)

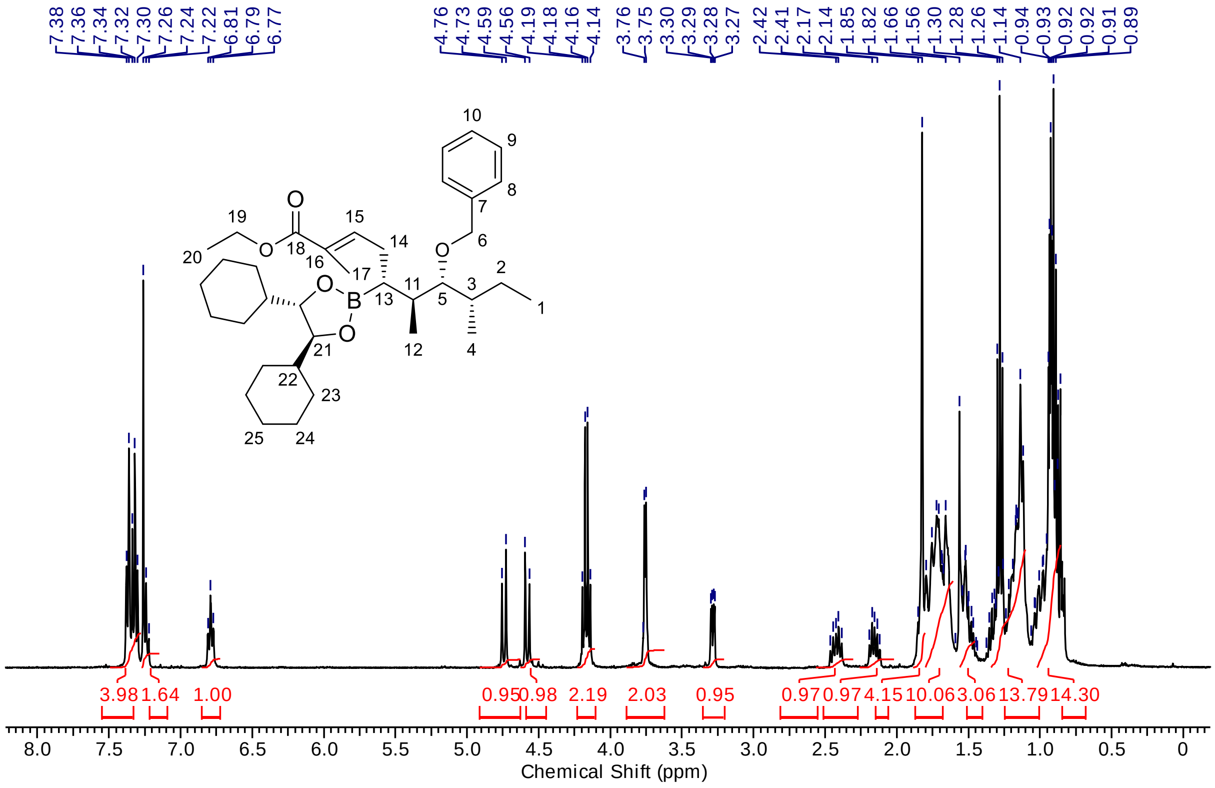

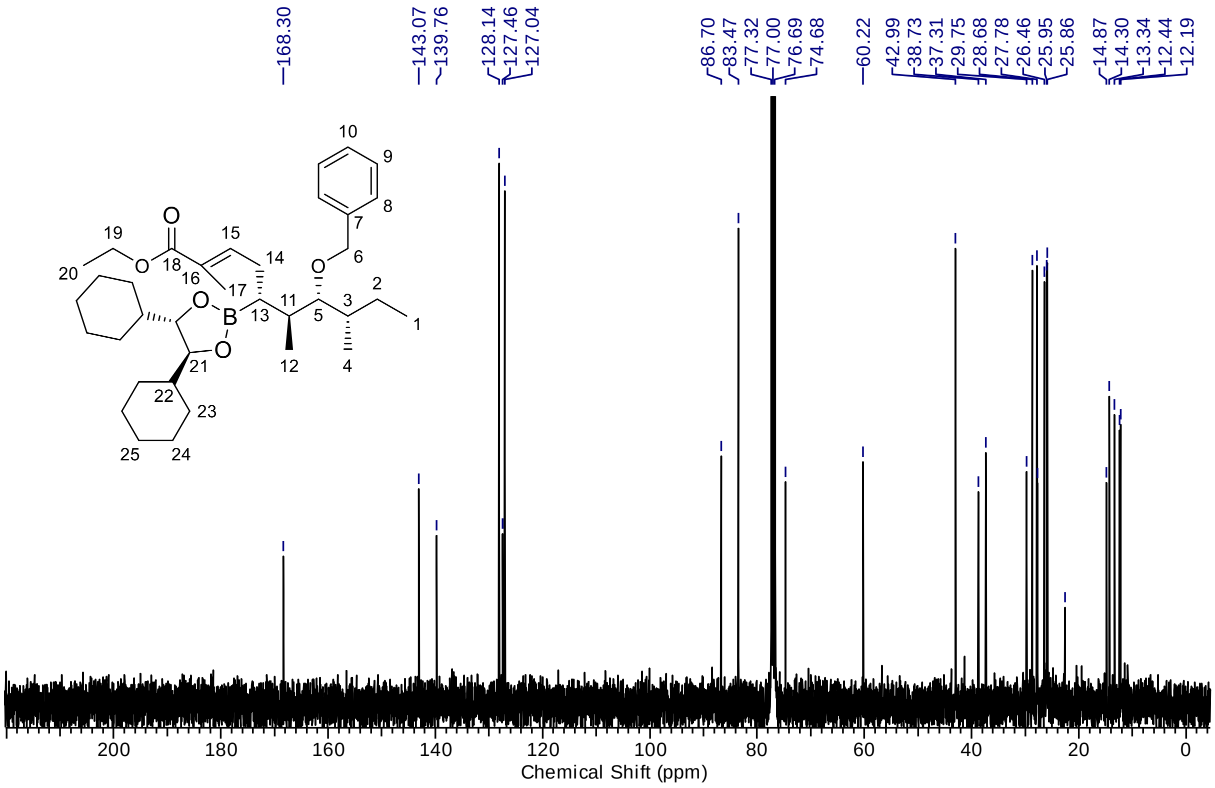

Ethyl (5*R*,6*S*,7*R*,8*S*,E)-7-(benzyloxy)-5-hydroxy-2,6,8-trimethyldec-2-enoate (10)

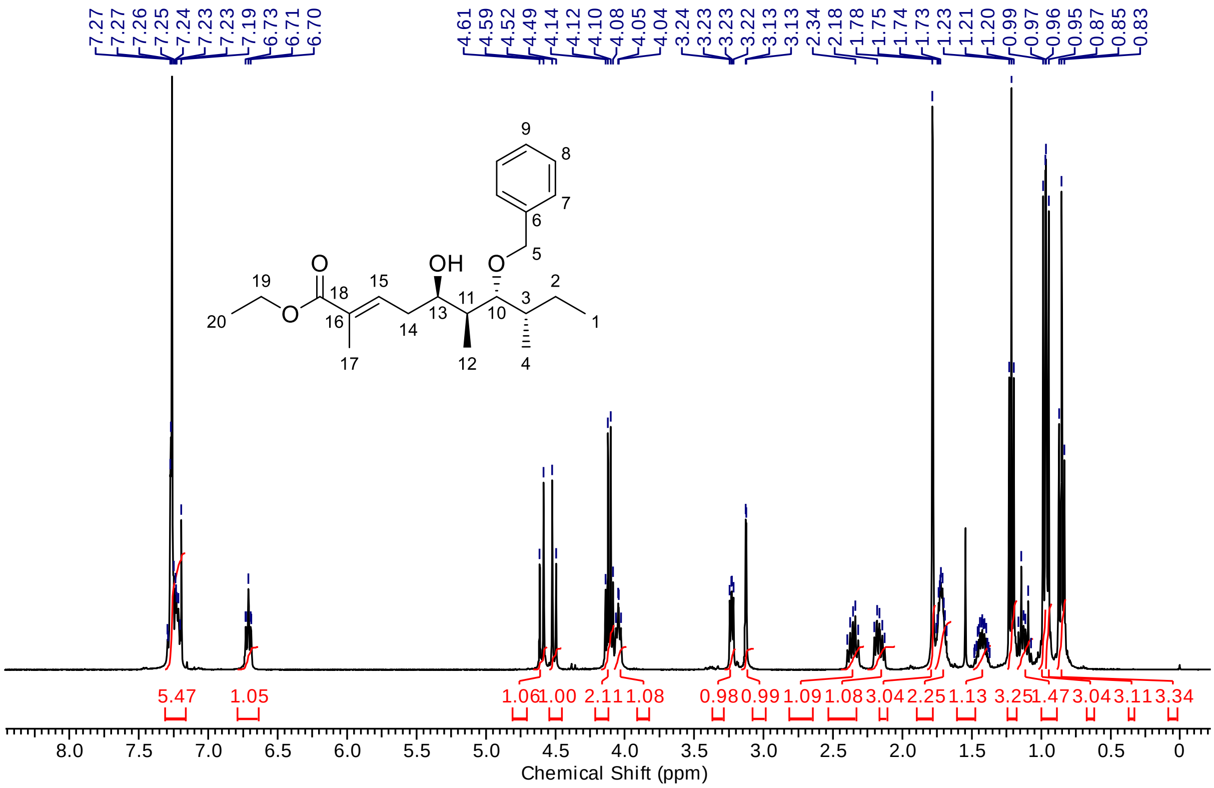

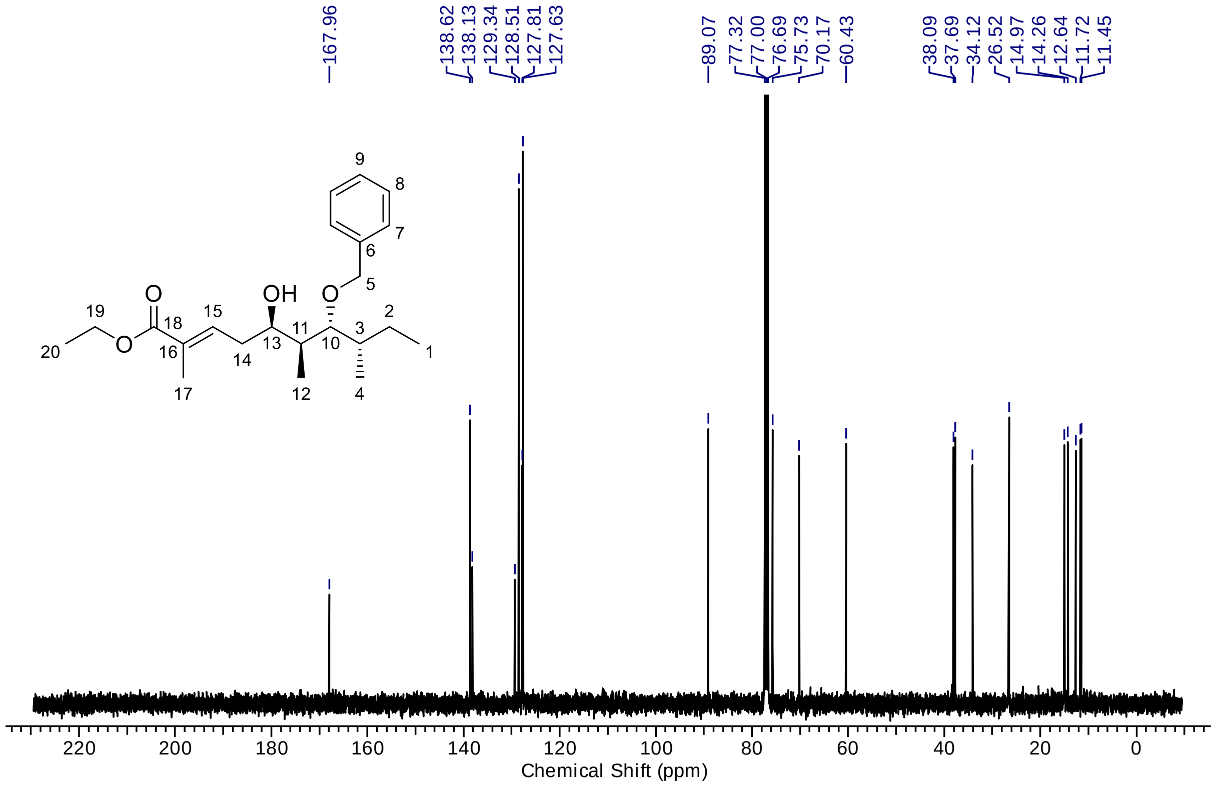

Supplement: Supplementary file 1 — Supplementary [file CHEM-27-949-s001.pdf]
